# Global, regional and national burden of endocrine, metabolic, blood and immune disorders 1990-2019: a systematic analysis of the Global Burden of Disease study 2019

**DOI:** 10.3389/fendo.2023.1101627

**Published:** 2023-05-08

**Authors:** Junyun Wu, Xiling Lin, Xin Huang, Yuyan Shen, Peng-Fei Shan

**Affiliations:** Department of Endocrinology and Metabolism, The Second Affiliated Hospital of ZheJiang University School of Medicine, Hangzhou, Zhejiang, China

**Keywords:** endocrine, metabolic, blood and immune disorders, death, disability-adjusted-life-years, years-of-life-lost, years-lived-with-disability

## Abstract

**Background:**

Endocrine, metabolic, blood and immune disorders (EMBID) is a vital public health problem globally, but the study on its burden and global trends was scarce. We aimed to evaluate the global burden of disease and trends in EMBID from 1990 to 2019.

**Methods:**

We extracted the data of EMBID-related on death cases, Age-standardized death rates (ASDRs), disability-adjusted life-years (DALYs), Age-standardized DALY rates, years of life lost (YLLs), Age-standardized YLL rates, years lived with disability (YLDs) and Age-standardized YLD rates between 1990 and 2019 from the Global Burden of Disease 2019, by sex, age, and year at the global and geographical region levels. The Annual rate of change was directly extracted from Global Health Data Exchange (GHDx) and we also calculated the age-related age-standardized rate (ASR) to quantify trends in EMBID-related deaths, DALYs, YLLs and YLDs.

**Result:**

Globally, the EMBID-related ASDRs showed an increasing trend, whereas the DALYs ASR, YLLs ASR and YLDs ASR were decreased between 1990 to 2019. Furthermore, High-income North America and Southern Sub-Saharan Africa had the highest both ASDRs and DALYs ASR, and Southern Sub-Saharan Africa and Caribbean had the highest both YLDs ASR and YLLs ASR in 2019. Males had a higher EMBID-related ASDRs than females, but the DALYs ASR in females were higher than males. The burden of EMBID was higher in older-aged compared to other age groups, especially in developed regions.

**Conclusion:**

Although EMBID-related ASRs for DALYs-, YLLs- and YLDs declined at the global level from 1990 to 2019, but the ASDRs was increasing. This implied high healthcare costs and more burden of ASDRs due to EMBID in the future. Therefore, there was an urgent need to adopt geographic targets, age-specific targets, prevention strategies and treatments for EMBID to reduce negative health outcomes globally.

## Introduction

Endocrine, metabolic, blood and immune disorders (EMBID) are a residual cause, including thyroid disorders, rare metabolic and immune disorders, and hematologic disorders that do not cause anemia (1). Previous studies have shown that approximately 13 million people in the United States, representing 4.78% of the population, suffer from undiagnosed endocrine disorders (2). Thyroid disease as one of the major endocrine disorders which accounts for approximately 30% to 40% of patients in endocrine practice. If this were true, it would account for about half of the patients with the endocrine disorders (3). Besides, another study suggested in metabolic disorders patients that nearly half (48.8%) were rare metabolic disorders, and, they assessed the general frequency of rare metabolic disorders as 10.96/10,000 individuals (4). A team demonstrated that the prevalence of immune disorders increased from 11% to nearly 16%, during 1988-1991,1992-2004, and 2011-2012 (5). Thus, EMBID as a residual cause, was also in need of attention.

EMBID is associated with a variety of adverse effects in human body. For example, hyperthyroidism caused bone loss and increased the risk of fracture, and although its effects on bone were reversible, patients had a high lifetime risk of fracture even though bone mineral density increases after treatment (6). In addition, metabolic disorders play a crucial role in the progression of tendon injury (7). Although EMBID was a group of residual cause, it’s related burden may be increased further if preventive and therapeutic measures are not taken in a timely manner. However, there were few comprehensive and up-to-date studies on the distribution of EMBID in countries worldwide.

The Global Burden of Disease (GBD) database estimates the global burden of 369 diseases and injuries, which provides favorable data for a comprehensive assessment of the distribution and trends of EMBID in each country. More comprehensive data on EMBID-related deaths, disability-adjusted life years (DALYs), years of life lost (YLLs), years lived with disability (YLDs) and respective trends in each country were necessary to allow policy makers to rationalize the allocation of resources and to formulate relevant policies more effectively depending on this knowledge, and, to date, there was barely any research on the burden associated with EMBID. Therefore, we extracted data related to EMBID in GBD for this study to illustrate the mortality rates, DALYs, YLLs, YLDs and corresponding trends in EMBID by region and country, age, sex, and socio-demographic index (SDI).

## Methods

### Overview

For this study analysis, we used data from the GBD public database 2019. GBD provides comprehensive, updated estimates of the global burden, including incidence, mortality, and prevalence of 369 diseases and injuries, as well as disability-adjusted life years by sex, age group, and 204 countries and territories. The methodological basis of GBD from 1990 to 2019 was reported in detail elsewhere. For EMBID, data were obtained through hospital discharges, claims, and data shared from the GBD collaborator network. Modeling using the Bayesian meta-regression tool, DisMod-MR, was the primary method used by GBD to generate an initial global fit on which to estimate the coefficients of the predictor variables. Previous studies demonstrated that DisMod-MR could produce more stable and valid estimates.

### Data source

Information on annual deaths, DALYs, YLLs, and YLDs and corresponding ASRs for EMBID was extracted from the publicly available Global Health Data Exchange (GHDx) (http://ghdx.healthdata.org/gbd,results,tool). We also collected on both sex- and age-related information to estimate the contribution of sex and age to the burden of EMBID. The GBD database was updated regularly and is currently available up to GBD 2019. The socio-demographic index (SDI) quantifies the number of years of development in each region. the SDI was the average of the education level for those aged 15 or older, the total fertility rate for women under 25, and per capita income, ranging from 0 to 1. GBD used the SDI to classify countries and regions into five classes: high SDI, high-middle SDI, Middle SDI, low-middle SDI, and low SDI. Geographically, the world was categorized into 21 GBD regions to assess the distribution among the different regions. Furthermore, we mapped the world including 204 countries to assess EMBID-related deaths, DALYs, YLLs and YLDs and the trend of each country from 1990 to 2019. Unlike GBD 2017, in GBD 2019, the GBD website added claims data from Poland as well as additional years of data from U.S. claims (2015-2016) and hospital discharges from Mexico, India, New Zealand, Sweden, Georgia, and Ecuador, and also incorporated hospital data from Botswana as well as data from Southern Sub-Saharan Africa.

### Definitions

Endocrine, metabolic, blood and immune disorders (EMBID) are a residual cause that includes diseases unrelated to other causes in the diabetes, genitourinary, blood and endocrine disease hierarchy. This residual group includes mainly thyroid disorders, rare metabolic and immune disorders, and hematologic disorders that do not cause anemia (specified diseases are shown in **Table S1**). EMBID is classified as asymptomatic, mild, moderate and severe categories: Asymptomatic was that the patient does not have any clinical symptoms of discomfort; Mild is low energy and feeling cold; Moderate is feeling nervous, palpitations, profuse sweating, and difficulty sleeping; Severe is easily bruised, sometimes bleeding from the gums and nose; feeling weak and having some difficulty with daily activities.

Disability-adjusted life years (DALYs) represent the sum of years lived with disability (YLDs) and years of life lost (YLLs). A disability-adjusted life year can be thought of as a lost year of “healthy” life. The sum of these disability-adjusted life years, or disease burden, in a population can be thought of as a measure of the gap between current health status and desired health status.

### Statistical analysis

The burden of EMBID was assessed using annual deaths, DALYs, YLLs, and YLDs, and the corresponding ASR (Age-standardized rate). We calculated the global EMBID-related age ASR(1-4 years, 5-14 years, 15-39 years, 40-64 years, and ≥ 65 years) and the formula calculating ASR is as follows:


$$\text{ASR }\left(\text{per }100,000\text{ population}\right)=\frac{\sum_{i=1}^{A}aiwi}{\sum_{i=1}^{A}wi}\times 100,000,$$

(*ai*, where *i* is the age-standardized rate in the ith age group, and w*i* is the count of GBD-standardized persons in the same age subgroup). We also analyzed the percentage change of deaths, DALYs, YLLs and YLDs and the corresponding ASR from 1990 to 2019, thus assessing the trend of EMBID-related burden over the 30-years period. We included all estimates in the 95% uncertainty interval (UI). R program (version 4.0.5) was used for all data analysis in this study.

## Results

### Overall status and trends in EMBID

**Figure 1:**
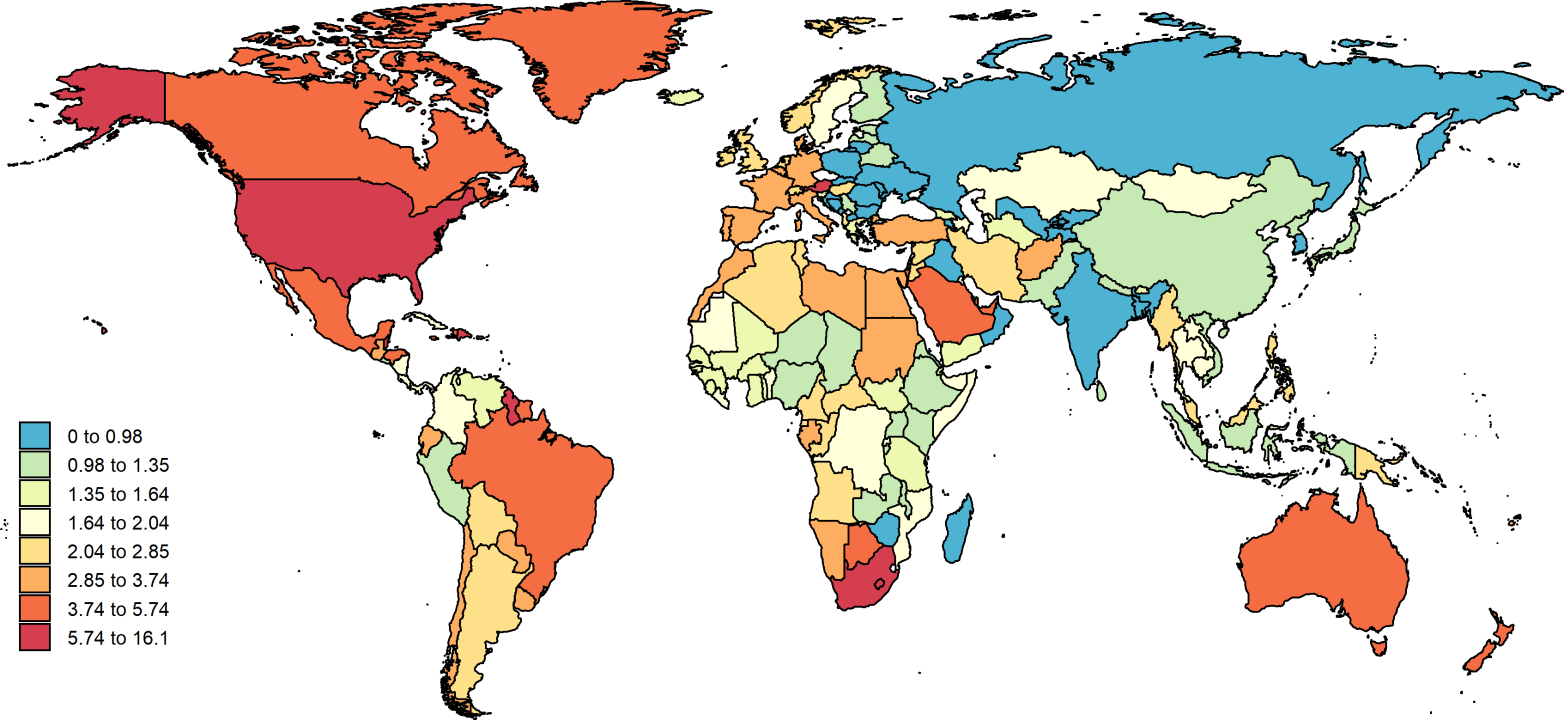
EMBID-related ASDRs by territory for both sex in 2019. EMBID, endocrine, metabolic, blood and immune disorders; ASDRs, age-standardized death rates.

In 2019, EMBID accounted for 162039.1 death cases (95% UI 131144.7-178615.4), with the Age-standardized death rates (ASDRs) 2.1per 100,000 population (95% UI 1.7-2.3). There was 108.6% (95% UI 77.9-129.1) increased in death cases and 16.1% (95% UI 1.7-24.9) increased in ASDRs from 1990. Globally, 22.9 million DALYs of EMBID (95% UI 17.0-30) were identified, with the Age-standardized rate (ASR) of DALYs 285.9 per 100,000 population (95% UI 213.0-373.3). There was an increase of 56.8% (95%UI 48.5-64.1) in DALYs, while a decrease of -4.7% (95% -8.1- -1.3) in DALYs ASR from 1990 (**Tables 1**, **2**; **Figures 1**, **S1**). There were 4.9 million YLLs of EMBID were observed, with the ASR of YLLs 64.2 per 100,000 population (95% UI 53.3-72.7). There an increase of 36.7% in YLLs and a decrease of -4.9% in the YLLs ASR from 1990. The YLDs of EMBID accounted to 18 million (95% UI 12.2-25.0), with the ASR of 221.8 per 100,000 population (95% UI 151.1-307.9). There was an increase of 63.3% in YLDs and a decrease of -4.6% in the age-standardized YLD rates, from 1990 to 2019 (**Tables 2**, **S2**; **Figures S2**, **S3**).

**Table 1:** The EMBID-related deaths, Age-standardized death rate and DALYs, Age-standardized DALY rate in 1990 and 2019.

	1990				2019			
	Deaths		DALYs		Deaths		DALYs	
	Deaths*10^3^ (95% UI)	Age-standardized death rate per 100,000 (95% UI)	DALYs *10^3^ (95% UI)	Age-standardized DALY rate per 100,000 (95% UI)	Deaths*10^3^ (95% UI)	Age-standardized death rate per 100,000 (95% UI)	DALYs*10^3^ (95% UI)	Age-standardized DALY rate per 100,000 (95% UI)
Global	77.7 (65.2, 90.1)	1.8 (1.5, 2.1)	14612.7 (10860.0, 18946.0)	300.1 (221.2, 391.8)	162.0 (131.1, 178.6)	2.1 (1.7, 2.3)	22906.6 (17001.2, 29969.5)	285.9 (213.0, 373.3)
Sex								
males	36.3 (27.9, 45.5)	1.8 (1.4, 2.2)	5383.0 (4107.5, 6890.6)	220.1 (167.8, 283.2)	76.9 (56.4, 88.5)	2.2 (1.6, 2.5)	8417.2 (6440.5, 10799.3)	216.6 (166.3, 276.8)
females	41.4 (33.0, 48.2)	1.8 (1.5, 2.1)	9229.7 (6683.9, 12084.6)	379.2 (274.1, 501.3)	85.1 (67.0, 95.2)	2.0 (1.6, 2.3)	14489.3 (10556.4, 19286.5)	354.3 (258.1, 469.0)
Age								
1-4 years	6.3 (3.9, 9.4)	1.3 (1.3, 1.9)	1031.5 (736.8, 1382.8)	206.1 (147.2, 276.3)	4.1 (3.2, 5.3)	0.8 (0.6, 1.0)	814.0 (622.9, 1053.7)	153.3 (117.3, 198.5)
5-14 years	3.7 (3.0, 4.6)	0.3 (0.3, 0.4)	1332.4 (939.4, 1881.0)	118.7 (83.6, 167.6)	3.6 (3.0, 4.5)	0.3 (0.2, 0.4)	1443.0 (1007.1, 2040.6)	114.5 (79.9, 161.8)
15-39 years	9.7 (7.6, 10.8)	0.5 (0.4, 0.5)	3703.5 (2410.4, 5613.0)	173.7 (112.9, 263.8)	13.7 (11.6, 16.1)	0.5 (0.4, 0.6)	4973.2 (3259.6, 7494.3)	176.0 (115.4, 265.2)
40-64 years	17.2 (14.2, 19.6)	1.6 (1.3, 1.8)	5193.3 (3187.0, 8084.4)	484.2 (297.3, 753.8)	41.3 (32.6, 45.9)	1.9 (1.5, 2.1)	10117.4 (6394.6, 15377.1)	459.9 (290.8, 699.0)
65 and over	28.1 (23.2, 32.4)	10.0 (8.2, 11.6)	2114.6 (1401.8, 3204.3)	645.9 (431.1, 973.2)	90.9 (68.7, 101.8)	13.5 (10.2, 15.2)	4702.6 (3282.6, 6819.8)	651.5 (456.0, 941.2)
Sociodemographic index								
High SDI	24.0 (19.9, 29.8)	2.5 (2.1, 3.1)	3201.3 (2386.0, 4158.3)	354.9 (265.3, 459.6)	63.3 (48.8, 72.3)	3.6 (2.9, 4.2)	4656.3 (3595.3, 5924.5)	354.0 (273.5, 447.9)
High-middle SDI	21.3 (16.3, 24.3)	1.6 (1.4, 1.9)	3271.2 (2411.0, 4347.4)	291.6 (215.2, 384.2)	41.2 (31.1, 46.7)	1.6 (1.4, 1.9)	4569.8 (3319.7, 6137.8)	271.0 (199.1, 358.5)
Middle SDI	15.5 (14.1, 19.2)	1.7 (1.3, 1.9)	4111.1 (3093.2, 5292.7)	276.1 (205.0, 359.7)	28.2 (23.9, 32.7)	1.9 (1.4, 2.2)	6608.8 (4876.1, 8680.2)	262.3 (195.6, 342.6)
Low-middle SDI	11.6 (8.2, 14.4)	1.3 (1.0, 1.5)	2756.5 (2043.1, 3559.8)	285.3 (207.6, 374.9)	19.7 (15.7, 22.9)	1.5 (1.2, 1.7)	4526.8 (3287.5, 5982.8)	278.8 (203.5, 367.6)
Low SDI	5.3 (3.7, 6.9)	1.3 (1.0, 1.6)	1263.5 (929.0, 1646.0)	289.1 (208.9, 381.9)	9.5 (7.3, 11.5)	1.3 (1.0, 1.6)	2531.4 (1846.3, 3336.3)	282.0 (205.4, 373.1)
Region								
Andean Latin America	1.3 (0.6, 1.6)	4.2 (2.3, 5.2)	128.3 (83.3, 162.6)	361.9 (251.8, 458.3)	1.1 (0.8, 1.8)	2.0 (1.5, 3.2)	150.3 (110.5, 200.2)	249.6 (183.9, 333.4)
Australasia	0.6 (0.5, 0.8)	2.7 (2.1, 3.4)	62.0 (47.4, 79.2)	289.6 (221.3, 369.4)	2.2 (1.7, 2.6)	4.7 (3.7, 5.7)	119.1 (94.7, 150.5)	330.3 (262.3, 413.3)
Caribbean	1.7 (1.1, 2.1)	5.7 (3.8, 6.5)	139.3 (99.4, 173.7)	411.0 (302.5, 508.8)	2.5 (2.0, 3.2)	5.2 (4.1, 6.5)	173.3 (137.2, 214.6)	365.0 (288.6, 453.5)
Central Asia	0.4 (0.3, 0.5)	0.6 (0.5, 0.8)	148.3 (109.0, 197.3)	235.2 (171.3, 312.5)	1.0 (0.7, 1.1)	1.2 (0.9, 1.4)	227.2 (167.2, 300.5)	246.0 (181.8, 324.2)
Central Europe	1.7 (1.3, 1.8)	1.4 (1.1, 1.5)	449.9 (325.9, 601.7)	348.7 (254.8, 463.1)	1.8 (1.5, 2.2)	1.1 (0.9, 1.4)	384.7 (278.9, 520.1)	274.7 (198.3, 368.4)
Central Latin America	2.7 (2.1, 3.0)	2.4 (1.9, 2.7)	325.9 (259.5, 405.5)	251.0 (195.6, 319.2)	8.4 (6.1, 9.8)	3.6 (2.6, 4.2)	698.7 (549.2, 886.5)	287.3 (225.8, 364.5)
Central Sub-Saharan Africa	1.1 (0.4, 1.8)	2.2 (1.0, 3.2)	165.7 (103.6, 238.0)	326.4 (226.0, 432.2)	1.6 (0.7, 2.4)	2.0 (0.8, 3.4)	296.1 (208.8, 400.6)	290.1 (203.1, 398.9)
East Asia	12.8 (9.6, 15.2)	1.5 (1.2, 1.7)	3133.7 (2286.7, 4133.1)	283.8 (206.2, 377.8)	19.6 (14.1, 23.0)	1.2 (0.9, 1.4)	4598.8 (3217.1, 6331.4)	249.0 (178.2, 337.6)
Eastern Europe	2.3 (1.5, 2.4)	0.9 (0.6, 1.0)	441.1 (324.7, 582.7)	186.0 (138.3, 244.7)	2.4 (2.0, 3.2)	0.9 (0.8, 1.3)	353.5 (268.0, 459.2)	154.8 (118.0, 200.6)
Eastern Sub-Saharan Africa	1.4 (1.1, 1.7)	1.2 (1.0, 1.5)	382.3 (279.9, 503.2)	273.2 (196.6, 364.2)	2.6 (2.1, 3.4)	1.3 (1.0, 1.6)	782.8 (558.1, 1058.3)	264.2 (189.0, 352.7)
High-income Asia Pacific	2.1 (1.9, 2.7)	1.3 (1.1, 1.5)	652.0 (454.9, 885.5)	346.3 (245.2, 468.8)	4.6 (3.0, 5.2)	1.0 (0.8, 1.3)	733.7 (516.3, 993.9)	304.0 (211.7, 410.8)
High-income North America	9.7 (8.2, 13.3)	2.9 (2.5, 4.0)	994.5 (770.4, 1263.1)	321.9 (250.4, 409.5)	37.2 (27.1, 40.7)	6.3 (4.8, 7.1)	1652.7 (1353.2, 1985.4)	346.0 (285.3, 417.3)
North Africa and Middle East	6.9 (4.5, 11.1)	2.4 (1.6, 3.7)	1179.3 (851.2, 1604.0)	366.3 (269.1, 479.1)	12.3 (8.7, 15.5)	2.8 (2.0, 3.5)	1995.0 (1469.1, 2606.3)	352.0 (261.0, 458.6)
Oceania	0.1 (0.1, 0.1)	3.0 (2.1, 4.1)	13.0 (9.9, 16.6)	258.6 (196.5, 336.2)	0.2 (0.2, 0.3)	3.0 (2.1, 4.2)	28.3 (21.4, 36.7)	253.9 (194.3, 332.5)
South Asia	7.3 (5.5, 9.5)	0.8 (0.7, 1.0)	2681.9 (1938.0, 3536.4)	292.5 (210.0, 389.5)	11.9 (9.7, 15.4)	0.8 (0.7, 1.1)	4930.9 (3529.8, 6615.7)	295.4 (212.0, 395.5)
Southeast Asia	5.2 (3.6, 6.2)	1.6 (1.1, 1.8)	789.3 (585.6, 1013.1)	199.9 (149.2, 258.0)	8.5 (6.8, 10.2)	1.5 (1.2, 1.8)	1149.8 (858.6, 1499.6)	167.3 (126.5, 216.3)
Southern Latin America	2.1 (1.0, 2.4)	5.0 (2.3, 5.7)	195.7 (147.1, 250.3)	409.7 (307.6, 525.0)	2.1 (1.8, 3.6)	2.7 (2.3, 4.7)	255.6 (188.4, 333.6)	349.1 (257.8, 459.4)
Southern Sub-Saharan Africa	1.7 (1.3, 2.0)	4.8 (3.6, 5.6)	223.2 (173.0, 281.9)	534.5 (410.0, 678.1)	3.5 (2.8, 4.1)	6.0 (4.6, 6.8)	373.4 (287.9, 472.2)	529.4 (408.9, 667.5)
Tropical Latin America	1.9 (1.7, 2.7)	1.8 (1.6, 2.6)	263.8 (206.9, 333.3)	206.0 (160.5, 260.7)	10.1 (6.2, 11.2)	4.4 (2.7, 4.9)	544.8 (411.9, 655.5)	236.7 (179.9, 284.1)
Western Europe	12.9 (10.5, 15.3)	2.5 (2.1, 3.1)	1793.8 (1317.7, 2363.4)	401.4 (296.3, 523.7)	24.6 (18.9, 28.8)	3.0 (2.4, 3.5)	2413.5 (1802.4, 3168.8)	415.1 (312.2, 541.0)
Western Sub-Saharan Africa	1.9 (1.2, 2.4)	1.6 (1.0, 1.9)	449.6 (325.2, 589.8)	287.7 (205.6, 383.6)	3.8 (2.8, 4.8)	1.5 (1.1, 1.8)	1044.5 (763.8, 1391.7)	291.8 (212.1, 390.9)

EMBID, endocrine, metabolic, blood and immune disorders; DALYs, disability-adjusted life years.

**Table 2:** Percent change in EMBID-related deaths, DALYs, YLLs, YLDs and corresponding ASR between 1990 to 2019.

	Percent change in number between 1990-2019 (%)				Percent change in age-standardized rate between 1990-2019 (%)			
	Deaths	DALYs	YLLs	YLDs	Deaths	DALYs	YLLs	YLDs
Global	108.6 (77.9, 129.1)	56.8 (48.5, 64.1)	36.7 (13.4, 61.8)	63.3 (59.1, 67.5)	16.1 (1.7, 24.9)	-4.7 (-8.1, -1.3)	-4.9 (-18.5, 9.5)	-4.6 (-6.1, -3.4)
Sex								
males	111.7 (77.7, 133.5)	56.4 (45.4, 67.0)	40.7 (14.1, 72.9)	64.3 (60.0, 68.8)	21.7 (7.3, 30.6)	-1.6 (-6.7, 3.2)	0.6 (-15.3, 16.3)	-2.6 (-4.3, -0.9)
females	105.9 (70.6, 142.2)	57.0 (48.6, 65.2)	32.6 (6.8, 75.0)	62.8 (58.3, 67.6)	10.7 (-4.8, 25.9)	-6.5 (-10.1, -2.6)	-10.5 (-25.7, 13.8)	-5.7 (-7.4, -4.2)
Sociodemographic index								
High SDI	164.0 (129.0, 177.4)	45.5 (39.1, 52.8)	95.7 (83.2, 102.4)	30.9 (25.6, 35.6)	44.1 (31.1, 49.4)	-0.2 (-4.0, 4.4)	28.1 (22.6, 33.7)	-9.2 (-12.0, -6.3)
High-middle SDI	82.3 (58.5, 99.0)	39.7 (31.5, 45.8)	18.7 (-4.6, 34.1)	45.0 (38.9, 51.0)	4.5 (-9.0, 13.9)	-7.1 (-11.8, -3.5)	-11.2 (-29.0, 1.7)	-5.9 (-8.5, -3.5)
Middle SDI	93.4 (59.7, 120.7)	60.8 (47.8, 72.8)	17.8 (-7.7, 43.3)	77.6 (69.2, 86.2)	9.9 (-4.2, 22.6)	-5.0 (-9.8, -0.7)	-12.7 (-28.5, 4.1)	-2.4 (-4.7, -0.2)
Low-middle SDI	70.6 (44.2, 110.4)	64.2 (50.1, 78.2)	15.8 (-8.0, 55.7)	81.2 (76.1, 86.1)	10.7 (0.1, 26.0)	-2.3 (-6.5, 1.8)	-7.6 (-23.1, 16.0)	-1.0 (-2.4, 0.3)
Low SDI	79.1 (37.9, 142.2)	100.3 (79.8, 119.0)	55.8 (12.0, 133.5)	116.0 (112.1, 119.2)	-2.2 (-14.1, 12.8)	-2.4 (-7.2, 2.1)	-10.1 (-29.4, 17.9)	-0.8 (-2.0, 0.5)
Region								
Andean Latin America	-52.4 (-69.1, 13.8)	-31.0 (-45.4, -3.1)	-63.2 (-77.6, -2.2)	-2.8 (-6.7, 1.1)	-10.2 (-42.6, 125.5)	17.1 (-12.9, 79.2)	-48.9 (-69.7, 41.5)	107.6 (95.9, 119.8)
Australasia	75.7 (57.3, 89.4)	14.1 (8.2, 22.6)	47.2 (35.7, 63.2)	-0.6 (-4.7, 3.6)	276.6 (221.5, 312.2)	92.0 (80.4, 105.9)	157.8 (139.0, 178.2)	64.4 (56.1, 72.9)
Caribbean	-8.6 (-28.0, 37.8)	-11.2 (-25.1, 12.5)	-16.9 (-37.7, 24.6)	-2.8 (-5.4, -0.5)	47.8 (16.7, 119.2)	24.4 (2.9, 59.4)	3.3 (-23.2, 57.1)	62.3 (55.5, 68.5)
Central Asia	94.6 (39.5, 124.1)	4.6 (-0.5, 10.1)	52.9 (23.5, 80.6)	-3.7 (-6.4, -1.1)	130.5 (78.5, 168.4)	53.2 (44.9, 61.2)	75.5 (45.2, 111.9)	48.2 (41.6, 54.6)
Central Europe	-23.2 (-36.0, 2.7)	-21.2 (-25.5, -15.4)	-37.0 (-49.8, -7.1)	-17.5 (-21.3, -14.2)	4.7 (-11.0, 29.0)	-14.5 (-18.9, -9.7)	-31.2 (-43.5, -7.8)	-11.2 (-15.5, -6.6)
Central Latin America	47.4 (27.1, 67.6)	14.5 (8.0, 23.0)	30.4 (12.3, 52.6)	6.1 (4.0, 8.3)	213.6 (164.8, 262.3)	114.4 (98.7, 131.2)	102.1 (71.7, 141.0)	123.0 (112.0, 134.0)
Central Sub-Saharan Africa	-11.4 (-36.4, 21.7)	-11.1 (-25.0, 1.5)	-29.9 (-55.9, 13.4)	-2.8 (6.4, 0.8)	42.5 (-15.1, 148.6)	78.6 (29.9, 131.5)	12.4 (-34.9, 132.1)	134.0 (123.2, 145.7)
East Asia	-15.3 (-32.8, 1.7)	-12.3 (-18.4, -7.0)	-37.5 (-54.0, -20.9)	-5.5 (-9.2, -1.9)	53.0 (20.2, 86.0)	46.8 (33.8, 60.0)	-21.9 (-40.0, -1.2)	65.7 (54.3, 77.7)
Eastern Europe	-0.9 (-14.1, 47.9)	-16.7 (-22.6, -5.5)	3.7 (-12.1, 51.8)	-22.9 (-28.0, -17.6)	4.3 (-10.0, 59.9)	-19.9 (-25.2, -10.2)	-3.8 (-17.3, 40.8)	-24.0 (-28.8, -19.2)
Eastern Sub-Saharan Africa	3.9 (-10.6, 23.7)	-3.3 (-7.7, 1.1)	-7.1 (-28.4, 22.1)	-2.7 (-4.1, -1.3)	88.5 (42.2, 153.9)	104.8 (86.7, 121.0)	60.0 (6.6, 147.5)	115.9 (110.8, 120.5)
High-income Asia Pacific	-17.9 (-33.7, -10.5)	-12.2 (-15.9, -9.0)	-29.5 (-38.5, -18.8)	-9.5 (-13.1, -5.9)	112.4 (42.3, 140.3)	12.5 (6.2, 18.6)	11.7 (-16.0, 24.3)	12.6 (6.0, 19.5)
High-income North America	112.6 (65.8, 126.0)	7.5 (-1.3, 17.7)	69.1 (60.9, 74.0)	-21.9 (-26.8, -16.8)	285.6 (182.4, 316.6)	66.2 (51.5, 83.8)	171.1 (138.4, 182.2)	19.5 (11.3, 27.3)
North Africa and Middle East	16.1 (-24.5, 51.3)	-3.9 (-19.4, 5.5)	-9.3 (-46.3, 26.5)	-1.7 (-3.5, 0.2)	79.3 (6.2, 145.4)	69.2 (28.9, 96.6)	20.4 (-35.1, 82.3)	98.8 (89.3, 108.2)
Oceania	2.0 (-20.5, 28.0)	-1.8 (-9.7, 7.4)	3.8 (-17.7, 31.1)	-4.8 (-9.0, -0.5)	139.0 (90.9, 198.2)	117.7 (98.2, 141.4)	128.6 (79.5, 193.4)	111.8 (100.4, 123.6)
South Asia	0.9 (-12.2, 19.0)	1.0 (-2.6, 4.5)	-8.5 (-26.7, 21.6)	2.4 (0.9, 3.8)	63.8 (35.9, 110.6)	83.9 (70.9, 95.5)	18.8 (-8.5, 71.7)	97.6 (91.7, 103.7)
Southeast Asia	-4.5 (-18.9, 15.3)	-16.3 (-21.7, -10.5)	-19.6 (-33.7, 2.5)	-14.9 (-17.7, -12.2)	65.0 (39.1, 109.2)	45.7 (31.0, 62.6)	13.8 (-10.1, 58.6)	61.9 (53.2, 70.9)
Southern Latin America	-45.8 (-56.8, 59.5)	-14.8 (-23.1, 13.0)	-37.1 (-50.4, 59.5)	-2.8 (-6.5, 1.1)	-0.1 (-19.7, 179.2)	30.6 (17.6, 70.1)	-10.5 (-28.3, 118.8)	52.8 (46.8, 58.8)
Southern Sub-Saharan Africa	24.8 (11.7, 39.4)	-1.0 (-5.4, 6.7)	0.3 (-11.7, 23.9)	-1.6 (-3.3, 0.2)	104.2 (82.1, 139.1)	67.3 (56.1, 85.7)	53.7 (29.7, 107.7)	75.5 (69.4, 81.5)
Tropical Latin America	139.6 (29.7, 173.6)	14.9 (-11.3, 33.4)	112.7 (25.2, 148.5)	-30.0 (-33.5, -26.4)	431.0 (192.5, 508.6)	106.5 (60.7, 136.5)	247.5 (107.1, 305.5)	34.8 (26.7, 42.6)
Western Europe	17.4 (1.5, 23.4)	3.4 (0.2, 6.6)	9.3 (-3.5, 18.8)	1.8 (-1.0, 4.6)	90.4 (62.7, 100.7)	34.5 (29.6, 38.9)	50.6 (30.1, 58.8)	30.7 (26.6, 34.8)
Western Sub-Saharan Africa	-7.7 (-26.4, 26.4)	1.4 (-2.8, 6.5)	-8.0 (-25.7, 21.9)	3.4 (2.0, 5.1)	96.1 (59.9, 160.3)	132.3 (118.2, 146.9)	91.8 (53.6, 153.6)	144.2 (138.4, 150.1)

EMBID, endocrine, metabolic, blood and immune disorders; DALYs, disability-adjusted life years; YLLs, years of life lost; YLDs, years lived with disability; ASR, age-standardized rate.

The EMBID-related deaths, DALYs, YLLs and YLDs were observed the highest ASR in high SDI (3.6 per 100,000 population [95% UI 2.9-4.2], 354.0 per 100,000 population [95% UI 273.5-447.9], 109.2 per 100,000 population [95% UI 90.5-138.0] and 244.8 per 100,000 population [95% UI 166.3-338.1], respectively) in 2019. Moreover, high SDI had the largest growing in terms of the ASDRs (44.1% 95 UI [31.1- 49.4]) and the ASR of YLLs (28.1% 95 UI [22.6-33.7]), while the DALYs ASR and the YLDs ASR were decreased in all SDI. Furthermore, the highest death cases and YLLs also (63292.2 [95% UI 48800.0-72255.0] and 1.4 million [95% UI 1.1-1.7], respectively) were observed in high SDI, and the highest DALYs and YLDs (6.6 million [95% UI 4.9-8.7] and 5.2 million [95% UI 3.5-7.3], respectively) were observed in middle SDI (**Tables 1**, **2**, **S2**).

**Figure 2:**
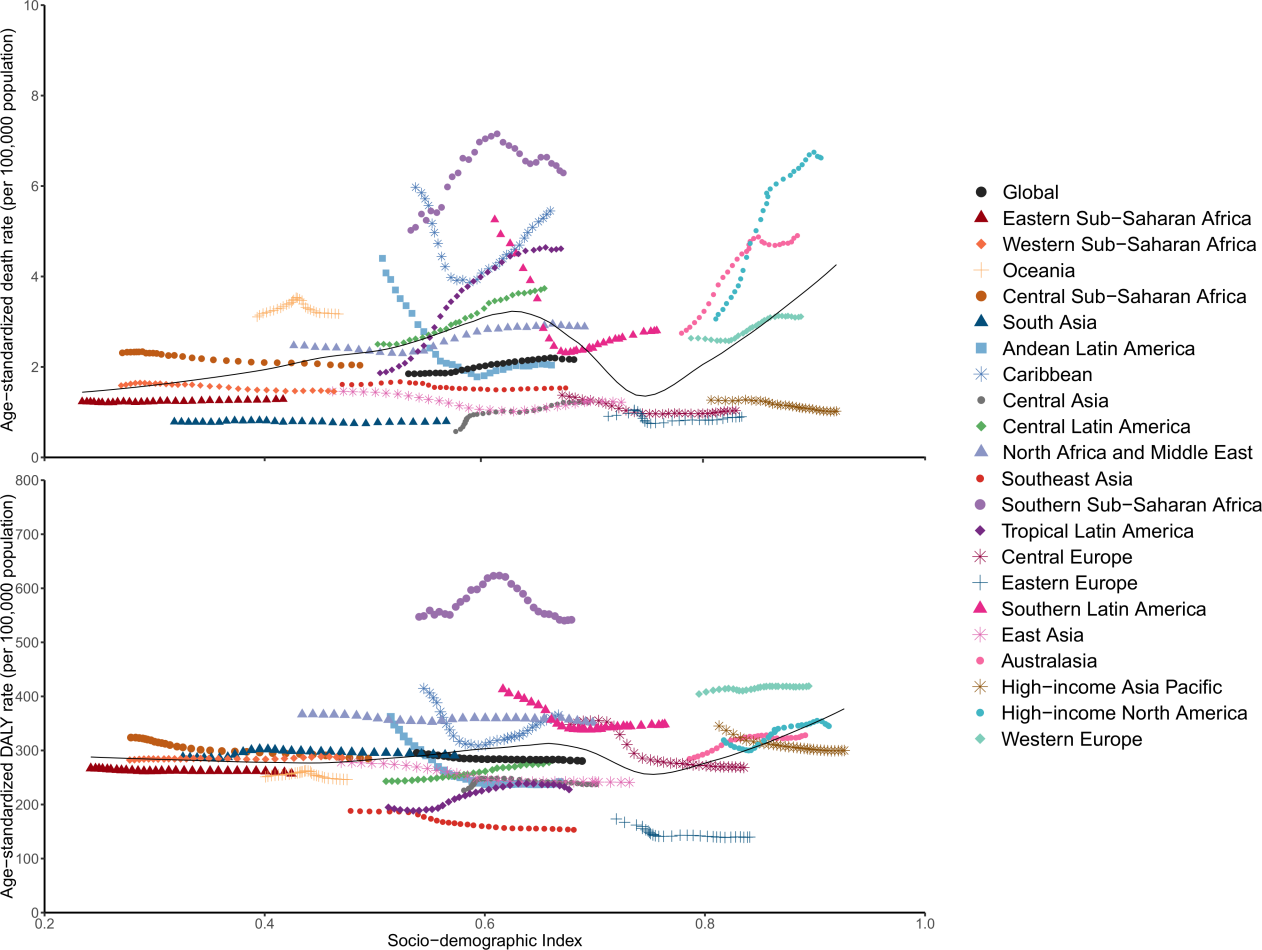
EMBID-related ASDRs and Age-standardized DALY rates across 21 GBD regions by Socio-demographic Index for both sexes combined, 1990-2019. EMBID, endocrine, metabolic, blood and immune disorders; ASDRs, age-standardized death rates; DALY, disability-adjusted life year.

The region with the EMBID-related highest ASDRs was observed in High-income North America (6.3 per 100,000 population [4.8-7.1]) and Southern Sub-Saharan Africa (6.0 per 100,000 population [4.6-6.8]) in 2019. The highest DALYs ASR was observed the Southern Sub-Saharan Africa region (529.4 per 100,000 population [95% UI 408.9-667.5]), followed by Western Europe (415.1 per 100,000 population [312.2-541.0]) (**Table 1**). Moreover, the highest YLLs ASR was observed in Caribbean (203.2 per 100,000 population [151.8-270.9]) and Southern Sub-Saharan Africa (182.3 per 100,000 population [144.4-216.6]). The Southern Sub-Saharan Africa and Western Europe identified the highest YLDs ASR (347.0 per 100,000 population [95% UI 235.3-480.0] and 320.0 per 100,000 population [95% UI 445.1-216.7], respectively) (**Table S1**). In addition, the Tropical Latin America had the largest increasing in ASDRs (431.0% UI 192.5-508.6) and YLLs ASR (247.5% UI 107.1-305.5) from 1990, and Western Sub-Saharan Africa had a largest increasing in DALYs ASR (132.3% UI 118.2-146.9) and YLDs ASR (144.2% UI 138.4-150.1) (**Table 2**; **Figures 2**, **S4**). The highest DALYs and YLDs (4.9 million [95% UI 3.5-6.6] and 4.3 million [95% UI 3.0-6.0], respectively) were identified in South Asia, and the High-income North America identified the highest death cases and YLLs (37215.7 [95% UI 27108.0-40665.2] and 830609.6 [95% UI 650204.7-972713.7], respectively).

In 2019, the country of American Samoa had the highest EMBID-related ASDRs (16.1 per 100,000 population [10.8-20.3]) and the Bahamas had the highest DALYs ASR (645.0 per 100,000 population [524.2-860.3]) (**Figures 1**, **S1**). Moreover, the highest YLLs ASR was observed in Bahamas (472.2 per 100,000 population [362.1-689.8]) and the highest YLDs was observed in Austria (445.4 per 100,000 population [303.6-615.2]) (**Figures S2**, **S3**). Between 1990 to 2019, the largest increasing of both ASDRs and YLLs ASRs were occurred in Georgia (343.5% and 281.4%, respectively), and the largest increasing of DALYs ASRs was occurred in Mexico (34.5% UI [31.5%-31.7%]), and the YLDs ASRs in Ecuador (21.5% UI [15.2%-27.9%]) was increased largest (**Tables S3**, **S4**). Furthermore, US had the highest number of both death cases and YLLs in 2019, and the highest DALYs and YLDs were identified in China.

### Men had a higher EMBID-related ASDRs than women, and women had a higher DALYs ASR than men

The ASDRs, DALYs ASR, YLLs ASR and YLDs ASR in EMBID had different trend patterns on sex. In general, males had a higher ASDRs and YLLs ASR than females, while a higher DALYs ASR and YLDs ASR were observed in females (**Figures S5**, **S6**). Globally, the number of deaths, DALYs, YLLs and YLDs grow in both males and females during past decades, reaching 76900.7, 8.4million, 2.5 million and 5.9 million in males, and 85138.5, 14.5 million, 2.4 million and 12.1 million in females (**Tables 1**, **S2**). In addition, DALYs ASR and YLDs ASR were consistently higher in women than in men throughout the study period, while the ASDR and YLLs ASR were higher in men than in women. Notably, the gap between both the ASDRs and YLLs appeared to increase over time in males and females (**Figure S7**). Furthermore, both men and women had the highest burden of ASDRs, DALY ASR, YLLs ASR and YLDs ASR in high SDI.

**Figure 3:**
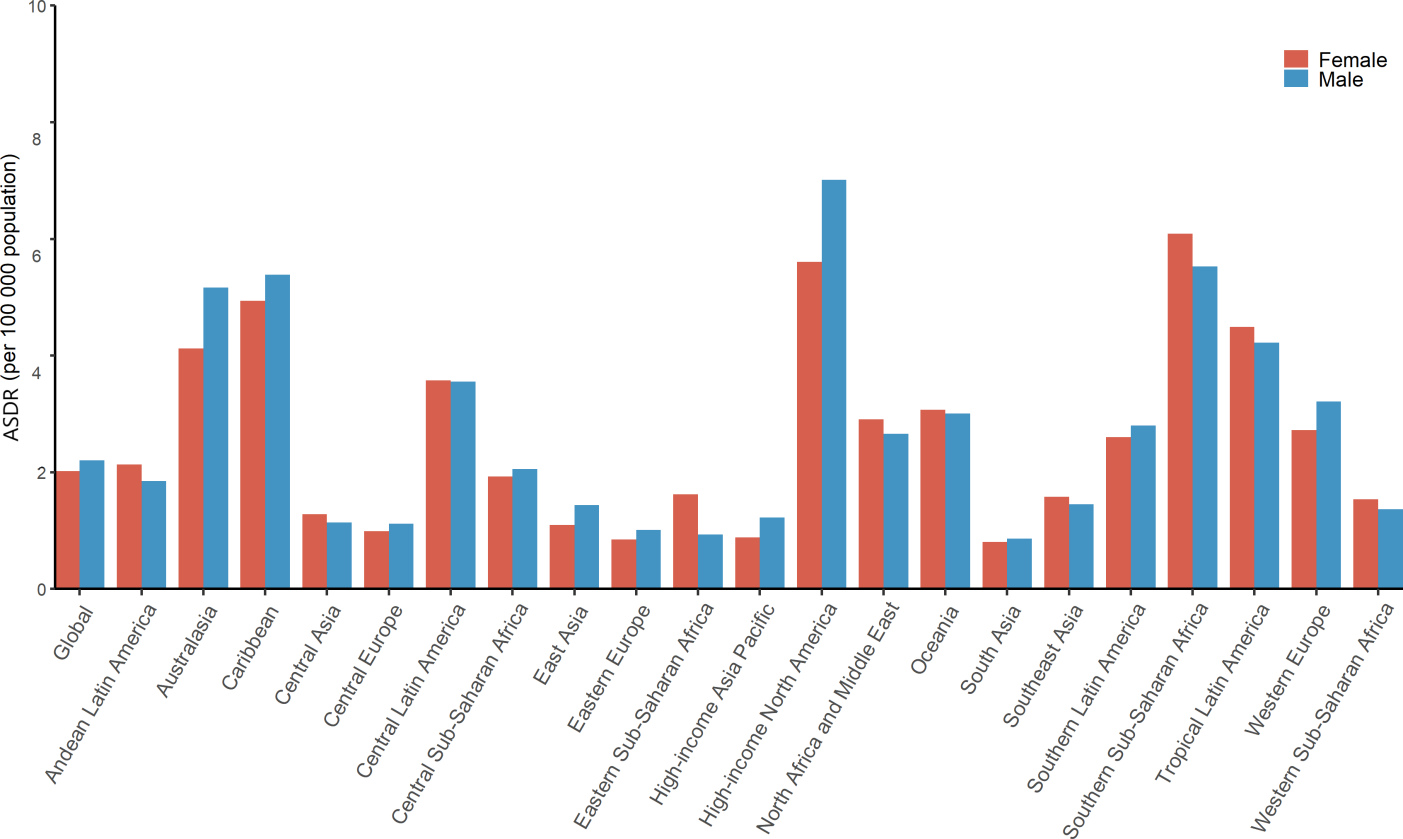
EMBID-related ASDRs for global, SDI and 21 GBD regions, by sex, in 2019. EMBID, endocrine, metabolic, blood and immune disorders; ASDRs, age-standardized death rates.

Similarly, both DALYs ASR and YLDs ASR were obviously higher for women than for men, while ASDRs and YLLs ASR were mostly higher for men than for women at the regional level. In 2019, High-income Asia Pacific had the highest ASDRs for males (7.0 per 100,000 population) and Southern Sub-Saharan Africa has the highest ASDRs for females (6.1 per 100,000 population), while South Asia has the lowest ASDRs for both sexes (**Figure 3**). In addition, the highest YLLs ASR for both males and females were found in Caribbean (**Figure S8**). Moreover, the highest DALYs ASR and YLDs in men and women were both identified in Southern Sub-Saharan Africa (**Figures S9**, **S10**). In addition, the percent change of both ASDRs and YLLs ASR increased more in in males than in females, while the percent change of both DALYs ASR and YLDs ASR increased mostly more in females than in males during the study period (**Figures S11**, **S12**). If this trend remains, the gender gap probably will widen further in the future.

### Heaviest EMBID-related burden was observed in older-aged adult population and was serious in developed regions

**Figure 4:**
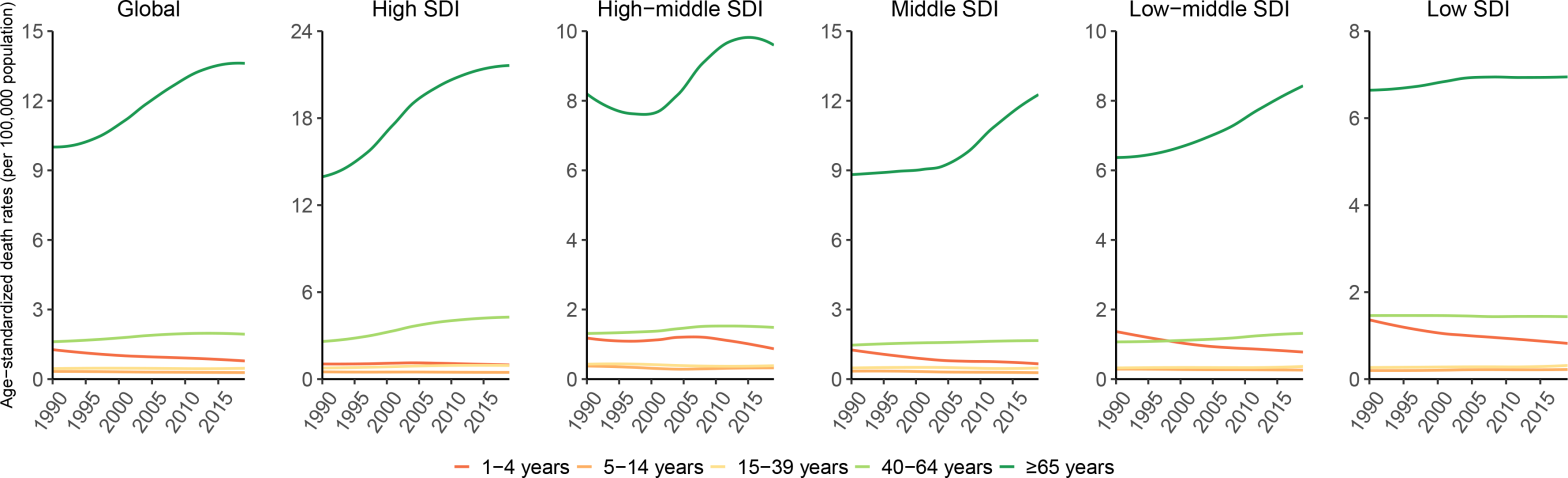
Global and SDI regions EMBID-related ASDRs by age, from 1990 to 2019. EMBID, endocrine, metabolic, blood and immune disorders; ASDRs, age-standardized death rates.

The analysis categorized the population into five age groups, namely 1-4 years (infants), 5-14 years (children), 15-39 years (youth), 40-64 years (middle-aged), and ≥ 65 years (old-aged). The global EMBID burden showed an upward trend with age, and was relatively severe in developed regions. The elderly population, especially those over 65 years aged, constituted the majority of ASDRs due to EMBID. In 2019, over 65 years aged had the highest EMBID-related ASDRs, DALYs ASR, YLLs ASR, YLDs ASR across all SDI region, followed by middle-aged. However, from 1990 to 2019, ASDRs and YLLs ASRs in both the older and middle-aged groups grew rapidly in areas with high SDI than in other SDI areas (**Figures 4**, **S13**). Moreover, the trend of DALYs ASR and YLDs ASR in each age group was relatively stable across all SDI region (**Figures S14**, **S15**). Notably, the DALYs ASR and YLDS ASR in middle-aged showed a rapid increasing trend in high-middle SDI, and the YLDs ASR was almost equal to that of older-aged in 2019. If this trend continues, the DALYs ASR and YLDs ASR of middle-aged adults are expected to exceed those of older adults in the high-middle SDI region. Besides, the YLLs ASR for infants, low SDI until 1995 and low-middle SDI until 1999, were the highest. Although trending downward from 1990 to 2019, it ranked second overall, except in the high SDI.

In addition, we analyzed and compared the percent change in each age group, across all SDI regions, between 1990 to 2019. Globally, the old-aged had the largest increase in deaths, DALYs, YLLs, and YLDs over the past decades period, with the middle-aged group being the next largest (**Figures S16**, **S17**). Furthermore, the infants showed a decreasing trend in most SDI regions. However, in addition to the increasing trend in the old-aged and middle-aged, the infants, children and youth showed a clear upward trend in the low SDI region compared to other SDI regions. For instance, in the youth, both the percent change in deaths and in DALYs showed the highest values in the low SDI regions, and the percent change in both infants and children showed a negative range in the high- and high-middle SDI regions. Thus, the burden associated with EMBID in low-SDI regions exhibits a faster increase in younger age groups compared to developed regions, and this rapid increase in the younger population is likely to continue in the future and constitute an expansion of the long-term burden of EMBID in low-SDI regions.

The age composition of those experiencing EMBID-related both deaths and YLLs between 1990 and 2019 showed a trend toward aging. The proportion of older-aged who died from EMBID in developed SDI regions was higher than the proportion of older-age who died from EMBID in developing regions. Further, Central Sub-Saharan Africa had the youngest composition of all patients who died from EMBID-related deaths Among all regions, as its younger age group accounted for 77% of deaths and 92% of YLLs in 2019. In compare, High-income Asia Pacific had the oldest composition, with older adults accounting for 83% of EMBID-related deaths and 56% of EMBID-related YLLs in 1990 and 2019 (**Figures S18**, **S19**). Compared to deaths and YLLs, the composition of DALYs and YLDs was relatively younger in all regions, with more middle-aged than older adults, followed by younger adults (**Figures S20**, **S21**). Oceania had the youngest composition of both DALYs and YLD patients, accounting for 91% of DALYs and 93% of YLDs in 2019.High-income North America had the oldest composition of DALYs and YLDs, accounting for 38% of DALYs and 34% of YLDs in 2019.

## Discussion

To our knowledge, this study was the first analysis on the burden of EMBID and its trends. This study presents updated and comprehensively available estimates of EMBID-related annual deaths, DALYs, YLLs, and YLDs for 204 countries and territories from 1990 to 2019. There were several key insights highlighted from this study. Globally, the EMBID-related ASDRs showed an increasing trend, whereas the DALYs ASR, YLLs ASR and YLDs ASR were decreased between 1990 to 2019. In general, high SDI region had the highest burden of EMBID. Furthermore, High-income North America and Southern Sub-Saharan Africa had the highest ASDRs and DALYs ASR, respectively; and Southern Sub-Saharan Africa and Caribbean had the highest YLDs ASR and YLLs ASR, respectively, in 2019. Males had a higher EMBID-related ASDRs than females, but the DALYs ASR in females were higher than males. The burden of EMBID was higher in older-aged compared to other age groups, especially in developed regions.

This study presents that EMBID-related burden was positively correlated with the level of socioeconomic development. We identified that high SDI region had the highest EMBID-related burden in 2019, especially ASDRs. From 1990 to 2019, the ASDRs was increased significantly in high SDI region. On the one hand, the reason for this may be attributed to the higher diagnostic technology and better medical system in high SDI region. For example, the United States, a country in a high SDI region, performs 118 MRI scans per 1,000 population and 245 CT scans per 1,000 population annually (8), which greatly improves the diagnosis of disease. Furthermore, a portion of high-income countries have nearly 100% of their population covered by health care, such as the United Kingdom, Australia, Canada, France, and Germany (9). This leads to more citizens being more willing to go to the hospital to visit a doctor after a health problem rising, which to the extent also increases the detection of diseases.

On the other hand, differences in EMBID-related burden between SDI levels can be attributed to the different living habits, such as dietary patterns and physical activity. Previous studies have reported that unhealthy diets, such as intake alcohol (10), sugary beverages (11), red meat (12), and fructose-rich foods (13), have long been considered dietary categories that break physical health. For example, studies have reported that alcohol abuse can lead to disturbance of the body’s vital system, the endocrine system, which in turn was related to the nervous system, the key to controlling the flow of messages between the body’s cells and organs, and that disturbance between the two can lead to disruption of many endocrine hormones in the body, such as pituitary hormones, thyroid hormones and growth hormones. In addition, alcohol consumption can even have the effect of affecting the disruption of the immune system (14). Furthermore, through the previous study has showed that the intake of sugary beverages, processed meat, and red meat was higher in high SDI region than other SDI regions (15). For instance, High-income North America, the largest contributor to EMBID-related ASDRs burden, topped the list for sugary beverage intake and processed meats (15). Other studies showed that high-income North America ranked second in terms of alcohol intake and Southern Sub-Saharan Africa has long been recognized a heavy episodic drinkers region (16, 17). So, countries should establish and strengthen the dietary guidelines, for example, by charging taxes on alcohol. In addition, insufficient physical activity and sedentary time may also be the cause of this phenomenon. Studies have reported that sedentary behavior is independently associated with poor health outcomes, while increased physical activity can be effective in improving physical health (18, 19). However, a comparative study reported that the phenomenon of insufficient physical activity increased with SDI (20). Furthermore, a study reviewed all countries around the world and compiled self-reported national-level data on sedentary time and showed that individuals in high-income countries tend to report sitting for longer periods of time than people in low-income countries (21). These results indicated that the presence of sedentary and physical inactivity was more serious in high SDI region than in low SDI region, and thus suggested that high SDI region need to make efforts in this area, such as building more public sports facilities and promoting the benefits of physical activity among the population, in order to improve the physical fitness of each citizen and reduce the occurrences of diseases.

Notably, the ASDRs of Peru has sharp declined during the past 3 decades, which could be attributed to the firm leadership of the government. In Peru, a new methodology for health system management was incorporated, which evaluated health workforce gaps depending on availability, resource requirement and estimation. They also use a matrix of modules and automatically calculate the value of the estimates. The methodology was already used in Peru, according to the Policy Guide on Human Resources for Health 2018-2030. It was used for a variety of strategic decisions, including public health intervention programs, strategies for disease prevention and control, and training plans for medical experts (22). A better health care management model can enhance the health status of the population.

In this study, we found that ASDRs was usually higher in male individuals than in female individuals, but DALYs ASR was lower than in female individuals, a phenomenon that may be due to differences in life expectancy and biological between men and women. A new WHO global report showed that women generally live longer than men, and the size of the life expectancy gap was even greater when women in low-income regions had access to better health care opportunities (23). A study claim that males live shorter yet healthier lifespans, while females tend to live longer but in relatively poorer health, possibly because much of the research on health and aging relies on physical function and disability measures to represent health (24). In addition, men and women had different behavioral factors, such as that men were more prone to perform risky behaviors, while women were more inclined to perform health-seeking behaviors (25). Our study also showed that the gap of ASDRs between men and women might continue to widen in the future. Therefore, countries should emphasize efforts to identify, investigate, and raise awareness of risky behaviors by improving their management in order to close the gender-specific gap in EMBID. Another aspect of this difference may be due to inherent, biological or related to hormonal and genetic sex differences. Women have 2 X chromosomes and can have a second X to offset the variation, while men do not, which provides a potent redundancy for women (26). The asymmetric mitochondrial maternal inheritance might benefit females by supplying noxious mutations to males, resulting in intense sex differences and survival disadvantages in males (27, 28). Furthermore, female hormones might be able to provide resistance to certain conditions, and females may have a more sensitive immune function to maintain homeostasis in the system (29, 30). Moreover, physiological differences between men and women may also be responsible for higher ASDRs in men and higher DALYs ASR in women. Arbeev et al. in the original Framingham cohort using data on heart rate, body mass index, blood pressure, hematocrit, glucose, cholesterol and mortality found faster dysregulation in women but a stronger association with risk of dysregulation and death in men (31). Cohen et al. examined five physiological systems (hepatic/renal function, oxygen transport, lipids, hematopoiesis and electrolytes) in men and women and found higher levels of dysregulation in men generally and in the hematopoietic systems and oxygen transportation (32).

We observed that older adults accounted for the highest EMBID-related burden and showed a continuous increasing trend from 1990 to 2019, which may be due to structural changes in the population and aging. This study demonstrated that the elderly population accounted for a major portion of the burden associated with EMBID and that the global age structure has shown an aging trend over the past decades, which was a strong correlation with global population aging and healthcare advances. Therefore, older adults remain the prime target groups of EMBID management in each countries (33). In addition, the middle-aged group has also shown an upward trend during the past 3 decades, occupying the second position in terms of EMBID-related burden. This phenomenon indicated that the EMBID was gradually becoming younger. In our findings, the gap between DALYs ASR and YLDs ASR in the High-middle SDI regions was gradually narrowing between older and middle-aged adults and was likely to surpass that of old-aged in the future. Our findings suggested that the trend toward a progressively younger burden of EMBID cannot be neglected, but there was a necessary and urgent need to accelerate efforts to alleviate the burden of EMBID in the older-aged. However, a study showed that four countries in the EU, including Germany, Italy, the Netherlands, and Poland, do not had health promotion policy documents that specifically addressed health promotion policies for old-aged, but that health promotion policies for older-aged can be better implemented in general policy acts that explicitly target older adults (34). Therefore, we suggested that local governments should develop more targeted health-related policies for the elderly and issue appropriate health promotion documents to loss the current highest EMBID burden of the older-aged. In addition, the literature showed that older-aged visit their doctors more often than other populations and tend to see the one doctor, which increases the possibility of physician influence (35). Then, the relevant authorities can develop policies related to health care providers on disease prevention practices, which should revolve around, for example, how to change patients’ risky behaviors and increase their awareness of health promotion and disease prevention.

Our study has the following limitations. First, because few previous studies have evaluated the burden associated with EMBID, this makes comparison with previous studies impossible. Second, original data were not collected across all countries, and estimates for these countries relied on Bayesian regression, which may result in underestimation of disease burden. Third, in the present GBD database, there was no detail on the proportion of each disease in this residual cause, which makes it impossible to assess on which disease the major focus should be.

In conclusion, EMBID-related ASRs for DALYs-, YLLs- and YLDs declined at the global level from 1990 to 2019. However, the continued global increase in ASDRs implied high healthcare costs, and the trend of aging and growth of the population will lead to more burden of ASDRs due to EMBID and, with continued advanced in medical technology, even higher costs in the future. Therefore, there was an urgent need to adopt geographic targets, age-specific targets, prevention strategies and treatments for EMBID to reduce negative health outcomes globally. Our findings will help countries to develop targeted medical policies related to EMBID.

## Data availability statement

The original contributions presented in the study are included in the article/**Supplementary Material**. Further inquiries can be directed to the corresponding author.

## Author contributions

All authors listed have made a substantial, direct, and intellectual contribution to the work and approved it for publication.

## References

[1] Vos T lim SS Abbafati C Abbas KM Abbasi M Abbasifard M. Global burden of 369 diseases and injuries in 204 countries and territories, 1990-2019: a systematic analysis for the global burden of disease study 2019. Lancet (2020) 396(10258):1204–22. doi: 10.1016/S0140-6736(20)30925-9 PMC7567026 33069326

[2] Golden SH Robinson KA Saldanha I Anton B Ladenson PW. Clinical review: prevalence and incidence of endocrine and metabolic disorders in the united states: a comprehensive review. J Clin Endocrinol Metab (2009) 94(6):1853–78. doi: 10.1210/jc.2008-2291 PMC5393375 19494161

[3] David S Cooper ECR. Thoughts on prevention of thyroid disease in the united states. THYROID (2002) 12(10):925–9. doi: 10.1089/105072502761016566 12487775

[4] Quaio C Moreira CM Chung CH Perazzio SF Dutra AP Kim CA. Frequency of carriers for rare metabolic diseases in a Brazilian cohort of 320 patients. Mol Biol Rep (2022) 49(5):3911–8. doi: 10.1007/s11033-022-07241-3 35229241

[5] Research round-up: autoimmune disease (2021). Available at: https://www.nature.com/articles/d41586-021-01834-x.

[6] Dana Jacobs-Kosmin RJD. Musculoskeletal manifestations of endocrine disorders. Curr Opin Rheumatol (2005) 17(11):64–19. doi: 10.1097/01.bor.0000150950.43282.df 15604907

[7] Abate M Schiavone C Salini V Andia I. Occurrence of tendon pathologies in metabolic disorders. Rheumatol (Oxford) (2013) 52(4):599–608. doi: 10.1093/rheumatology/kes395 23315787

[8] Papanicolas I Woskie LR Jha AK. Health care spending in the united states and other high-income countries. JAMA (2018) 319(10):1024–39. doi: 10.1001/jama.2018.1150 29536101

[9] Papanicolas I Mossialos E Gundersen A Woskie L Jha AK. Performance of UK national health service compared with other high income countries: observational study. BMJ (2019) 367:l6326. doi: 10.1136/bmj.l6326 31776110 PMC6880250

[10] Griswold MG Fullman N Hawley C Arian N Zimsen SRM Tymeson HD. Alcohol use and burden for 195 countries and territories, 1990–2016: a systematic analysis for the global burden of disease study 2016. Lancet (2018) 392(10152):1015–35. doi: 10.1016/S0140-6736(18)31310-2 PMC6148333 30146330

[11] Gan WY Mohamed SF Law LS. Unhealthy lifestyle associated with higher intake of sugar-sweetened beverages among Malaysian school-aged adolescents. Int J Environ Res Public Health (2019) 16(15). doi: 10.3390/ijerph16152785 PMC6696103 31382672

[12] Wolk A. Potential health hazards of eating red meat. J Intern Med (2017) 281(2):106–22. doi: 10.1111/joim.12543 27597529

[13] Semnani-Azad Z Khan TA Blanco Mejia S Arian N Zimsen SRM Tymeson HD. Association of major food sources of fructose-containing sugars with incident metabolic syndrome: a systematic review and meta-analysis. JAMA Netw Open (2020) 3(7):e209993. doi: 10.1001/jamanetworkopen.2020.9993 32644139 PMC7348689

[14] Nadia Rachdaoui DKS. Pathophysiology of the effects of alcohol abuse on the endocrine system. Alcohol Res (2017) 38(32):255–76. 10.35946/arcr.v38.2.08 PMC5513689 28988577

[15] Afshin A Sur PJ Fay KA Cornaby L Ferrara G Salama JS. Health effects of dietary risks in 195 countries, 1990–2017: a systematic analysis for the global burden of disease study 2017. Lancet (2019) 393(10184):1958–72. doi: 10.1016/S0140-6736(19)30041-8 PMC6899507 30954305

[16] Degenhardt L Charlson F Ferrari A Santomauro D Erskine H Mantilla-Herrara A. The global burden of disease attributable to alcohol and drug use in 195 countries and territories, 1990–2016: a systematic analysis for the global burden of disease study 2016. Lancet Psychiatry (2018) 5(12):987–1012. doi: 10.1016/S2215-0366(18)30337-7 30392731 PMC6251968

[17] Morojele NK Dumbili EW Obot IS Parry CDH. Alcohol consumption, harms and policy developments in sub-Saharan Africa: the case for stronger national and regional responses. Drug Alcohol Rev (2021) 40(3):402–19. doi: 10.1111/dar.13247 33629786

[18] Biswas A Oh PI Faulkner GE Bajaj RR Silver MA Mitchell MS. Sedentary time and its association with risk for disease incidence, mortality, and hospitalization in adults: a systematic review and meta-analysis. Ann Intern Med (2015) 162(2):123–32. doi: 10.7326/M14-1651 25599350

[19] Dimitri P Joshi K Jones N. Moving more: physical activity and its positive effects on long term conditions in children and young people. Arch Dis Child (2020) 105(11):1035–40. doi: 10.1136/archdischild-2019-318017 32198161

[20] Forouzanfar MH Afshin A Alexander LT Anderson HR Bhutta ZA Biryukov S. Global, regional, and national comparative risk assessment of 79 behavioural, environmental and occupational, and metabolic risks or clusters of risks, 1990-2015: a systematic analysis for the global burden of disease study 2015. Lancet (2016) 388(10053):1659–724. doi: 10.1016/S0140-6736(16)31679-8 PMC5388856 27733284

[21] McLaughlin M Atkin AJ Starr L Hall A Wolfenden L Sutherland R. Worldwide surveillance of self-reported sitting time: a scoping review. Int J Behav Nutr Phys Act (2020) 17(1):111. doi: 10.1186/s12966-020-01008-4 32883294 PMC7469304

[22] Mabunda SA Gupta M Chitha WW Mtshali NG Ugarte C Echegaray C. Lessons learnt during the implementation of WISN for comprehensive primary health care in India, south Africa and Peru. Int J Environ Res Public Health (2021) 18(23). doi: 10.3390/ijerph182312541 PMC8656745 34886270

[23] Thornton J. WHO report shows that women outlive men worldwide. BMJ (2019) 365:l1631. doi: 10.1136/bmj.l1631 30952650

[24] Hazzard WR. Why do women live longer than men? biologic differences that influence longevity. Postgrad Med (1989) 85(85):271–278, 281-273. doi: 10.1080/00325481.1989.11700672 2648373

[25] Rogers RG E BG Saint Onge JM Krueger PM. Social, behavioral, and biological factors, and sex differences in mortality. Demography (2010) 47(43):555–78. doi: 10.1353/dem.0.0119 PMC3000060 20879677

[26] Austad SN Fischer KE. Sex differences in lifespan. Cell Metab (2016) 23(6):1022–33. doi: 10.1016/j.cmet.2016.05.019 PMC4932837 27304504

[27] Wolff JN Gemmell NJ. Mitochondria, maternal inheritance, and asymmetric fitness: why males die younger. Bioessays (2013) 35(2):93–9. doi: 10.1002/bies.201200141 23281153

[28] Frank SA H LD. Mitochondria and male disease. Nature (1996) 383(6597):6224. doi: 10.1038/383224a0 8805695

[29] Perez-Lopez FR Larrad-Mur L Kallen A Chedraui P Taylor HS. Gender differences in cardiovascular disease: hormonal and biochemical influences. Reprod Sci (2010) 17(6):511–31. doi: 10.1177/1933719110367829 PMC3107852 20460551

[30] Pomatto LCD Tower J Davies KJA. Sexual dimorphism and aging differentially regulate adaptive homeostasis. J Gerontol A Biol Sci Med Sci (2018) 73(2):141–9. doi: 10.1093/gerona/glx083 PMC5861879 28525535

[31] Arbeev KG Cohen AA Arbeeva LS Milot E Stallard E Kulminski AM. Optimal versus realized trajectories of physiological dysregulation in aging and their relation to sex-specific mortality risk. Front Public Health (2016) 4:3. doi: 10.3389/fpubh.2016.00003 26835445 PMC4725219

[32] Cohen AA Legault V Li Q Fried LP Ferrucci L. Men sustain higher dysregulation levels than women without becoming frail. J Gerontol A Biol Sci Med Sci (2018) 73(2):175–84. doi: 10.1093/gerona/glx146 PMC5861919 28977345

[33] Bennett JE Stevens GA Mathers CD Bonita R Rehm J Kruk ME. NCD countdown 2030: worldwide trends in non-communicable disease mortality and progress towards sustainable development goal target 3.4. Lancet (2018) 392(10152):1072–88. doi: 10.1016/S0140-6736(18)31992-5 30264707

[34] Arsenijevic J Groot W. Health promotion policies for elderly-some comparisons across Germany, Italy, the Netherlands and Poland. Health Policy (2022) 126(1):69–73. doi: 10.1016/j.healthpol.2020.01.013 32113665

[35] The aging population in the twenty-first century: statistics for health policy. Available at: https://www.ncbi.nlm.nih.gov/books/NBK217727/. 25032446

