# Supplementary material for: Global, regional and national burden of endocrine, metabolic, blood and immune disorders 1990-2019: a systematic analysis of the Global Burden of Disease study 2019
Source: Front Endocrinol (Lausanne). 2023 May 8;14:1101627. doi: 10.3389/fendo.2023.1101627 (PMC10200867; doi:10.3389/fendo.2023.1101627)
Supplement: Supplementary file 1 [file DataSheet_1.pdf]

Table S1 List of EMBID-related disease based on ICD-10.

ICD10	Disease
D52.1	Drug-induced folate deficiency anaemia
D59.0	Drug-induced autoimmune haemolytic anaemia
D59.2	Drug-induced nonautoimmune haemolytic anaemia
D59.6	aemoglobinuria due to haemolysis from other external causes
D66	Hereditary factor VIII deficiency
D67	Hereditary factor IX deficiency
D68	Other coagulation defects
D69	Purpura and other haemorrhagic conditions
D70	Agranulocytosis
D70.0	Congenital agranulocytosis
D70.1	Agranulocytosis secondary to cancer chemotherapy
D70.2	Other drug-induced agranulocytosis
D70.4	Cyclic neutropenia
D70.8	Other neutropenia
D70.9	Neutropenia, unspecified
D71	Functional disorders of polymorphonuclear neutrophils
D72	Other disorders of white blood cells
D73	Diseases of spleen
D74	Methaemoglobinaemia
D75	Other diseases of blood and blood-forming organs
D75.0	Familial erythrocytosis
D75.1	Secondary polycythaemia
D75.8	Other specified diseases of blood and blood-forming organs

D76	Other specified diseases with participation of lymphoreticular and reticulohistiocytic tissue
D77	Other disorders of blood and blood-forming organs in diseases classified elsewhere
D78	Disorders of lipoprotein metabolism and other lipidaemias
D78.0	Pure hypercholesterolaemia
D78.1	Pure hyperglyceridaemia
D78.2	Mixed hyperlipidaemia
D78.3	Hyperchylomicronaemia
D78.4	Other hyperlipidaemia
D78.5	Hyperlipidaemia, unspecified
D78.6	Lipoprotein deficiency
D78.8	Other disorders of lipoprotein metabolism
D86.8	Sarcoidosis of other sites
D89	Other disorders involving the immune mechanism, not elsewhere classified
D89.0	Polyclonal hypergammaglobulinaemia
D89.1	Cryoglobulinaemia
D89.2	Hypergammaglobulinaemia, unspecified
E03	Other hypothyroidism
E04	Other nontoxic goitre
E05	Thyrotoxicosis hyperthyroidism
E06	Thyroiditis
E07	Other disorders of thyroid
E07.0	Hypersecretion of calcitonin
E07.1	Dyshormogenetic goitre
E09	Drug or chemical induced diabetes mellitus
E15.0	Nondiabetic hypoglycaemic coma
E16.0	Other disorders of pancreatic internal secretion

E20	Hypoparathyroidism
E21	Hyperparathyroidism and other disorders of parathyroid gland
E22	Hyperfunction of pituitary gland
E23	Hypofunction and other disorders of pituitary gland
E24	Cushing syndrome
E24.0	Pituitary-dependent Cushing disease
E24.1	Nelson syndrome
E24.2	Drug-induced Cushing syndrome
E24.3	Ectopic ACTH syndrome
E24.8	Other Cushing syndrome
E24.9	Cushing syndrome, unspecified
E25	Adrenogenital disorders
E26	Hyperaldosteronism
E27	Other disorders of adrenal gland
E28.0	Estrogen excess
E28.1	Androgen excess
E28.3	Primary ovarian failure
E28.8	Other ovarian dysfunction
E28.9	Ovarian dysfunction, unspecified
E34	Other endocrine disorders
E36	Bacterial sepsis of newborn
E65	Localized adiposity
E66	Obesity
E67	Other hyperalimentation
E68	Sequelae of hyperalimentation
E70	Disorders of aromatic amino-acid metabolism

E71	Disorders of branched-chain amino-acid metabolism and fatty-acid metabolism
E72	Other disorders of amino-acid metabolism
E73	Lactose intolerance
E74	Other disorders of carbohydrate metabolism
E75	Disorders of sphingolipid metabolism and other lipid storage disorders
E76	Disorders of glycosaminoglycan metabolism
E77	Disorders of glycoprotein metabolism
E78	Disorders of lipoprotein metabolism and other lipidaemias
E79	Disorders of purine and pyrimidine metabolism
E80	Disorders of porphyrin and bilirubin metabolism
E82	Immunodeficiency associated with other major defects
E83	Disorders of mineral metabolism
E84	Cystic fibrosis
E85	Amyloidosis
E85.0	Non-neuropathic heredofamilial amyloidosis
E85.1	Neuropathic heredofamilial amyloidosis
E85.2	Heredofamilial amyloidosis, unspecified
E88	Other metabolic disorders
E89	Postprocedural endocrine and metabolic disorders, not elsewhere classified
G21.0	Malignant neuroleptic syndrome
G21.1	Other drug-induced secondary parkinsonism
G24.0	Drug-induced dystonia
G25.1	Drug-induced tremor
G25.4	Drug-induced chorea
G25.6	Drug-induced tics and other tics of organic origin
G25.7	Other and unspecified drug induced movement disorders

G72	Other myopathies
G93.7	Reye syndrome
G97	Postprocedural disorders of nervous system, not elsewhere classified
I95.2	Hypotension due to drugs
I95.3	Hypotension of hemodialysis
I97	Postprocedural disorders of circulatory system, not elsewhere classified
I98.9	Disorder of circulatory system, unspecified
J70.0	Acute pulmonary manifestations due to radiation
J70.1	Chronic and other pulmonary manifestations due to radiation
J70.2	Acute drug-induced interstitial lung disorders
J70.3	Chronic drug-induced interstitial lung disorders
J70.4	Drug-induced interstitial lung disorders, unspecified
J70.5	Respiratory conditions due to smoke inhalation
J95	Postprocedural respiratory disorders, not elsewhere classified
K43	Ventral hernia
K52.0	Gastroenteritis and colitis due to radiation
K62.7	Radiation proctitis
K91	Postprocedural disorders of digestive system, not elsewhere classified
K94	Colostomy complication, unspecified
K95	Complications of bariatric procedures
M87.1	Osteonecrosis due to drugs
N14	Drug- and heavy-metal-induced tubulo-interstitial and tubular conditions
N14.0	Analgesic nephropathy
N14.1	Nephropathy induced by other drugs, medicaments and biological substances
N14.2	Nephropathy induced by unspecified drug, medicament or biological substance
N14.3	Nephropathy induced by heavy metals

N14.4	Toxic nephropathy, not elsewhere classified
N65	Deformity and disproportion of reconstructed breast
N65.0	Obstructed labour due to deformed pelvis
N65.1	Obstructed labour due to generally contracted pelvis
N99	Postprocedural disorders of genitourinary system, not elsewhere classified
P96.2	Withdrawal symptoms from therapeutic use of drugs in newborn
P96.5	Complications of intrauterine procedures, not elsewhere classified
R50.2	Drug-induced fever

EMBID: endocrine, metabolic, blood and immune disorders.

Table S2: The EMBID-related YLLs, Age-standardized YLL rate and YLDs, Age-standardized YLD rate in 1990 and 2019.

	**1990**				**2019**			
	**YLLs**		**YLDs**		**YLLs**		**YLDs**	
	YLLs*10^3 (95% UI)	Age-standardized YLL rate per 100,000 (95% UI)	YLDs*10^3 (95% UI)	Age-standardized YLD rates per 100,000 (95% UI)	YLLs*10^3 (95% UI)	Age-standardized YLL rate per 100,000 (95% UI)	YLDs*10^3 (95% UI)	Age-standardized YLD rate per 100,000 (95% UI)
Global	3590.2 (2874.5, 4329.9)	67.5 (55.4, 80.2)	11022.4 (7513.6, 15340.3)	232.6 (158.5, 323.1)	4906.3 (4066.1, 5534.5)	64.2 (53.3, 72.7)	18000.3 (12249.6, 24962.9)	221.8 (151.1, 307.9)
**Sex**								
males	1810.7 (1249, 2410.5)	67.8 (49.6, 86.7)	3572.3 (2416.9, 4992.7)	152.3 (103.6, 212.9)	2547.3 (1954.8, 3013.7)	68.2 (52.3, 80.9)	5869.9 (3977.5, 8163.4)	148.4 (100.6, 206.4)
females	1779.6 (1277.9, 2181.4)	67.1 (49.7, 80.3)	7450.1 (5082.5, 10360.5)	312.0 (212.4, 431.3)	2359.0 (1922.3, 2717.1)	60.1 (49.4, 69.5)	12130.4 (8240.3, 16800.8)	294.2 (199.8, 406.4)
**Age**								
1-4 years	5444.6 (3358.3, 8054.8)	108.8 (67.1, 160.9)	4870.7 (3137.3, 7140.4)	97.3 (62.7, 142.7)	3543.9 (2772.3, 4520.0)	66.8 (52.2, 85.1)	4595.6 (2924.3, 6794.4)	86.6 (55.1, 128.0)
5-14 years	2955.5 (2345.8, 3649.5)	26.3 (20.9, 32.5)	10368.0 (6428.8, 15793)	92.4 (57.3, 140.7)	2870.1 (2400.2, 3563.7)	22.8 (19.0, 28.3)	11560.3 (7147.6, 17554.8)	91.7 (56.7, 139.2)

15-39 years	5886.7 (4587.3, 6551.6)	27.2 (21.2, 30.3)	31148.4 (18178.2, 50126.5)	146.5 (85.5, 235.9)	8149.7 (6863.3, 9570.0)	28.8 (24.2, 33.8)	41582.3 (24469.9, 66789.0)	147.2 (86.7, 236.4)
40-64 years	6138.2 (5028.2, 6973.5)	57.2 (46.9, 65.1)	45794.4 (25860.5, 74486.1)	426.9 (241.2, 694.5)	14507.6 (11486.1, 16143.0)	66.2 (52.4, 73.5)	86666 (49762.1, 139298.5)	393.8 (226.1, 633.0)
65 and over	4304.8 (3590.1, 4975.1)	139.1 (115.4, 160.7)	16841.7 (9723.0, 27742.3)	506.9 (292.7, 834.1)	12588.0 (9751.1, 14006.3)	179.4 (138.3, 199.8)	34438.1 (20418.9, 55382.2)	472.1 (279.8, 758.4)
**Sociodemographic index**								
High SDI	720.0 (598.3, 900.9)	85.3 (71.3, 106.5)	2481.3 (1677.3, 3436.1)	269.6 (181.5, 373.3)	1408.6 (1128.0, 1697.0)	109.2 (90.5, 138.0)	3247.6 (2208.9, 4475.7)	244.8 (166.3, 338.1)
High-middle SDI	664.3 (591.2, 870.3)	61.9 (54.9, 81.8)	2606.9 (1759.1, 3636.6)	229.6 (154.9, 320.6)	788.5 (664.1, 921.7)	55.0 (46.9, 65.1)	3781.3 (2542.7, 5320.8)	216.0 (145.3, 301.8)
Middle SDI	1157.1 (827.3, 1399.9)	68.7 (52.0, 81.0)	2954.1 (1996.7, 4131.8)	207.3 (140.0, 289.9)	1363.0 (1073.7, 1549.5)	60.0 (47.3, 68.4)	5245.8 (3518.2, 7310.5)	202.3 (136.0, 282.0)
Low-middle SDI	716.2 (480.8, 946.9)	55.5 (38.8, 69.8)	2040.3 (1380.7, 2829.4)	229.8 (156.0, 316.5)	829.0 (651.7, 988.6)	51.3 (40.3, 60.7)	3697.7 (2505.9, 5130.4)	227.5 (154.5, 314.8)
Low SDI	328.5 (212.6, 463.5)	52.0 (36.6, 66.4)	935.1 (634.0, 1296.9)	237.1 (161.9, 328.4)	511.9 (387.5, 644.4)	46.8 (35.8, 57.1)	2019.5 (1356.8, 2820.5)	235.2 (160.5, 327.1)
**Region**								
Andean Latin America	74.2 (35.4, 97.8)	169.4 (86.1, 216.5)	54.1 (37.0, 75.1)	192.5 (132.4, 268.0)	37.9 (27.1, 64.0)	62.4 (44.8, 104.5)	112.4 (76.2, 156.4)	187.2 (127.4, 260.8)
Australasia	18.4 (14.8, 23.2)	89.0 (71.8, 111.5)	43.7 (29.1, 60.6)	200.6 (133.5, 279.6)	47.4 (39.0, 60.6)	131.0 (109.8, 174.5)	71.8 (48.6, 100.5)	199.4 (134.5, 277.5)
Caribbean	89.5 (54.0, 118.7)	244.5 (153.9, 314.9)	49.8 (33.9, 69.7)	166.6 (112.2, 233.5)	92.4 (69.9, 122.3)	203.2 (151.8, 270.9)	80.9 (54.5, 114.0)	161.9 (109.3, 227.1)

Central Asia	27.2 (20.9, 31.5)	34.3 (27.3, 40.6)	121.1 (82.2, 169.8)	200.9 (137.2, 278.8)	47.8 (36.9, 57.3)	52.5 (40.2, 62.7)	179.4 (120.8, 250.8)	193.5 (130.7, 270.9)
Central Europe	73.2 (58.8, 79.9)	66.2 (53.6, 72.2)	376.7 (255.8, 528.4)	282.6 (190.8, 397.9)	50.3 (41.5, 67.8)	41.7 (33.7, 61.7)	334.4 (228.5, 463.0)	233.0 (158.4, 326.0)
Central Latin America	134.4 (104.7, 150.0)	86.4 (68.5, 97.6)	191.5 (128.8, 268.2)	164.5 (111.0, 230.6)	271.7 (211.6, 323.8)	112.8 (88.5, 134.4)	427.0 (289.5, 599.9)	174.6 (118.2, 245.8)
Central Sub-Saharan Africa	75.5 (23.5, 139.4)	100.1 (39.6, 156.6)	90.3 (61.2, 126.9)	226.3 (154.0, 316.0)	84.8 (37.8, 127.4)	70.2 (30.0, 111.4)	211.3 (143.8, 296.5)	219.8 (149.7, 307.4)
East Asia	676.5 (499.5, 824.2)	59.7 (44.2, 72.5)	2457.2 (1642.4, 3459.2)	224.1 (149.6, 315.9)	528.3 (398.0, 612.2)	37.3 (27.5, 43.0)	4070.5 (2710.1, 5770.0)	211.8 (140.9, 300.5)
Eastern Europe	90.8 (63.2, 97.2)	43.2 (29.4, 46.2)	350.3 (237.7, 489.6)	142.8 (96.6, 200.7)	87.4 (73.0, 118.0)	44.8 (36.6, 59.5)	266.1 (183.3, 372.4)	110.1 (74.5, 155.0)
Eastern Sub-Saharan Africa	76.6 (55.1, 98.3)	41.3 (32.0, 49.2)	305.8 (206.1, 427.0)	231.9 (156.7, 322.2)	122.5 (89.8, 166.9)	38.4 (29.8, 49.1)	660.3 (441.4, 924.5)	225.8 (152.3, 313.3)
High-income Asia Pacific	71.9 (65.3, 94.8)	46.1 (41.4, 60.6)	580.1 (385.7, 816.9)	300.2 (200.0, 423.5)	80.3 (66.0, 99.8)	32.5 (28.9, 45.5)	653.4 (436.4, 911.9)	271.5 (180.6, 380.2)
High-income North America	306.4 (249.4, 398.3)	104.0 (84.0, 134.1)	688.2 (467.5, 949.9)	217.8 (147.6, 299.1)	830.6 (650.2, 972.7)	175.9 (142.1, 217.2)	822.1 (569.5, 1125.5)	170.1 (116.4, 233.0)
North Africa and Middle East	445.6 (267.0, 768.3)	108.1 (70.0, 174.6)	733.7 (486.7, 1039.9)	258.2 (173.0, 361.2)	536.5 (376.0, 693.2)	98.0 (68.6, 125.5)	1458.4 (964.3, 2053.2)	253.9 (170.5, 355.5)
Oceania	4.5 (3.4, 5.9)	89.7 (67.1, 119.7)	8.5 (5.7, 11.8)	169.0 (114.6, 233.4)	10.3 (7.3, 13.8)	93.1 (64.9, 126.4)	18.0 (12.1, 25.2)	160.8 (107.9, 223.0)
South Asia	468.0 (329.9, 640.8)	37.0 (27.7, 48.7)	2213.9 (1492.3, 3039.1)	255.4 (174.0, 352.3)	555.8 (435.8, 720.7)	33.8 (26.6, 43.5)	4375.1 (2979.8, 6047.8)	261.6 (178.5, 361.6)
Southeast Asia	265.7 (167.9, 346.2)	60.3 (40.1, 74.4)	523.6 (349.7, 732.3)	139.6 (93.1, 193.3)	302.3 (233.8, 362.1)	48.5 (37.5, 58.1)	847.6 (559.9, 1184.7)	118.8 (79.3, 165.9)

Southern Latin America	68.5 (37.8, 77.0)	143.4 (77.9, 161.1)	127.2 (85.6, 178.0)	266.3 (179.3, 374.0)	61.3 (52.7, 103.9)	90.3 (76.3, 153.8)	194.3 (131.9, 270.6)	258.9 (174.8, 362.0)
Southern Sub-Saharan Africa	83.9 (58.0, 97.1)	181.8 (132.6, 209.3)	139.2 (93.1, 195.9)	352.7 (237.9, 488.6)	129.0 (102.8, 157.2)	182.3 (144.4, 216.6)	244.4 (164.4, 342.0)	347.0 (235.3, 480.0)
Tropical Latin America	88.9 (77.9, 118.2)	64.9 (57.4, 87.8)	174.9 (119.1, 241.8)	141.2 (96.3, 195.0)	309.0 (194.9, 341.4)	138.0 (88.8, 154.0)	235.8 (161.7, 329.8)	98.8 (67.9, 138.4)
Western Europe	348.9 (278.7, 425.7)	86.5 (68.9, 104.4)	1444.9 (972.1, 2010.2)	314.9 (210.6, 437.8)	525.4 (411.8, 626.9)	94.6 (73.9, 117.2)	1888.1 (1283.0, 2639.4)	320.5 (216.7, 445.1)
Western Sub-Saharan Africa	101.8 (63.6, 132.5)	51.1 (33.3, 62.5)	347.7 (236.3, 485.6)	236.6 (161.3, 328.2)	195.3 (140.4, 262.7)	47.0 (35.4, 59.6)	849.2 (571.6, 1195.1)	244.7 (165.6, 340.6)

EMBID: endocrine, metabolic, blood and immune disorders. **YLLs:** years of life lost. **YLDs:** years lived with disability.

Table S3: The EMBID-related deaths, age-standardized death rate and DALYs, age-standardized DALY rates in 1990 and 2019, and percent change from 1990 to 2019.

	**1990**				**2019**				**1990-2019**		**1990-2019**	
	**Deaths**		**DALYs**		**Deaths**		**DALYs**		**Percent change in number (%)**		**Percent change in age-standardized rates (%)**	
	Deaths (95% UI)	Age-standardized death rate per 100,000 (95% UI)	DALYs (95% UI)	Age-standardized DALY rate per 100,000 (95% UI)	Deaths (95% UI)	Age-standardized death rate per 100,000 (95% UI)	DALYs (95% UI)	Age-standardized DALY rate per 100,000 (95% UI)	**Deaths**	**DALYs**	**Deaths**	**DALYs**
Global	77687.3 (65203.7, 90125.3)	1.8 (1.5, 2.1)	14612663.3 (10859985.4, 18945978.6)	300.1 (221.2, 391.8)	162039.2 (131144.7, 178615.4)	2.1 (1.7, 2.3)	22906564.1 (17001209.2, 29969465.0)	285.9 (213.0, 373.3)	108.6 (77.9, 129.1)	56.8 (48.5, 64.1)	16.1 (1.7, 24.9)	-4.7 (-8.1, -1.3)
**Countries**												
Afghanistan	311.4 (160.4, 492.8)	2.7 (1.6, 3.9)	43522.7 (28053.6, 61365.3)	365.7 (256.4, 493.0)	762.9 (472.7, 1057.8)	3.0 (1.9, 4.0)	115745 (83984.3, 150840.2)	342.8 (253.8, 448.3)	145.0 (42.1, 326.6)	165.9 (89.3, 256.1)	10.3 (-33.1, 67.0)	-6.2 (-25.8, 12.9)
Albania	90.7 (67.5, 115.4)	3.6 (2.3, 4.2)	12037.7 (9205.1, 16525.7)	389.5 (299.6, 511.7)	71.5 (52.2, 97.1)	2.1 (1.5, 3.1)	10159.7 (7345.3, 13957.9)	332.7 (238.5, 452.7)	-21.1 (-43.2, 12.5)	-15.6 (-29.4, -1.3)	-40.9 (-60.9, -1.2)	-14.6 (-25.1, -0.5)

Algeria	385.0 (274.7, 569.4)	2.1 (1.6, 2.8)	78395.4 (58320.4, 103651.4)	349.4 (258.3, 458.9)	783.4 (524.4, 993.8)	2.5 (1.7, 3.2)	133779.2 (96946.1, 176544.9)	330.4 (242.0, 432.2)	103.5 (11.3, 193.9)	70.6 (35.8, 97.7)	19.0 (-25.0, 63.6)	-5.4 (-19.1, 5.4)
American Samoa	3.1 (2.2, 4.3)	12.4 (9.1, 16.8)	187.4 (142.7, 244.7)	532.4 (410.0, 691.2)	7.4 (5.0, 9.3)	16.1 (10.9, 20.3)	319.8 (242.6, 407.0)	618.8 (469.5, 786.4)	139.6 (61.7, 252.2)	70.6 (28.8, 125.5)	30.1 (-12.1, 89.3)	16.2 (-12.0, 52.0)
Andorra	0.7 (0.5, 1.0)	1.5 (1.1, 2.0)	206.9 (144.4, 278.3)	365.3 (258.1, 490.8)	2 .0 (1.4, 2.6)	1.5 (1.1, 2.0)	386.2 (269.1, 531.0)	348.0 (245.6, 472.6)	172.9 (87.9, 284.0)	86.6 (71.0, 102.1)	-2.8 (-31.8, 36.0)	-4.7 (-11.3, 2.1)
Angola	191.5 (65.1, 338.5)	2.1 (0.9, 3.1)	29348.2 (18568.4, 43107.4)	317.9 (220.4, 422.7)	383.7 (163.5, 594.2)	2.0 (0.8, 3.5)	70618.5 (48433.1, 95451.9)	301.2 (207.8, 408.8)	100.4 (14.9, 256.1)	140.6 (74.4, 213.9)	-2.1 (-34.2, 39.6)	-5.3 (-20.2, 8.5)
Antigua and Barbuda	4.8 (3.2, 5.5)	8.5 (5.7, 9.7)	257.6 (196.0, 306.3)	454.7 (343.8, 541.6)	8.2 (6.6, 11.6)	9.3 (7.5, 13.1)	411.4 (328.5, 536.3)	454.3 (363.4, 595.0)	68.8 (29.5, 196.6)	59.7 (34.2, 123.6)	9.1 (-16.0, 90.8)	-0.1 (-17.2, 41.7)
Argentina	1781.5 (766.5, 2032.8)	6.1 (2.5, 7.1)	146631.4 (106825.3, 188365.2)	451.6 (327.9, 580.2)	1297.7 (1054.4, 2634.6)	2.6 (2.1, 5.1)	170438.4 (122760.4, 227684.7)	353.5 (255.8, 473.1)	-27.2 (-45.3, 170.8)	16.2 (0.4, 65.5)	-58.2 (-68.8, 62.6)	-21.7 (-32.3, 13.5)
Armenia	12.6 (11.0, 17.0)	0.4 (0.4, 0.5)	6411.4 (4476.9, 8860.5)	197.1 (137.2, 270.7)	33.6 (21.2, 40.6)	1.0 (0.6, 1.2)	7575.1 (5426.0, 10235.3)	219.5 (158.5, 292.2)	166.7 (74.0, 226.3)	18.2 (8.9, 27.9)	136.3 (59.0, 185.5)	11.4 (3.7, 20.1)
Australia	478.0 (387.7, 620.8)	2.6 (2.1, 3.4)	51673.3 (39244.9, 66294.5)	289.4 (220.1, 370.9)	1913.5 (1460.1, 2217.6)	4.8 (3.7, 5.7)	100955.8 (80030.6, 128006.4)	330.5 (261.0, 415.1)	300.3 (231.0, 344.1)	95.4 (82.6, 110.2)	81.9 (60.2, 98.7)	14.2 (7.7, 23.1)

Austria	356.6 (307.0, 520.9)	3.5 (3.0, 5.0)	54566.0 (40389.7, 71323.6)	596.5 (440.5, 778.5)	946.0 (679.3, 1068.8)	6.1 (4.3, 6.8)	77341.6 (58440.6, 98805.9)	637.0 (488.6, 815.2)	165.3 (57.0, 222.8)	41.7 (24.4, 55.4)	76.2 (1.5, 113.1)	6.8 (-5.7, 16.5)
Azerbaijan	112.5 (69.9, 143.1)	1.4 (1.0, 1.7)	21088.9 (15017.0, 27595.8)	295.8 (215.2, 386.4)	153.5 (103.9, 197.1)	1.9 (1.2, 2.4)	30267.4 (21812.0, 40147.3)	297.2 (210.2, 393.0)	36.4 (5.7, 76.4)	43.5 (23.9, 63.8)	29.1 (2.1, 64.7)	0.5 (-10.4, 15.6)
Bahamas	29.1 (19.6, 33.1)	15.8 (10.6, 18.0)	1719.4 (1238.9, 2018.7)	779.2 (570.9, 917.8)	47.9 (37.4, 69.8)	13.4 (10.5, 19.6)	2386.4 (1933.4, 3150.9)	645.0 (524.2, 860.3)	64.9 (25.2, 179.6)	38.8 (10.7, 105.0)	-15.2 (-35.4, 41.9)	-17.2 (-33.7, 23.0)
Bahrain	14.5 (11.8, 19.2)	6.5 (5.0, 7.8)	1949.7 (1508.3, 2505.9)	472.2 (371.2, 598.5)	56.2 (31.0, 74.8)	8.6 (4.4, 11.7)	5972.5 (4358.3, 7889.6)	464.0 (342.2, 594.1)	287.6 (104.1, 443.1)	206.3 (133.7, 257.4)	33.7 (-23.8, 81.3)	-1.7 (-21.9, 12.6)
Bangladesh	943.3 (614.2, 1394.9)	1.0 (0.7, 1.2)	225431.8 (162762.4, 297442.7)	249.6 (179.8, 328.4)	986.2 (767.9, 1342.9)	0.8 (0.6, 1.0)	322365.5 (232185.8, 434889.9)	216.7 (157.2, 290.7)	4.5 (-29.4, 73.6)	43.0 (18.9, 68.3)	-16.2 (-36.3, 16.2)	-13.2 (-21.6, -4.1)
Barbados	25.7 (17.7, 28.9)	9.3 (6.5, 10.4)	1246.4 (962.1, 1476.1)	492.4 (380.1, 581.9)	37.1 (28.8, 56.2)	8.8 (6.8, 13.4)	1556.0 (1230.9, 2076.8)	433.7 (339.3, 596.1)	44.4 (8.1, 146.5)	24.8 (2.9, 77.9)	-6.2 (-30.2, 61.1)	-11.9 (-29.1, 29.7)
Belarus	39.7 (34.2, 66.2)	0.4 (0.3, 0.6)	17434.8 (12243.3, 23798.8)	156.6 (110.7, 213.7)	136.9 (73.0, 180.8)	1.1 (0.6, 1.5)	20131.3 (14610.2, 26808.1)	184.5 (134.3, 243.7)	244.8 (36.6, 394.0)	15.5 (-0.8, 30.7)	209.2 (26.2, 345.9)	17.8 (0.1, 34.9)
Belgium	441.4 (314.0, 485.4)	3.2 (2.3, 3.6)	52471.7 (39017.0, 69590.7)	447.4 (334.3, 588.2)	677.9 (531.7, 806.7)	3.1 (2.5, 3.9)	68237.1 (50477.1, 90223.9)	460.1 (338.8, 604.0)	53.6 (37.0, 86.9)	30.0 (24.4, 36.9)	-2.6 (-12.1, 22.3)	2.8 (-1.9, 8.4)

Belize	11.3 (5.9, 13.5)	7.2 (4.5, 8.2)	905.9 (505.9, 1122.1)	483.6 (322.4, 588.5)	21.3 (17.3, 32.8)	6.9 (5.5, 10.8)	1465.6 (1205.4, 1887.6)	408.9 (335.2, 529.0)	88.8 (38.5, 337.0)	61.8 (25.4, 223.6)	-4.9 (-27.5, 92.0)	-15.5 (-30.9, 42.2)
Benin	57.7 (35.5, 74.6)	1.8 (1.1, 2.3)	10512.8 (7793.5, 13745.2)	275.5 (199.9, 365.4)	135.1 (88.7, 197.9)	1.8 (1.3, 2.5)	27038.7 (19522.3, 35866.6)	275.9 (198.6, 365.5)	134.4 (58.5, 240.4)	157.2 (124.1, 194.8)	-1.5 (-33.3, 49.8)	0.1 (-9.4, 11.1)
Bermuda	5.5 (3.5, 6.2)	9.5 (6.0, 10.9)	286.5 (217.0, 344.0)	474.4 (359.4, 566.6)	6.7 (5.2, 9.2)	5.7 (4.4, 8.2)	303.9 (233.2, 400.1)	332.1 (257.5, 443.8)	22.4 (-5.6, 101.8)	6.1 (-10.1, 39.2)	-40.5 (-54.5, 4.2)	-30.0 (-41.5, -2.5)
Bhutan	5.9 (2.4, 10.7)	1.2 (0.6, 1.7)	1450.7 (963.0, 1982.2)	279.4 (192.8, 374.2)	8.2 (5.6, 11.5)	1.4 (1.0, 2.0)	1903.4 (1382.2, 2525.6)	276.2 (202.6, 364.4)	39.7 (-19.5, 227.4)	31.2 (1.7, 69.5)	23.5 (-16.0, 100.8)	-1.2 (-14.5, 16.8)
Bolivia (Plurinational State of)	175.5 (116.1, 227.4)	3.6 (2.4, 4.4)	20263.4 (15131.1, 25719.2)	343.4 (263.9, 431.0)	244.2 (177.7, 357.3)	2.8 (2.1, 4.1)	28906.8 (21289.8, 38494.1)	268.5 (198.3, 356.0)	39.1 (-10.9, 140.9)	42.7 (7.7, 86.3)	-21.8 (-46.6, 28.1)	-21.8 (-35.2, -4.1)
Bosnia and Herzegovina	39.4 (28.5, 45.2)	1.0 (0.7, 1.1)	12977.1 (9118.2, 17772.5)	286.1 (201.6, 392.1)	40.9 (29.6, 53.0)	0.8 (0.6, 1.1)	11293.9 (7849.9, 15807.1)	274.8 (192.8, 378.4)	3.9 (-21.7, 35.4)	-13.0 (-20.3, -5.2)	-13.7 (-33.7, 11.6)	-4.0 (-9.8, 2.4)
Botswana	21.3 (10.5, 35.7)	2.9 (1.5, 4.9)	4093.4 (2890.3, 5612.8)	436.8 (305.1, 594.0)	74.1 (37.1, 113.2)	4.5 (2.3, 6.8)	10920.2 (7803.0, 14763.3)	544.2 (387.8, 736.0)	248.0 (117.5, 453.7)	166.8 (132.4, 206.7)	53.4 (-4.2, 143.2)	24.6 (9.4, 42.6)
Brazil	1849.0 (1661.8, 2596.2)	1.8 (1.6, 2.6)	256625.8 (200170.2, 325037.5)	206.2 (160.2, 261.0)	9936.8 (5996.7, 10923.3)	4.4 (2.7, 4.9)	531395.3 (401818.8, 638577.3)	237.6 (180.6, 284.6)	437.4 (192.9, 517.6)	107.1 (59.9, 138.7)	140.6 (29.2, 175.0)	15.2 (-11.5, 34.1)

Brunei Darussalam	10.0 (8.1, 13.3)	7.3 (5.9, 9.2)	1170.9 (920.0, 1496.2)	533.7 (420.2, 674.6)	20.9 (17.4, 26.4)	7.9 (6.2, 9.5)	2154.3 (1672.8, 2754.3)	524.2 (416.8, 658.2)	109.5 (74.0, 155.5)	84 (64.5, 104.2)	8.4 (-11.6, 31.9)	-1.8 (-10.9, 8.5)
Bulgaria	88.2 (75.8, 119.0)	1.0 (0.8, 1.3)	30219.7 (21409.4, 41374.3)	312.6 (224.7, 422.8)	91.8 (70.1, 126.6)	1.0 (0.7, 1.3)	25530.5 (18032.5, 34907.8)	295.3 (209.5, 399.1)	4.0 (-18.1, 30.3)	-15.5 (-20.7, -9.5)	-2.6 (-24.3, 24.7)	-5.5 (-11.3, 1.3)
Burkina Faso	76.8 (58.0, 99.0)	1.2 (0.9, 1.6)	19556.7 (14408.3, 25773.5)	255.8 (185.7, 344.4)	219.7 (161.7, 287.8)	1.6 (1.2, 2.0)	52878.5 (39006.1, 69422.5)	285.8 (207.0, 377.0)	186.1 (120.5, 271.2)	170.4 (142.5, 200.5)	27.8 (-1.3, 61.3)	11.8 (4.2, 20.1)
Burundi	45.0 (26.4, 59.9)	1.3 (0.8, 1.8)	10424.3 (7513.2, 13927.2)	254.9 (181.6, 342.1)	74.2 (42.7, 120.7)	1.2 (0.7, 1.9)	20343.1 (14430.9, 28113.9)	237.8 (167.4, 323.3)	65.1 (3.1, 160.8)	95.2 (68.4, 122.7)	-7.8 (-36.1, 36.1)	-6.7 (-14.8, 2.5)
Cabo Verde	1.5 (1.2, 2.0)	0.5 (0.4, 0.7)	727.7 (513.7, 991.1)	255.8 (178.6, 351.9)	3.5 (2.7, 4.9)	0.8 (0.6, 1.1)	1340.1 (930.9, 1851.0)	259.5 (181.9, 356.3)	133.4 (73.2, 202.5)	84.2 (67.6, 100.1)	56.4 (21.7, 95.4)	1.5 (-4.4, 7.5)
Cambodia	147.5 (92.1, 213.2)	2.2 (1.6, 2.7)	20040.5 (13622.8, 27175.2)	229.4 (168.1, 297.4)	227.4 (171.1, 288.5)	2.0 (1.5, 2.5)	28922.6 (21636.6, 37554.3)	191.7 (144.5, 247.7)	54.2 (-0.2, 169.0)	44.3 (9.0, 87.6)	-6.7 (-29.4, 36.3)	-16.4 (-28.3, -1.9)
Cameroon	155.7 (90.6, 210.0)	2.8 (1.6, 3.6)	23958.8 (17196.4, 31351.6)	302.8 (221.2, 396.9)	349.8 (216.2, 515.5)	2.2 (1.5, 3.2)	63632.8 (46188.0, 84326.0)	287.7 (205.8, 381.3)	124.6 (52.7, 224.5)	165.6 (133.0, 199.9)	-18.8 (-43.5, 23.0)	-5.0 (-14.7, 6.2)
Canada	965.0 (711.4, 1176.3)	3.2 (2.3, 3.9)	91942.4 (70314.8, 116819.8)	308.6 (237.7, 389.3)	2669.8 (2188.9, 3458.0)	4.2 (3.6, 5.7)	153392.3 (119412.6, 197567.9)	311.5 (245.4, 395.5)	176.7 (143.7, 235.8)	66.8 (54.3, 91.9)	32.0 (16.9, 62.1)	0.9 (-5.7, 17.1)

Central African Republic	50.9 (19.3, 77.4)	2.3 (1.0, 3.6)	7576.5 (4848.6, 10570.1)	311.2 (215.9, 412.2)	79.2 (34.1, 119.0)	2.3 (1.0, 3.7)	13172.7 (9037.9, 17871.5)	302.9 (211.5, 407.7)	55.7 (11.2, 126.4)	73.9 (41.0, 107.0)	-2.0 (-26.2, 30.1)	-2.7 (-14.1, 8.5)
Chad	52.2 (32.9, 70.3)	1.3 (0.8, 1.9)	12960.2 (9370.2, 17387.0)	267.3 (191.8, 360.9)	106.2 (77.1, 138.9)	1.1 (0.8, 1.5)	32121.4 (23137.0, 43056.1)	259.8 (186.5, 349.5)	103.6 (48.8, 177.5)	147.8 (124.0, 173.5)	-12.6 (-36.7, 26.0)	-2.8 (-9.6, 3.9)
Chile	194.9 (165.0, 249.6)	1.9 (1.6, 2.4)	35885 (26996.5, 46742.6)	303.3 (227.7, 396.6)	627.9 (534.1, 796.7)	2.9 (2.4, 3.7)	69760.7 (52953.5, 90251.1)	333.6 (253.4, 430.2)	222.1 (191.9, 257.2)	94.4 (85.7, 103.7)	54.3 (40.0, 71.7)	10.0 (4.9, 17.3)
China	12158.9 (9059.7, 14483.4)	1.4 (1.1, 1.7)	3028147.9 (2209673.9, 3997157.9)	284.4 (206.4, 378.3)	18598.1 (13028.5, 22121.2)	1.2 (0.9, 1.4)	4468851.9 (3130885.4, 6155682.6)	250.1 (178.6, 339.2)	53 (17.7, 88.8)	47.6 (34.5, 61.1)	-14.8 (-33.6, 3.4)	-12.0 (-18.4, -6.7)
Colombia	386.4 (295.3, 427.3)	1.7 (1.3, 1.9)	59362.9 (45714.0, 75802.0)	233.3 (176.1, 305.4)	1033.0 (771.3, 1404.7)	2.0 (1.5, 2.7)	122085.3 (91124.9, 161094.2)	244.7 (183.9, 321.6)	167.4 (104.6, 260.8)	105.7 (83.6, 131.3)	21.3 (-7.3, 58.9)	4.9 (-4.9, 17.6)
Comoros	4.2 (2.3, 5.8)	1.5 (0.8, 2.0)	1028.5 (729.3, 1396.2)	287.6 (205.1, 389.1)	8.5 (5.4, 11.5)	1.7 (1.1, 2.3)	1700.0 (1219.1, 2275.4)	274.3 (196.7, 366.5)	101.1 (41.9, 261.2)	65.3 (45.7, 95.3)	16.7 (-15.1, 90.7)	-4.6 (-13.2, 8.3)
Congo	47.7 (18.8, 71.7)	2.8 (1.3, 4.1)	7042.7 (4678.4, 9500.2)	349.3 (242.3, 464.3)	80.8 (35.4, 121.2)	2.5 (1.1, 3.9)	13530.4 (9559.6, 17999.3)	312.5 (222.7, 419.5)	69.3 (14.1, 150.1)	92.1 (52.0, 130.8)	-10.1 (-32.9, 19.5)	-10.5 (-22.0, 0.1)
Cook Islands	0.1 (0.0, 0.1)	0.4 (0.3, 0.6)	30.7 (21.2, 42.0)	188.1 (130.6, 256.2)	0.1 (0.1, 0.1)	0.5 (0.4, 0.6)	38.7 (26.6, 53.5)	185.7 (127.9, 253.4)	60.7 (14.2, 121.5)	26.0 (16.0, 36.7)	3.5 (-25.7, 48.1)	-1.3 (-7.3, 4.8)

Costa Rica	25.5 (21.5, 32.5)	1.1 (1.0, 1.5)	4985.5 (3719.8, 6559.9)	210.4 (153.8, 280.3)	93.0 (69.3, 118.4)	1.9 (1.4, 2.4)	11524.5 (8574.6, 15394.8)	233.5 (175.0, 311.4)	264.9 (181.8, 366.4)	131.2 (110.4, 154.6)	64.1 (27.3, 110.7)	11.0 (2.5, 23.0)
Croatia	51.7 (37.8, 58.0)	1.0 (0.7, 1.2)	20102.8 (14471.9, 27120.9)	368.8 (267.2, 492.6)	47.6 (35.3, 67.5)	0.8 (0.6, 1.1)	20537.4 (14332.3, 27743.8)	387.5 (273.0, 521.4)	-7.8 (-31.5, 35.0)	2.2 (-3.6, 8.6)	-22.0 (-41.8, 15.5)	5.1 (-1.2, 12.7)
Cuba	188.1 (131.7, 207.3)	1.9 (1.3, 2.1)	24501.6 (18630.5, 31470.2)	238.1 (180.1, 306.3)	282.6 (206.8, 513.9)	1.8 (1.4, 3.2)	31540.7 (23264.0, 42638.3)	220.3 (166.4, 287.1)	50.2 (5.6, 227.5)	28.7 (12.6, 63.3)	-4.4 (-31.4, 93.6)	-7.5 (-18.1, 17.3)
Cyprus	32.1 (22.1, 40.5)	4.6 (3.1, 5.7)	3036.8 (2301.9, 3920.0)	381.9 (291.0, 489.1)	65.4 (36.5, 83.1)	3.8 (2.2, 4.8)	5679.5 (4174.2, 7387.7)	341.8 (255.3, 443.6)	103.8 (42.8, 168.5)	87.0 (66.7, 104.0)	-16.8 (-39.2, 7.8)	-10.5 (-19.8, -2.4)
Czechia	142.6 (104.7, 157.2)	1.3 (0.9, 1.4)	36805.2 (26260.9, 49458.5)	334.7 (242.0, 448.7)	285.6 (145.2, 361.3)	1.6 (0.9, 2.1)	50521.2 (36030.5, 68519.8)	361.1 (262.3, 487.8)	100.3 (18.4, 148.8)	37.3 (22.9, 54.8)	25.8 (-13.7, 56.3)	7.9 (-2.0, 20.3)
Côte d'Ivoire	111.5 (68.5, 148.2)	1.8 (1.1, 2.3)	26174.7 (19113.6, 34185.1)	284.5 (205.1, 375.4)	223.6 (150.7, 305.3)	1.6 (1.1, 2.1)	56683 (40657.3, 75743.6)	275.9 (198.3, 370.0)	100.4 (38.9, 183.2)	116.6 (91.7, 139.1)	-10.5 (-38.9, 33.7)	-3.0 (-11.2, 5.2)
Democratic People's Republic of Korea	292.7 (213.2, 428.9)	1.6 (1.2, 2.2)	56901.2 (41414.7, 75565.9)	268.6 (195.8, 356.5)	308.1 (219.3, 429.1)	1.1 (0.8, 1.6)	64071.9 (45002.5, 88076.1)	215.2 (152.7, 293.0)	5.3 (-27.1, 57.1)	12.6 (-5.1, 28.9)	-26.7 (-46.5, 2.0)	-19.9 (-29.8, -10.4)
Democratic Republic of the Congo	773.1 (287.0, 1333.0)	2.2 (1.0, 3.2)	117245.5 (72713.3, 174484.5)	326.5 (225.0, 436.0)	955.2 (399.8, 1659.9)	1.9 (0.7, 3.5)	189854.5 (131428.0, 261862.9)	281.1 (195.0, 389.1)	23.6 (-33.9, 135.7)	61.9 (10.1, 118.7)	-16.4 (-45.3, 21.0)	-13.9 (-29.6, 0.7)

Denmark	177.3 (150.7, 229.7)	2.6 (2.1, 3.3)	27498.7 (20506.5, 36473.4)	458.7 (342.0, 604.5)	334.0 (275.3, 417.9)	3.3 (2.6, 4.1)	34749.1 (25906.8, 45269.8)	462.0 (341.6, 600.2)	88.4 (57.5, 113.1)	26.4 (20.4, 32.6)	27.0 (7.8, 43)	0.7 (-4.1, 5.8)
Djibouti	2.6 (1.5, 4.1)	1.1 (0.7, 1.7)	945.0 (675.1, 1276.6)	276.3 (195.2, 374.0)	10.2 (5.8, 16.3)	1.5 (0.9, 2.3)	2704 (1938.5, 3636.7)	268.4 (192.4, 358.6)	286.2 (178.3, 443.9)	186.1 (159.3, 214.0)	35.5 (1.1, 84.8)	-2.9 (-9.2, 5.2)
Dominica	6.4 (4.5, 7.6)	9.0 (6.2, 10.6)	338.2 (256.5, 411.6)	478.1 (364.1, 581.4)	7.3 (5.6, 10.6)	9.3 (7.0, 13.4)	344.4 (268.3, 457.6)	490.3 (374.5, 655.3)	14.3 (-17.4, 89.4)	1.8 (-20.2, 43.8)	3.2 (-26.5, 71.1)	2.6 (-20.7, 46.3)
Dominican Republic	392.3 (126.8, 575.8)	5.7 (2.1, 7.9)	36145.1 (15302.9, 51310.5)	460.5 (239.4, 626.1)	562.9 (268.5, 899.7)	5.9 (2.8, 9.4)	39408.8 (25176.7, 55135.5)	379.4 (243.7, 528.0)	43.5 (-4.6, 148.3)	9.0 (-21.7, 79.2)	3.8 (-29.3, 59.3)	-17.6 (-36.8, 12.0)
Ecuador	252.9 (164.3, 296.2)	3.6 (2.4, 4.2)	23971.3 (17936.1, 29431.7)	278.8 (209.6, 348.2)	460.6 (343.0, 595.5)	3.2 (2.4, 4.2)	44515.1 (34333.2, 56878.6)	273.2 (211.4, 349.0)	82.1 (38.9, 166.9)	85.7 (55.5, 127.9)	-11.2 (-31.3, 31.5)	-2.0 (-14.9, 15.6)
Egypt	1391.9 (547.6, 2376.5)	2.9 (1.3, 4.4)	209128.7 (126839.2, 306233.0)	382.0 (251.8, 522.9)	2248.6 (1162.6, 3670.3)	3.5 (1.8, 5.7)	318553.7 (225821.6, 421953.9)	356.6 (253.3, 471.5)	61.6 (-9.0, 165.4)	52.3 (10.7, 93.6)	20.5 (-27.3, 79.8)	-6.7 (-26.0, 9.9)
El Salvador	181.3 (51.8, 265.8)	3.3 (1.2, 4.5)	19280.0 (8091.7, 27039.1)	362.8 (187.3, 491.0)	77.0 (54.0, 123.8)	1.3 (0.9, 2.0)	12682.3 (9170.9, 17006.1)	213.9 (154.6, 289.0)	-57.6 (-77.4, 86.3)	-34.2 (-57.3, 45.8)	-61.8 (-78.7, 28.9)	-41.0 (-57.8, 3.4)
Equatorial Guinea	8.0 (3.1, 12.8)	2.2 (1.0, 3.3)	1243.1 (798.4, 1708.6)	318.2 (221.2, 420.5)	16.9 (6.6, 29.6)	2.5 (1.0, 4.4)	3287.7 (2275.7, 4530.1)	329.1 (227.1, 450.4)	111.1 (17.9, 265.6)	164.5 (96.6, 235.7)	16.1 (-26.2, 71.7)	3.4 (-11.6, 20.7)

Eritrea	14.5 (8.8, 22.4)	1.0 (0.6, 1.6)	5662.1 (4027.1, 7686.7)	265.5 (188.8, 358.8)	40.9 (21.6, 69.9)	1.3 (0.7, 2.1)	12877.2 (9030.5, 17728.5)	260.4 (183.9, 356.1)	182.1 (92.0, 322.2)	127.4 (108.3, 149.6)	31.9 (-9.4, 88.0)	-1.9 (-8.5, 5.9)
Estonia	16.3 (13.1, 19.8)	0.9 (0.8, 1.2)	3164.9 (2336.8, 4194.9)	189.2 (141.5, 251.7)	24.0 (17.1, 31.0)	1.2 (0.9, 1.5)	2968.6 (2201.9, 3974.6)	186.0 (137.7, 246.0)	47.3 (10.0, 91.8)	-6.2 (-13.5, 2.4)	22.9 (-5.5, 60.2)	-1.7 (-9.4, 7.4)
Eswatini	20.7 (11.5, 27.6)	4.8 (2.8, 6.4)	2704.0 (1939.7, 3570.7)	478.8 (348.1, 625.7)	43.5 (22.7, 68.4)	6.1 (3.3, 9.4)	4606.2 (3238.0, 6304.9)	515.8 (362.5, 706.5)	110.2 (36.7, 212.5)	70.3 (44.0, 104.8)	26.9 (-17.2, 84.1)	7.7 (-8.5, 29.2)
Ethiopia	475.2 (334.2, 625.9)	1.6 (1.2, 2.1)	110386.1 (80542.2, 144287.1)	286.2 (206.7, 381.3)	595.8 (478.7, 793.3)	1.2 (1.0, 1.5)	199148.2 (140912.0, 270720.9)	262.3 (185.9, 354.7)	25.4 (-13.6, 111.2)	80.4 (57.1, 102.5)	-24.7 (-43.9, 6.1)	-8.4 (-15.8, -0.9)
Fiji	10.8 (7.8, 14.3)	2.7 (2.0, 3.6)	1469.4 (1103.6, 1929.9)	249.1 (187.9, 326.6)	22.3 (16.1, 29.4)	3.2 (2.3, 4.1)	2291.5 (1734.0, 2932.0)	260.1 (199.3, 331.8)	105.7 (38.1, 201.0)	55.9 (36.0, 80.1)	17.5 (-20.1, 70.2)	4.4 (-8.7, 21.4)
Finland	61.1 (54.0, 81.3)	1.0 (0.9, 1.3)	17958.7 (12912.3, 23806.4)	300.2 (217.8, 397.0)	117.8 (96.5, 156.6)	1.3 (1.0, 1.5)	22657.5 (16495.1, 30083.2)	287.9 (212.0, 379.3)	92.9 (69.5, 121.7)	26.2 (21.5, 31.4)	21.1 (3.5, 38.6)	-4.1 (-7.9, 0.4)
France	2699.8 (2082.8, 3094.6)	3.5 (2.8, 4.1)	263079.0 (197091.6, 343116.4)	401.5 (302.0, 520.0)	4787.3 (3734.3, 5982.5)	3.6 (3.0, 4.7)	349660.5 (264235.0, 459022.1)	404.0 (304.4, 523.5)	77.3 (56.6, 103.7)	32.9 (26.1, 40.5)	4.6 (-5.5, 20.8)	0.6 (-4.3, 6.5)
Gabon	20.8 (8.4, 31.6)	2.6 (1.1, 4.0)	3273.1 (2201.8, 4412.1)	374.1 (256.7, 502.6)	40.6 (18.1, 59.8)	3.5 (1.6, 5.2)	5602 (3888.7, 7594.3)	372.9 (258.4, 505.7)	95.6 (44.0, 174.0)	71.2 (42.5, 97.6)	34.2 (3.2, 78.4)	-0.3 (-10.1, 11.7)

Gambia	7.6 (4.7, 10.7)	1.5 (0.9, 2.1)	2171.2 (1558.2, 2918.4)	279.5 (197.4, 377.3)	18.9 (13.8, 24.4)	1.6 (1.2, 2.1)	4991.1 (3638.7, 6691.4)	285.2 (208.0, 381.9)	148.7 (70.7, 276.8)	129.9 (106.6, 152.1)	8.8 (-28.7, 70.9)	2.0 (-6.3, 10.5)
Georgia	18.1 (15.5, 27.6)	0.3 (0.3, 0.5)	12362 (8595.6, 17129.4)	210.8 (146.3, 288.6)	74.6 (20.3, 95.3)	1.5 (0.5, 1.8)	11510.1 (8291.0, 15432.8)	258.3 (188.4, 342.0)	313.3 (-5.5, 468.0)	-6.9 (-22.0, 4.9)	343.5 (13.3, 501.6)	22.6 (3.2, 37.7)
Germany	3045.0 (2323.2, 3339.8)	2.7 (2.1, 3.0)	387209.6 (285013.1, 512044.8)	397.1 (292.9, 521.4)	5290.0 (3869.9, 6092.3)	3.2 (2.3, 3.7)	540834.0 (402387.8, 714296.7)	450.2 (335.0, 589.1)	73.7 (55.4, 90.0)	39.7 (30.1, 49.7)	19.5 (4.1, 29.5)	13.4 (5.7, 20.7)
Ghana	74.6 (52.3, 107.6)	1.0 (0.7, 1.3)	27701.1 (19678.1, 37812.4)	248.6 (176.9, 338.6)	237.5 (170.0, 342.1)	1.4 (1.0, 1.9)	68178.6 (48480.7, 92613.9)	269.2 (191.5, 362.8)	218.2 (139.2, 320.0)	146.1 (128.8, 164.2)	39.4 (3.7, 86.9)	8.3 (2.0, 15.3)
Greece	97.4 (84.7, 154.1)	0.8 (0.7, 1.2)	38686.6 (26514.1, 53963.0)	314.5 (215.8, 435.9)	305.2 (206.2, 347.8)	1.4 (1.0, 1.6)	47078.9 (33724.4, 64108.6)	329.4 (234.6, 442.2)	213.3 (64.2, 282.8)	21.7 (12.3, 30.3)	87.2 (-2.1, 126.9)	4.7 (-2.8, 11.3)
Greenland	1.7 (1.4, 2.5)	4.5 (3.7, 6.1)	166.4 (128.8, 217.2)	341.6 (266.4, 438.6)	3.5 (2.2, 4.5)	5.6 (3.5, 7.2)	227.3 (172.8, 288.1)	349.9 (268.9, 438.9)	101.8 (22.0, 192.4)	36.6 (7.7, 65.1)	23.0 (-20.0, 69.7)	2.4 (-16.6, 22.0)
Grenada	7.1 (4.2, 8.4)	9.0 (5.2, 10.5)	396.2 (273.8, 477.9)	499.1 (350.4, 601.6)	8.0 (6.7, 11.4)	8.2 (6.9, 11.5)	440.5 (359.9, 581.6)	427.5 (350.3, 562.2)	12.4 (-12.1, 119.6)	11.2 (-10.8, 77.5)	-8.4 (-28.3, 80.2)	-14.4 (-30.0, 36.9)
Guam	2.7 (2.2, 3.7)	3.6 (2.9, 4.8)	318.7 (240.6, 409.1)	281.6 (213.6, 360.9)	7.6 (5.6, 9.5)	4.1 (3.0, 5.2)	557.2 (427.2, 705.6)	307.4 (235.7, 387.3)	180.6 (89.7, 277.2)	74.8 (47.1, 105.2)	13.4 (-20.3, 49.9)	9.2 (-7.4, 27.2)

Guatemala	191.4 (124.5, 221.5)	3.3 (2.2, 3.8)	21186.2 (15794.8, 25830.5)	295.7 (230.2, 369.3)	390.3 (295.1, 525.8)	3.4 (2.6, 4.4)	38060.5 (29289.4, 49456.7)	268.2 (205.3, 348.9)	103.9 (52.7, 226.4)	79.6 (48.8, 147.5)	0.7 (-21.8, 38.1)	-9.3 (-20.7, 12.2)
Guinea	85.0 (51.1, 109.9)	1.8 (1.1, 2.3)	15002.6 (11002.8, 19521)	280.5 (203.8, 368.0)	119.8 (84.0, 156.2)	1.5 (1.1, 1.9)	26905.8 (19451.3, 35950.7)	270.1 (195.4, 358.0)	41.0 (2.4, 97.2)	79.3 (56.2, 103.0)	-16.6 (-38.7, 19.2)	-3.7 (-11.8, 5.0)
Guinea-Bissau	12.2 (7.7, 16.5)	2.1 (1.3, 2.7)	2262.1 (1656.9, 2973.2)	289.1 (211.0, 382.6)	15.5 (11.3, 20.2)	1.6 (1.2, 2.1)	3965.2 (2867.4, 5274.5)	276.8 (200.7, 368.4)	26.9 (-9.3, 88.0)	75.3 (52.4, 99.6)	-22.0 (-43.5, 12.5)	-4.3 (-13.0, 5.2)
Guyana	47.6 (29.6, 56.6)	9.2 (6.1, 10.9)	3502.6 (2508.1, 4195.9)	524.1 (383.7, 625.7)	57.1 (42.5, 80.7)	9.1 (6.8, 13.0)	3591.0 (2782.2, 4690.7)	499.4 (387.7, 648.1)	19.9 (-15.3, 115.1)	2.5 (-20.6, 56.8)	-2.0 (-29.2, 73.1)	-4.7 (-25.1, 42.3)
Haiti	372.3 (235.2, 550.0)	6.9 (5.2, 8.8)	32969.2 (20335.9, 50538.2)	484.4 (351.7, 656.1)	529.4 (377.8, 745.9)	5.7 (4.1, 8.3)	47136.3 (35157.8, 63965.4)	412.9 (311.8, 536.9)	42.2 (-10.2, 123.8)	43.0 (-3.5, 107.0)	-16.7 (-39.7, 10.6)	-14.8 (-35.4, 8.6)
Honduras	149.3 (90.9, 197.5)	3.3 (2.5, 4.0)	15837.0 (10305.0, 20767.7)	335.3 (253.7, 419.5)	259.6 (191.2, 345.8)	4.0 (2.9, 5.4)	24015.2 (18302.8, 31131.7)	296.4 (228.7, 379.9)	73.8 (18.6, 160.9)	51.6 (12.1, 103.4)	20.3 (-13.5, 56.8)	-11.6 (-26.5, 3.6)
Hungary	184.9 (162.3, 238.8)	1.6 (1.4, 2.1)	39450.8 (28857.2, 52318.7)	348.9 (257.8, 460.7)	327.3 (212.0, 408.9)	2.2 (1.5, 2.7)	41039.1 (30128.7, 54351.1)	343.5 (253.4, 454.6)	77.0 (4.4, 126.8)	4.0 (-4.9, 13.4)	33.8 (-12.9, 69.4)	-1.6 (-9.3, 7.2)
Iceland	3.7 (3.1, 4.6)	1.3 (1.1, 1.7)	848.4 (598.4, 1150.8)	325.5 (230.1, 442.3)	8.4 (6.2, 10.0)	1.6 (1.2, 1.9)	1413.3 (1011.9, 1909.8)	329.5 (236.5, 441.1)	125.6 (81.5, 164.2)	66.6 (54.6, 78.6)	18.2 (-3.4, 38.5)	1.2 (-4.7, 8.4)

India	5250.3 (3989.4, 6564.7)	0.8 (0.6, 1.0)	2160517.4 (1549292.1, 2846988.7)	300.8 (215.7, 403.1)	8609.6 (7064.0, 10642.1)	0.8 (0.6, 1.0)	4032994.1 (2845614.4, 5448197.5)	306.9 (217.6, 413.6)	64.0 (33.2, 113.5)	86.7 (74.4, 98)	-2.0 (-16.2, 18.5)	2.0 (-2.0, 5.8)
Indonesia	1210.0 (853.7, 1536.1)	0.8 (0.7, 1.0)	275430.9 (199384.4, 362314.8)	172.3 (124.9, 226.5)	1930.7 (1557.7, 2316.4)	1.0 (0.8, 1.2)	311791.6 (229589.8, 409230.1)	119.8 (90.5, 155.8)	59.6 (22.5, 116.8)	13.2 (0.0, 25.8)	18.4 (-0.3, 39.9)	-30.5 (-36.1, -23.3)
Iran (Islamic Republic of)	824.0 (650.0, 1262.2)	2.0 (1.6, 2.7)	180387.6 (135397.3, 240082.6)	345.4 (253.7, 456.3)	1617.9 (956.6, 1887.5)	2.4 (1.4, 2.8)	284591.1 (205634.8, 377449.6)	343.5 (251.7, 456.2)	96.4 (-9.5, 172.9)	57.8 (19.7, 80.4)	21.7 (-30.6, 63.7)	-0.5 (-18.0, 9.2)
Iraq	170.9 (116.2, 233.1)	1.1 (0.8, 1.5)	46772.4 (33647.4, 62389.0)	307.3 (219.8, 410.6)	240.2 (178.7, 301.0)	0.8 (0.6, 1.1)	99670.1 (69454.0, 136375.1)	272.7 (190.9, 371.8)	40.5 (0.1, 121.6)	113.1 (82.6, 142.3)	-24.4 (-42.8, 6.7)	-11.3 (-18.6, -3.9)
Ireland	92.3 (78.8, 121.8)	2.4 (2.1, 3.2)	13916.7 (10322.0, 18286.6)	378.1 (279.9, 495.4)	188.9 (154.5, 245.5)	2.8 (2.3, 3.7)	22861.4 (16655.1, 30411.5)	393.6 (291.8, 514.9)	104.8 (71.9, 130.5)	64.3 (54.3, 74.5)	15.6 (-4.3, 29.6)	4.1 (-1.7, 10.4)
Israel	189.9 (141.7, 210.5)	4.2 (3.0, 4.7)	19593.8 (14849.7, 25449.4)	407.5 (308.8, 530.2)	647.7 (377.3, 753.0)	5.4 (3.4, 6.2)	42294.3 (32285.8, 54600.7)	419.2 (319.7, 543.3)	241.2 (137.7, 288.6)	115.9 (99.6, 129.6)	27.7 (0.3, 43.4)	2.9 (-3.6, 9.1)
Italy	1387.4 (1261.1, 2009.9)	1.9 (1.7, 2.7)	313070.8 (223134.8, 424787.6)	465.1 (334.6, 622.5)	3779.6 (2440.2, 4185.8)	3.0 (1.9, 3.3)	381989.9 (280879.7, 505358.1)	462.1 (340.3, 604.1)	172.4 (47.1, 210.9)	22.0 (11.3, 30.8)	54.6 (-16.3, 73.8)	-0.6 (-8.5, 5.2)
Jamaica	49.6 (36.5, 56.2)	2.5 (1.9, 2.8)	5037.5 (3906.4, 6370.5)	246.2 (189.0, 313.4)	128.1 (97.1, 166.9)	4.2 (3.2, 5.5)	8042.7 (6235.3, 10267.7)	283.2 (219.5, 364.8)	158.4 (97.8, 240.1)	59.7 (39.2, 88.2)	68.8 (28.9, 124.2)	15.1 (0.4, 37.5)

Japan	1625.7 (1366.9, 2053.3)	1.2 (1.0, 1.5)	501807.9 (349086.4, 682701.9)	348.6 (245.9, 475.0)	3837.4 (2435.3, 4415.3)	1.1 (0.9, 1.4)	508602.7 (360310.7, 686952.6)	307.3 (214.6, 414.5)	136.0 (57.9, 165.6)	1.4 (-6.1, 8.6)	-6.3 (-17.9, 1.3)	-11.9 (-16.5, -7.0)
Jordan	60.4 (46.6, 75.6)	2.5 (2.0, 3.1)	11107.2 (8479.0, 14606.7)	347.7 (261.8, 455.5)	167.4 (128.4, 214.8)	2.2 (1.7, 2.9)	33632.5 (24823.0, 43955.9)	323.2 (239.1, 423.0)	177 (110.5, 269.3)	202.8 (166.5, 237.3)	-11.8 (-33.6, 12.9)	-7.1 (-14.7, 1.3)
Kazakhstan	81.2 (69.4, 133.8)	0.6 (0.5, 0.9)	36097.3 (25661.5, 48565.1)	235.3 (167.1, 318.3)	291.5 (188.4, 345.5)	1.7 (1.1, 2.0)	50543.3 (37452.6, 66540.5)	266.4 (197.7, 349.1)	259.1 (72.3, 356.9)	40.0 (26.6, 52.9)	205.6 (44.9, 285.5)	13.2 (3.1, 23.9)
Kenya	86.4 (63.6, 112.8)	0.8 (0.6, 1.1)	40567.4 (28457.4, 54977.8)	280.6 (194.2, 382.6)	260.9 (186.2, 368.7)	1.1 (0.8, 1.6)	98347.9 (68895.6, 134425.7)	268.7 (188.0, 363.8)	201.9 (133.6, 284.5)	142.4 (132.1, 151.8)	44.5 (19.8, 80.9)	-4.2 (-7.2, 0.0)
Kiribati	2.7 (1.9, 3.6)	6.9 (4.9, 9.8)	217.8 (163.4, 279.9)	383.7 (294.8, 481.8)	4.7 (3.1, 6.4)	7.1 (4.9, 10.2)	357.3 (264.4, 450.0)	371.2 (277.8, 463.8)	70.9 (30.0, 123.5)	64.0 (40.1, 90.0)	2.9 (-20.7, 34.0)	-3.3 (-16.6, 10.9)
Kuwait	17.0 (12.7, 20.0)	1.5 (1.1, 1.7)	5165.2 (3724.0, 6944.7)	344.7 (248.6, 459.5)	45.4 (36.0, 61.3)	1.7 (1.3, 2.2)	14478.6 (10185.4, 19771.9)	342.2 (248.1, 458.1)	167.4 (113.4, 254.7)	180.3 (150.8, 208.5)	14.1 (-6.4, 44.3)	-0.7 (-6.6, 6.6)
Kyrgyzstan	19.5 (15.9, 24.4)	0.5 (0.4, 0.6)	8540.3 (6137.6, 11442.6)	217.8 (154.4, 295.1)	39.4 (31.0, 48.1)	0.7 (0.5, 0.8)	13109.2 (9455.0, 17695.3)	213.9 (153.4, 288.2)	102.2 (69.6, 137.6)	53.5 (43.6, 63.9)	47.7 (23.5, 71.0)	-1.8 (-7.5, 4.1)
Lao People's Democratic Republic	76.6 (39.3, 122.6)	2.5 (1.6, 3.3)	8949.8 (5473.6, 13107.6)	238.5 (163.3, 318.7)	92.5 (66.5, 122.4)	2.0 (1.5, 2.6)	12158.2 (8994.5, 15880.0)	193.2 (144.1, 250.4)	20.8 (-24.6, 118.6)	35.8 (-2.7, 90.4)	-17.9 (-40.2, 21.5)	-19.0 (-34.2, -1.5)

Latvia	19.2 (15.6, 24.5)	0.6 (0.5, 0.8)	5255.1 (3797.4, 7056.3)	181.0 (131.1, 242.6)	33.7 (20.4, 42.9)	1.1 (0.7, 1.4)	5509.9 (3997.4, 7360.9)	225.3 (165.0, 299.4)	75.8 (9.6, 124.0)	4.8 (-3.3, 12.8)	73.7 (17.6, 120.8)	24.5 (15.3, 33.5)
Lebanon	50.9 (37.6, 68.7)	1.9 (1.5, 2.7)	10046.2 (7338.5, 13194.7)	325.6 (236.2, 432.3)	115.5 (75.2, 164.6)	2.3 (1.5, 3.2)	16532.3 (11958.1, 21875.9)	317.1 (229.4, 417.9)	127 (44.4, 248.7)	64.6 (42.5, 84.9)	17.5 (-26.8, 80.0)	-2.6 (-13.4, 8.1)
Lesotho	34.9 (19.5, 57.4)	3.0 (1.7, 4.9)	5603.1 (4005.3, 7645.0)	397.9 (287.4, 545.4)	81.7 (42.4, 119.9)	5.9 (3.0, 8.5)	8331.3 (5905.4, 11010.1)	481.0 (342.0, 633.7)	134.2 (41.1, 258.2)	48.7 (25.1, 77.4)	97.7 (21.1, 198.2)	20.9 (2.5, 45.1)
Liberia	34.3 (19.6, 49.3)	2.3 (1.3, 3.0)	5162.7 (3641.2, 6795.3)	295.9 (214.1, 386.6)	46.6 (30.1, 66.3)	1.8 (1.2, 2.6)	9516.5 (6819.9, 12695.3)	259.0 (187.0, 342.7)	35.9 (-3.7, 106.7)	84.3 (50.8, 118.5)	-19.4 (-44.5, 22.5)	-12.5 (-22.1, -1.9)
Libya	70.1 (44.4, 101.5)	2.2 (1.4, 3.1)	13335.3 (9749.8, 17682.3)	355.4 (256.2, 471.4)	144.7 (94.6, 206.9)	2.9 (1.9, 4.1)	21387.4 (15430.5, 28238.3)	336.6 (247.5, 441.7)	106.5 (25.5, 206.2)	60.4 (32.1, 86.7)	34.0 (-14.5, 95.2)	-5.3 (-16.7, 7.4)
Lithuania	21.3 (18.9, 31.0)	0.6 (0.5, 0.8)	10638.8 (7427.1, 14455.8)	267.5 (188.1, 363.3)	34.3 (25.7, 42.9)	0.9 (0.6, 1.1)	9895.4 (6994.7, 13398.1)	278.9 (200.0, 373.6)	61.2 (14.3, 101.0)	-7 (-12.6, -0.5)	55.6 (13.4, 91.1)	4.3 (-1.4, 11.0)
Luxembourg	13.1 (11.0, 16.1)	2.7 (2.3, 3.4)	1639.5 (1219.9, 2150.2)	366.0 (274.8, 475.1)	27.0 (20.0, 33.3)	2.9 (2.2, 3.6)	2934.4 (2167.5, 3825.2)	372.8 (276.1, 482.4)	105.9 (55.5, 147.5)	79.0 (67.9, 90.3)	4.1 (-19.4, 26.1)	1.9 (-5.1, 8.9)
Madagascar	78.1 (47.4, 105.4)	1.0 (0.6, 1.4)	23390.0 (16919.0, 31118.1)	260.0 (185.0, 350.5)	131.9 (75.1, 198.0)	1.0 (0.6, 1.5)	47805.3 (33306.4, 65233.5)	244.1 (172.1, 332.0)	68.8 (22.6, 131.2)	104.4 (85.5, 122.6)	-2.1 (-26.4, 28.8)	-6.1 (-12.1, 0.4)

Malawi	62.5 (44.3, 87.9)	1.0 (0.8, 1.3)	18943.9 (13766.3, 25378.6)	259.7 (185.3, 348.3)	98.7 (74.4, 130.1)	1.1 (0.8, 1.4)	33381.9 (23607.4, 45501.3)	252.2 (180.6, 344.1)	58.1 (5.6, 140.9)	76.2 (54.6, 98.1)	8.2 (-18.3, 44.1)	-2.9 (-10.0, 4.2)
Malaysia	165.1 (140.5, 207.2)	1.5 (1.3, 1.9)	28977.7 (21267.1, 37878.4)	199.8 (146.9, 262.1)	524.5 (365.7, 686.1)	2.1 (1.5, 2.7)	60045.9 (44466.6, 78474.5)	194.2 (144.7, 251.6)	217.8 (132.2, 329.7)	107.2 (87.0, 131.1)	36.3 (-2.8, 90.6)	-2.8 (-12.0, 9.0)
Maldives	10.6 (6.7, 15.3)	7.2 (5.3, 9.7)	913.5 (598.8, 1263.8)	420.2 (309.9, 542.2)	21.5 (17.3, 27.2)	6.7 (5.4, 8.6)	1432.0 (1149.1, 1786.2)	322.7 (258.5, 401.7)	102.5 (39.7, 229.4)	56.8 (9.6, 135.4)	-6.7 (-28.3, 27.3)	-23.2 (-39.2, -0.2)
Mali	98.9 (60.7, 133.2)	1.6 (1.0, 2.2)	21279.7 (15466.2, 27875.9)	287.4 (204.0, 380.9)	200.4 (138.3, 278.8)	1.4 (1.0, 1.9)	52485.7 (38432.6, 70154.7)	290.8 (208.8, 391.1)	102.7 (47.7, 188.3)	146.6 (116.7, 178.3)	-12.7 (-34.0, 26.1)	1.2 (-7.0, 9.9)
Malta	5.1 (4.1, 6.1)	1.3 (1.0, 1.6)	1179.1 (831.3, 1598.3)	293.1 (208.8, 394.7)	11.8 (9.2, 14.5)	1.5 (1.2, 1.9)	1998.4 (1433.7, 2694.6)	317.2 (229.9, 422.1)	131.9 (98.6, 172.5)	69.5 (57.7, 81.4)	16.5 (-1.0, 37.9)	8.2 (2.0, 15.3)
Marshall Islands	0.7 (0.5, 1.1)	3.9 (2.5, 5.8)	83.6 (62.0, 110.2)	278.0 (205.5, 367.2)	1.5 (1.0, 2.1)	4.1 (2.8, 5.7)	138.8 (103.9, 179.1)	280.3 (210.9, 361.9)	98.6 (46.7, 177.2)	66.1 (45.6, 91.7)	4.8 (-22.7, 46.8)	0.8 (-12.2, 17.6)
Mauritania	30.1 (18.7, 39.3)	2.6 (1.5, 3.4)	5139.9 (3766.8, 6729.7)	313.4 (228.4, 411.3)	39.1 (27.5, 55.1)	1.7 (1.2, 2.3)	8799.3 (6404.1, 11864.0)	276.3 (199.5, 368.4)	29.7 (-7.7, 102.6)	71.2 (51.3, 91.2)	-34.2 (-52.5, 5.6)	-11.8 (-21.0, -2.3)
Mauritius	19.0 (10.4, 21.6)	2.6 (1.4, 2.9)	2076.7 (1564.3, 2665.2)	215.9 (161.5, 276.0)	26.3 (19.9, 45.5)	1.7 (1.3, 3.0)	2814.5 (2047.9, 3829.5)	184.6 (136.5, 249.8)	38.4 (-2.6, 252.3)	35.5 (17.4, 80.9)	-32.4 (-52.2, 72.7)	-14.5 (-25.5, 16.6)

Mexico	1471.1 (1267.1, 1896.1)	2.9 (2.4, 3.5)	160289.6 (126717.7, 204047.3)	247.6 (195.3, 312.4)	5945.1 (3722.0, 7027.1)	5.2 (3.3, 6.1)	405058.1 (316117.0, 501320.2)	333.1 (261.0, 410.8)	304.1 (163.4, 384.1)	152.7 (108.6, 179.4)	79.0 (30.3, 110.0)	34.5 (18.7, 46.9)
Micronesia (Federated States of)	2.9 (2.1, 3.7)	5.7 (4.0, 7.7)	240.5 (185.9, 299.9)	331.2 (256.0, 417.8)	3.9 (2.4, 5.3)	5.8 (3.8, 7.7)	285.7 (207.4, 365.5)	320.1 (234.8, 405.7)	35.4 (-12.9, 94.1)	18.8 (-4.7, 40.5)	1.4 (-32.7, 46.8)	-3.4 (-23.2, 16.2)
Monaco	0.6 (0.4, 0.9)	1.0 (0.8, 1.5)	161.8 (110.4, 224.1)	381.6 (262.5, 526.6)	1.2 (0.8, 1.5)	1.4 (0.9, 1.8)	210.7 (145.0, 290.3)	379.9 (263.3, 519.0)	109.1 (17.3, 218.6)	30.2 (20.3, 39.4)	35.1 (-20.3, 100.4)	-0.4 (-7.3, 6.2)
Mongolia	55.0 (34.7, 72.8)	2.8 (1.8, 3.5)	6863.9 (5020.2, 8769.4)	336.2 (253.0, 424.2)	58.6 (43.4, 79.1)	2.0 (1.5, 2.6)	9054.8 (6795.6, 11771.6)	269.8 (202.7, 355.0)	6.5 (-29.1, 69.3)	31.9 (1.4, 70.1)	-29.5 (-49.3, 5.9)	-19.7 (-33.5, -2.1)
Montenegro	3.4 (2.8, 4.2)	0.6 (0.5, 0.7)	1883.1 (1313.9, 2597.3)	299.2 (211.3, 411.2)	4.3 (3.3, 5.4)	0.5 (0.4, 0.7)	1982.2 (1353.3, 2739.3)	271.0 (186.1, 373.6)	27.7 (2.3, 60.4)	5.3 (-2.2, 13.2)	-5.3 (-22.8, 19.5)	-9.4 (-14.8, -3.6)
Morocco	383.5 (219.5, 556.9)	1.9 (1.1, 2.7)	77119.5 (55187.4, 102201.0)	333.0 (238.8, 442.4)	801.5 (522.6, 1058.8)	2.9 (1.9, 3.7)	117328.3 (86177.9, 154688.9)	337.1 (250.6, 439.6)	109.0 (21.4, 212.9)	52.1 (23.0, 78.0)	52.8 (-2.0, 108.5)	1.2 (-13.8, 15.0)
Mozambique	108.7 (76.1, 179.2)	1.3 (1.0, 2.1)	27512.3 (20143.6, 36642.7)	266.5 (191.6, 357.8)	243.3 (162.7, 404.5)	1.7 (1.1, 2.7)	56505.8 (39997.7, 76402.8)	267.1 (191.2, 361.3)	123.8 (57.8, 221.0)	105.4 (79.6, 137.3)	26.0 (-6.6, 68.0)	0.2 (-6.8, 8.4)
Myanmar	690.7 (420.9, 1132.3)	2.3 (1.6, 3.1)	87360.3 (58681.7, 127591.1)	235.9 (166.7, 316.9)	934.3 (710.5, 1224.3)	2.2 (1.6, 2.7)	114153.9 (84068.2, 149589.8)	213.9 (158.8, 278.0)	35.3 (-16.0, 118.7)	30.7 (-4.6, 67.5)	-5.0 (-31.0, 31.5)	-9.3 (-26.6, 7.6)

Namibia	23.9 (11.7, 37.2)	2.8 (1.4, 4.4)	4448.8 (3100.8, 6097.7)	418.9 (293.2, 569.9)	51.0 (24.6, 89.5)	3.2 (1.6, 5.4)	8196.4 (5721.3, 11219.5)	420.0 (294.4, 570.2)	112.8 (47.0, 201.5)	84.2 (65.6, 109.2)	13.2 (-18.4, 55.0)	0.3 (-8.5, 12.4)
Nauru	0.2 (0.2, 0.3)	4.7 (3.3, 6.1)	24.9 (18.8, 31.7)	324.7 (251.1, 412.5)	0.3 (0.2, 0.4)	4.7 (3.2, 6.1)	25.5 (19.1, 32.9)	313.1 (236.4, 399.0)	6.5 (-17.5, 36.2)	2.4 (-9.4, 15.6)	-0.3 (-22.0, 29.2)	-3.6 (-14.3, 9.0)
Nepal	138.9 (86.8, 204.5)	0.8 (0.6, 1.0)	35246.9 (25512.5, 46702.8)	215.0 (154.2, 282.8)	265.2 (187.0, 354.8)	1.2 (0.8, 1.6)	54253.3 (39907.9, 71589.0)	199.7 (147.0, 262.1)	91.0 (34.1, 166.7)	53.9 (31.4, 74.8)	49.4 (13.3, 91.1)	-7.1 (-14.7, 2.0)
Netherlands	554.3 (385.1, 606.8)	3.1 (2.1, 3.4)	66897.0 (49610.6, 87702.2)	409.5 (305.6, 536.7)	682.9 (577.6, 1028.3)	2.4 (2.1, 3.5)	84917.3 (61293.7, 114282.6)	379.1 (278.6, 502.9)	23.2 (3.1, 103.6)	26.9 (16.9, 42.5)	-23.8 (-35.5, 24.9)	-7.4 (-14.0, 4.5)
New Zealand	105.6 (84.7, 127.9)	2.9 (2.3, 3.4)	10362.9 (8058.5, 13095.7)	290.6 (226.5, 368.8)	284.8 (233.6, 378.3)	4.2 (3.4, 5.4)	18180.7 (14378.0, 22468.7)	329.4 (262.6, 407.7)	169.7 (144.9, 209.4)	75.4 (65.8, 88.9)	45.5 (33.8, 63.6)	13.4 (7.5, 22.3)
Nicaragua	46.3 (33.5, 56.6)	1.5 (1.2, 1.8)	6959.9 (5281.0, 8917.3)	233.1 (175.6, 307.1)	81.8 (64.3, 105.5)	1.8 (1.4, 2.4)	11563.5 (8671.9, 15288.9)	214.6 (160.1, 283.5)	77.0 (36.4, 139.1)	66.1 (42.6, 94.9)	18.3 (-6.7, 50.3)	-7.9 (-15.4, -0.3)
Niger	77.5 (46.3, 106.4)	1.5 (1.0, 2.1)	17918.1 (12950.6, 23563.5)	277.8 (202.0, 373.8)	161.5 (107.4, 231.8)	1.2 (0.9, 1.8)	45625.7 (33483.7, 60707.1)	260.8 (188.2, 354.0)	108.3 (44.7, 216.3)	154.6 (121.2, 190.7)	-20.6 (-42.1, 14.6)	-6.1 (-13.3, 1.5)
Nigeria	872.6 (544.6, 1115.2)	1.5 (0.9, 1.9)	223579.9 (162959.4, 294967.3)	298.1 (212.2, 399.3)	1600.6 (1148.4, 2148.7)	1.3 (1.0, 1.7)	520139.2 (375562.1, 697754.9)	309.0 (222.0, 415.0)	83.4 (41.6, 151.3)	132.6 (117.6, 148.5)	-9.4 (-32.8, 31.0)	3.7 (-1.3, 8.9)

Niue	0.1 (0.1, 0.1)	3.8 (2.6, 5.1)	6.2 (4.6, 8.0)	286.7 (216.3, 373.4)	0.1 (0.1, 0.1)	3.8 (2.6, 5.1)	5.2 (3.9, 6.5)	272.4 (207.0, 345.1)	-9.0 (-37.0, 31.4)	-16.0 (-28.6, 0.0)	0.9 (-29.8, 47.2)	-5.0 (-19.2, 13.6)
North Macedonia	19.5 (11.9, 23.6)	1.1 (0.7, 1.3)	6346.7 (4545.0, 8542.3)	319.1 (228.7, 427.3)	14.4 (10.9, 20.9)	0.6 (0.5, 0.8)	6655.0 (4621.9, 9231.6)	265.7 (185.5, 368.3)	-26.0 (-48.8, 33.3)	4.9 (-5.3, 16.9)	-45.0 (-61.3, -3.7)	-16.7 (-25.1, -6.8)
Northern Mariana Islands	0.9 (0.6, 1.5)	4.5 (3.2, 6.6)	106.4 (78.9, 143.1)	292.3 (220.6, 386.5)	2.6 (1.9, 3.2)	5.7 (4.3, 7.1)	168.7 (129.5, 216.9)	318.7 (247.3, 403.8)	170.0 (68.7, 307.5)	58.5 (26.6, 94.6)	27.5 (-15.6, 85.9)	9.0 (-10.9, 30.3)
Norway	81.1 (71.4, 128.7)	1.4 (1.3, 2.1)	22013.3 (15616.7, 29618.1)	447.5 (317.0, 601.2)	189.2 (155.2, 240.4)	2.2 (1.8, 2.8)	32351.8 (23154.7, 43578.0)	482.3 (345.7, 645.0)	133.4 (60.3, 158.7)	47.0 (40.7, 52.8)	51.1 (9.4, 65.5)	7.8 (3.7, 11.4)
Oman	6.7 (4.8, 8.8)	0.6 (0.4, 0.8)	4683.4 (3268.1, 6516.1)	295.8 (203.3, 408.3)	14.0 (10.6, 16.6)	0.7 (0.6, 0.9)	10647.6 (7189.2, 14831.9)	275.8 (192.0, 378.0)	107.9 (60.6, 169.7)	127.3 (106.5, 151.9)	21.1 (-16.7, 70.5)	-6.8 (-11.8, -1.7)
Pakistan	942.7 (483.6, 1700.4)	0.9 (0.5, 1.5)	259224.9 (179671.1, 350797.6)	274.2 (194.0, 369.2)	2059.4 (1027.1, 3575.5)	1.2 (0.6, 2.0)	519377.4 (366510.7, 687067.7)	274.8 (194.4, 365.3)	118.5 (69.4, 189.5)	100.4 (81.1, 119.8)	30.3 (5.0, 61.7)	0.2 (-5.2, 7.5)
Palau	0.3 (0.2, 0.4)	3.1 (2.3, 4.2)	34.8 (26.0, 45.1)	266.7 (201.8, 348.0)	0.7 (0.5, 1.0)	3.7 (2.5, 5.3)	58.6 (43.0, 76.2)	265.4 (197.8, 341.5)	125.3 (48.1, 220.5)	68.5 (42.5, 99.4)	19.1 (-19.7, 65.9)	-0.5 (-15.4, 17.3)
Palestine	56.2 (33.2, 81.0)	2.7 (2.1, 3.5)	7806.0 (5216.1, 10532.9)	361.3 (265.3, 471.6)	57.2 (45.1, 75.7)	1.9 (1.5, 2.6)	12739.7 (9330.2, 17080.4)	297.2 (217.3, 395.6)	1.9 (-33.7, 77.0)	63.2 (20.5, 122.8)	-29.4 (-47.0, -5.1)	-17.7 (-29.8, -5.0)

Panama	42.8 (24.6, 49.1)	2.2 (1.3, 2.4)	5518.9 (4058.3, 7039.1)	273.0 (204.0, 351.4)	79.5 (57.7, 124.3)	1.9 (1.4, 3.0)	10633.1 (7950.2, 14083.1)	256.4 (191.7, 339.1)	85.7 (27.4, 316.1)	92.7 (65.0, 151.9)	-12.2 (-39.0, 78.7)	-6.1 (-17.9, 20.0)
Papua New Guinea	54.8 (38.1, 76.7)	2.5 (1.5, 3.7)	8020.2 (6046.3, 10368.3)	246.3 (183.5, 326.3)	145.1 (94.0, 220.9)	2.5 (1.5, 3.9)	19774.1 (14658.2, 26121.5)	239.4 (177.2, 316.7)	164.8 (96.7, 253.4)	146.6 (120.0, 179.9)	0.8 (-25.7, 34.6)	-2.8 (-12.0, 8.3)
Paraguay	60.9 (45.7, 75.1)	1.8 (1.5, 2.3)	7159.4 (5145.4, 9270.5)	189.7 (146.4, 242.5)	205.5 (114.6, 287.5)	3.6 (2.0, 5.0)	13397.5 (9582.7, 17188.1)	214.2 (152.3, 275.2)	237.3 (119.5, 376)	87.1 (53.6, 124.7)	106.1 (9.0, 211.4)	12.9 (-6.4, 38.1)
Peru	827.0 (359.9, 1118.6)	4.6 (2.1, 6.0)	84089.0 (49739.7, 111487.5)	406.6 (260.9, 529.3)	422.4 (227.8, 955.5)	1.3 (0.7, 2.9)	76830.7 (52026.4, 106180.3)	231.3 (157.3, 321.1)	-48.9 (-77.6, 108.4)	-8.6 (-37.9, 66.5)	-72.0 (-87.4, 7.3)	-43.1 (-58.7, -8.4)
Philippines	935.1 (738.0, 1091.7)	2.8 (1.8, 3.3)	122858.7 (93780.9, 156848.9)	245.9 (187.7, 312.9)	1835.4 (1387.3, 2210.5)	2.5 (1.7, 3.0)	254126.4 (189073.8, 333831.4)	253.6 (190.6, 332.4)	96.3 (62.4, 132.4)	106.8 (85.6, 127.7)	-9.9 (-25.2, 12.0)	3.1 (-3.3, 8.9)
Poland	661.3 (442.3, 710.7)	1.7 (1.1, 1.9)	146818.6 (106275.4, 196188.9)	371.0 (270.7, 493.8)	490.7 (384.5, 851.3)	0.9 (0.7, 1.7)	77088.2 (55832.4, 102948.0)	171.0 (122.9, 230.4)	-25.8 (-43.6, 56.4)	-47.5 (-52.9, -36.6)	-46.9 (-60.3, 19.0)	-53.9 (-59.0, -41.3)
Portugal	164.7 (145.4, 255.3)	1.5 (1.3, 2.2)	34833.8 (25095.7, 46921.0)	310.1 (225.6, 409.7)	714.6 (360.5, 826.7)	3.2 (1.7, 3.6)	43151.2 (32439.1, 55540.8)	292.4 (219.7, 372.2)	333.8 (58.7, 433.4)	23.9 (2.5, 38.9)	113.0 (-13.7, 157.4)	-5.7 (-20.7, 4.2)
Puerto Rico	379.6 (207.0, 429.3)	11.1 (6.0, 12.5)	18887.8 (12754.2, 22459.3)	533.3 (362.1, 633.4)	556.4 (416.5, 719.3)	8.4 (6.4, 11.2)	20081 (15709.5, 25162.7)	421.7 (331.7, 543.3)	46.6 (8.7, 154.7)	6.3 (-13.9, 50.2)	-23.9 (-44.5, 39.7)	-20.9 (-36.9, 20.6)

Qatar	8.1 (6.0, 10.9)	4.8 (3.4, 6.5)	1468.2 (1093.1, 1935.7)	419.1 (316.2, 545.2)	29.7 (21.0, 42.2)	5.6 (3.0, 8.4)	7939.5 (5523.6, 10951.6)	363.4 (266.8, 475.8)	265.2 (156.1, 439.1)	440.8 (357.4, 522.0)	15.7 (-26.6, 67.8)	-13.3 (-24.0, -1.6)
Republic of Korea	488.4 (400.5, 634.2)	1.7 (1.4, 2.1)	139198.1 (99894.9, 188363.7)	335.2 (241.7, 445.5)	665.8 (453.5, 817.6)	0.9 (0.6, 1.1)	201359.3 (137332.2, 279194.4)	294.5 (202.4, 406.4)	36.3 (-12.0, 80.6)	44.7 (28.2, 61.5)	-47.7 (-61.7, -33.1)	-12.1 (-20.2, -4.7)
Republic of Moldova	26.3 (23.2, 37.0)	0.6 (0.5, 0.9)	8034.8 (5896.8, 10774.4)	179.9 (131.7, 241.0)	43.0 (34.9, 59.5)	0.9 (0.8, 1.3)	7443.1 (5448.2, 9861.1)	178.2 (131.1, 235.3)	63.5 (34.2, 93.2)	-7.4 (-14.5, 0.2)	50.8 (25.5, 80.2)	-1.0 (-7.7, 10.4)
Romania	203.5 (121.1, 228.0)	0.9 (0.5, 1.1)	77193 (54855.5, 106084.2)	317.1 (227.2, 427.8)	126.5 (98.3, 199.2)	0.5 (0.4, 0.8)	67802.8 (46177.7, 94032.4)	285.1 (198.1, 391.9)	-37.8 (-54.2, 27.5)	-12.2 (-20.6, -2.3)	-50.8 (-64.2, 15.1)	-10.1 (-18.4, 2.6)
Russian Federation	1925.1 (1150.7, 2093.8)	1.2 (0.7, 1.3)	297455.5 (221391.4, 387846.1)	191.3 (144.1, 248.2)	1628.5 (1353.4, 2477.6)	0.9 (0.8, 1.4)	209287.4 (160094.9, 272853.2)	134.8 (102.9, 175.4)	-15.4 (-31.3, 69.0)	-29.6 (-37.1, -13.4)	-24.9 (-38.6, 47.1)	-29.5 (-37.2, -12.2)
Rwanda	66.1 (40.2, 86.7)	1.6 (1.1, 2.1)	13722.6 (10012.2, 18094.9)	267.8 (191.7, 356.2)	112.3 (69.1, 153.9)	1.6 (1.0, 2.2)	24481.5 (17553.8, 33153.6)	256.3 (184.5, 343.6)	69.9 (12.8, 167.9)	78.4 (55.0, 103.3)	0.5 (-26.1, 40.2)	-4.3 (-13.0, 5.4)
Saint Kitts and Nevis	3.9 (2.4, 4.4)	10.8 (6.7, 12.2)	204.9 (149.4, 239.7)	538.9 (393.6, 633.6)	4.2 (3.0, 7.0)	7.6 (5.6, 12.2)	241.4 (175.5, 345.3)	388.9 (287.6, 564.8)	9.6 (-24.0, 130.9)	17.8 (-12.9, 98.0)	-29.8 (-49.7, 45.4)	-27.8 (-46.2, 23.5)
Saint Lucia	7.3 (4.5, 8.3)	7.4 (4.5, 8.4)	502.3 (362.5, 600.3)	422.6 (307.0, 502.7)	11.2 (8.8, 18.0)	5.9 (4.6, 9.4)	658.0 (515.9, 863.3)	351.2 (274.1, 461.0)	53.9 (15.3, 227.6)	31.0 (7.5, 106.2)	-21.3 (-40.5, 67.0)	-16.9 (-31.4, 30.2)

Saint Vincent and the Grenadines	7.7 (5.0, 8.9)	9.3 (6.1, 10.6)	500.8 (348.4, 603.3)	515.2 (372.3, 619.3)	11.9 (9.7, 17.7)	10.1 (8.1, 14.9)	584.9 (474.3, 766.6)	501.6 (404.2, 668.2)	53.8 (16.2, 183.4)	16.8 (-7.4, 84.4)	8.0 (-17.4, 93.7)	-2.6 (-22.3, 49.5)
Samoa	4.4 (3.3, 5.9)	4.9 (3.6, 6.6)	354.1 (268.6, 451.3)	299.0 (228.0, 381.2)	6.0 (4.1, 8.0)	4.1 (2.8, 5.4)	464.3 (350.5, 603.8)	263.2 (200.2, 339.4)	34.1 (-4.2, 85.4)	31.1 (8.2, 56.8)	-17.5 (-40.3, 12.1)	-12.0 (-27.3, 5.0)
San Marino	0.4 (0.3, 0.5)	1.4 (1.1, 1.9)	95.7 (66.5, 130.0)	356.0 (249.4, 475.7)	1.1 (0.7, 1.6)	1.7 (1.1, 2.5)	152.6 (107.1, 207.1)	355.2 (251.0, 479.7)	177.1 (66.4, 339.7)	59.4 (47.0, 74.9)	26.2 (-24.2, 98.3)	-0.2 (-8.0, 8.7)
Sao Tome and Principe	2.3 (1.8, 2.9)	3.1 (2.2, 3.9)	314.2 (234.6, 408.3)	319.0 (238.6, 415.6)	3.9 (2.8, 5.2)	3.8 (2.5, 5.1)	509.7 (380.0, 670.8)	317.1 (241.5, 410.5)	69.4 (27.1, 129.2)	62.2 (39.9, 87.3)	20.0 (-10.9, 59.5)	-0.6 (-10.0, 12.0)
Saudi Arabia	341.1 (253.8, 458.8)	4.2 (2.7, 6.0)	50663.4 (37911.3, 67100.4)	389.4 (288.8, 512.5)	850.1 (566.6, 1151.6)	4.7 (3.1, 6.2)	118239.4 (87317.9, 156224.2)	376.4 (285.1, 484.4)	149.3 (71.1, 279.0)	133.4 (93.7, 174.8)	11.7 (-28.3, 67.9)	-3.3 (-14.3, 10.0)
Senegal	90.8 (55.7, 115.7)	2.0 (1.2, 2.6)	19041.4 (13783.2, 24933.9)	306.9 (222.1, 406.0)	141.6 (102.3, 183.0)	1.6 (1.2, 2.0)	34863.1 (25117.0, 46632.9)	285.1 (205.5, 378.8)	55.9 (11.5, 138.2)	83.1 (62.7, 105.4)	-19.7 (-41.3, 22.1)	-7.1 (-14.9, 1.2)
Serbia	134.0 (108.0, 170.2)	1.5 (1.2, 1.9)	32689.2 (23589.5, 43889.7)	331.3 (243.1, 438.1)	149.7 (102.3, 192.3)	1.2 (0.8, 1.5)	33423.9 (23795.5, 45525.0)	315.6 (222.3, 427.2)	11.7 (-21.4, 50.8)	2.2 (-7.1, 11.3)	-21.8 (-45.4, 6.4)	-4.7 (-15.7, 4.9)
Seychelles	1.5 (0.9, 1.7)	2.5 (1.6, 2.9)	141.5 (104.5, 181.3)	225.3 (166.7, 288.9)	2.2 (1.7, 2.8)	2.1 (1.7, 2.8)	225.1 (168.5, 298.3)	194.6 (147.2, 253.4)	47.6 (17.7, 140.3)	59.1 (40.5, 91.6)	-14 (-31.2, 39.8)	-13.6 (-22.1, 2.2)

Sierra Leone	47.0 (27.5, 66.6)	1.7 (1.0, 2.2)	8913.4 (6467.6, 11678.2)	280.9 (203.4, 374.5)	76.6 (50.3, 109.5)	1.4 (1.0, 1.9)	18167.4 (13054.7, 24202.6)	270.3 (193.7, 363.4)	63.1 (14.2, 140.5)	103.8 (77.0, 134.8)	-15.2 (-39.6, 21.0)	-3.8 (-12.3, 5.5)
Singapore	24.1 (18.9, 27.5)	0.9 (0.8, 1.1)	9807.4 (6921.0, 13530.6)	324.1 (231.7, 443.1)	39.5 (32.6, 68.1)	0.6 (0.5, 1.0)	21613.6 (14547.9, 29972.0)	307.2 (209.5, 424.8)	63.4 (30.2, 188.0)	120.4 (102.7, 140.9)	-37.7 (-49.7, 1.0)	-5.2 (-11.4, 2.2)
Slovakia	43.7 (37.7, 57.9)	0.8 (0.7, 1.1)	25396.7 (17859.6, 34626.3)	462.1 (326.7, 631.3)	61.9 (40.2, 81.8)	0.9 (0.6, 1.1)	28637.7 (20012.3, 39180.4)	426.1 (300.5, 582.9)	41.6 (-9.3, 96.2)	12.8 (6.1, 21.6)	4.8 (-32.4, 44.9)	-7.8 (-13.6, -0.5)
Slovenia	27.0 (20.3, 34.8)	1.3 (1.0, 1.6)	7989.3 (5790.9, 10648.6)	381.6 (278.6, 506.9)	56.2 (36.5, 74.4)	1.6 (1.1, 2.1)	10050.4 (7341.0, 13388.9)	379.9 (278.1, 506.6)	108.5 (41.7, 187.1)	25.8 (18.0, 33.9)	24.1 (-10.4, 63.2)	-0.4 (-6.0, 5.2)
Solomon Islands	6.8 (5.0, 9.2)	4.1 (3.0, 5.7)	692.3 (527.4, 881.4)	290.3 (222.5, 369.7)	17.1 (11.8, 23.1)	4.6 (3.4, 6.1)	1542.7 (1160.6, 1974.8)	303.3 (231.1, 384.5)	151.6 (82.6, 239.2)	122.8 (95.7, 156.4)	13.9 (-15.8, 54.8)	4.5 (-9.2, 21.6)
Somalia	56.7 (33.7, 83.6)	1.5 (0.9, 2.4)	15326.8 (11014.1, 20564.5)	288.0 (208.3, 387.4)	156.1 (90.2, 315.4)	1.7 (1.0, 3.6)	39870.0 (28416.4, 54410.4)	282.1 (200.8, 386.4)	175.1 (81.7, 315.4)	160.1 (133.2, 197.2)	12.1 (-22.0, 60.2)	-2.0 (-9.5, 8.7)
South Africa	1533.9 (1182.5, 1778.6)	5.6 (4.4, 6.6)	180557.6 (141951.0, 225527.1)	590.9 (462.5, 738.5)	3213.3 (2529.6, 3735.3)	6.9 (5.4, 7.9)	304455.8 (236422.4, 381023.2)	573.8 (447.8, 716.6)	109.5 (85.9, 141.8)	68.6 (56.6, 88.7)	23.2 (10.1, 39.4)	-2.9 (-7.9, 5.6)
South Sudan	47.2 (27.5, 67.3)	1.3 (0.8, 1.7)	12845.5 (9261.9, 17054.1)	290.0 (205.5, 390.2)	76.9 (46.8, 112.5)	1.5 (0.9, 2.2)	20445.1 (14442.1, 27498.6)	291.4 (204.3, 391.5)	62.9 (18.3, 126.6)	59.2 (41.8, 76.4)	18.7 (-11.4, 56.2)	0.5 (-6.4, 8.0)

Spain	897.5 (782.7, 1217.6)	2.0 (1.7, 2.6)	159315.9 (115324.9, 214014.5)	369.1 (270.7, 488.4)	2690.6 (1686.3, 3090.9)	2.9 (1.9, 3.3)	233634.6 (171558.0, 308386.1)	377.5 (278.4, 498.5)	199.8 (65.1, 248.6)	46.6 (34.4, 56.2)	47.6 (-13.7, 67.8)	2.3 (-5.0, 7.7)
Sri Lanka	174.8 (137.3, 201.0)	1.6 (1.2, 1.8)	29307.9 (21508.5, 38288.1)	194.0 (142.9, 252.3)	273.6 (195.6, 368.6)	1.2 (0.9, 1.6)	40093.3 (28972.0, 53788.1)	164.7 (119.6, 220.0)	56.5 (15.4, 109.7)	36.8 (22.1, 50.5)	-24.2 (-44.2, 1.7)	-15.1 (-23.2, -7.1)
Sudan	378.4 (198.4, 671.9)	1.8 (1.2, 2.6)	67436.2 (45332.1, 97910.3)	338.1 (236.3, 457.2)	843.9 (501.0, 1265.3)	2.9 (1.8, 4.0)	131343.0 (93268.5, 177482.5)	357.1 (258.4, 473.8)	123.0 (21.5, 281.9)	94.8 (42.5, 153.0)	56.4 (-3.3, 133.5)	5.6 (-12.6, 28.1)
Suriname	16.6 (11.2, 20.0)	5.2 (3.6, 6.2)	1395.6 (990.3, 1737.1)	395.4 (289.8, 488.2)	26.7 (20.5, 38.3)	4.8 (3.7, 6.9)	1992.8 (1554.7, 2589.1)	347.4 (270.7, 447.6)	60.7 (13.7, 159.8)	42.8 (10.7, 94.7)	-8.2 (-34.2, 47.1)	-12.2 (-30.3, 17.8)
Sweden	247.1 (121.6, 282.2)	1.8 (0.9, 2.0)	34967.8 (24914.3, 46856.6)	334.7 (240.6, 448.9)	363.9 (216.8, 416.3)	1.7 (1.1, 1.9)	44293.3 (31622.1, 60325.6)	330.6 (233.5, 447.5)	47.3 (32.2, 81.0)	26.7 (20.0, 34.0)	-4.0 (-13.5, 26.8)	-1.2 (-6.2, 4.7)
Switzerland	208.2 (174.3, 262.5)	2.3 (1.9, 2.9)	24971.4 (18816.0, 32206.4)	305.5 (233.4, 392.1)	343.8 (283.6, 459.9)	2.2 (1.8, 3.0)	36673.4 (27588.7, 47716.6)	302.6 (229.2, 393.5)	65.1 (42.8, 96.6)	46.9 (40.5, 55.3)	-2.5 (-14.7, 17.3)	-1.0 (-6.2, 5.9)
Syrian Arab Republic	303.8 (202.2, 433.9)	2.6 (1.9, 3.3)	45634.8 (33058.7, 61375.3)	363.4 (271.9, 471.4)	231.3 (170.3, 313.9)	2.2 (1.6, 2.9)	43415.9 (31823.0, 57900.0)	309.4 (229.2, 407.4)	-23.9 (-48.8, 24.4)	-4.9 (-26.1, 20.1)	-17.7 (-40.2, 16.0)	-14.9 (-26.4, -2.2)
Taiwan (Province of China)	351.6 (256.3, 383.4)	2.4 (1.8, 2.7)	48640.1 (36063.6, 63908.3)	261.7 (194.2, 342.6)	688.5 (518.6, 1004.1)	1.9 (1.4, 2.8)	65852.1 (48297.8, 87703.4)	216.1 (158.9, 284.2)	95.8 (49.0, 205.1)	35.4 (21.0, 52.8)	-21.4 (-40.3, 22.2)	-17.4 (-25.5, -5.9)

Tajikistan	24.7 (19.8, 38.8)	0.5 (0.4, 0.6)	9257.7 (6667.2, 12614.8)	206.7 (146.1, 283.0)	62.3 (35.5, 83.5)	0.8 (0.4, 1.1)	18158.8 (13052.4, 24332.7)	212.7 (151.7, 285.2)	152.4 (13.8, 274.6)	96.1 (60.2, 120.1)	78.3 (-8.6, 153.8)	2.9 (-9.5, 13.5)
Thailand	1111.2 (561.6, 1532.4)	2.6 (1.4, 3.5)	113862.2 (75927.8, 151966.6)	227.5 (154.1, 300.9)	1490.9 (1064.9, 2215.6)	1.6 (1.2, 2.4)	159083.7 (116895.8, 213256.5)	177.4 (132.0, 234.1)	34.2 (-23.6, 204.2)	39.7 (8.7, 91.0)	-37.0 (-62.9, 32.3)	-22 .0 (-38.4, 5.7)
Timor-Leste	9.5 (5.2, 15.2)	1.8 (1.3, 2.3)	1341.2 (831.2, 1920.0)	193.6 (138.7, 251.8)	13.2 (8.8, 18.1)	1.6 (1.1, 2.3)	1808.4 (1321.6, 2394.5)	167.9 (122.4, 223.0)	39.5 (-21.6, 194.8)	34.8 (-5.8, 97.6)	-8.7 (-36.9, 40.7)	-13.3 (-28.1, 6.2)
Togo	29.1 (18.8, 39.0)	1.6 (1.0, 2.2)	7164.0 (5236.2, 9534.9)	269.2 (193.8, 362.3)	59.8 (43.5, 77.6)	1.4 (1.0, 1.8)	16640.8 (11945.2, 22546.5)	264.7 (189.4, 357.6)	105.8 (46.2, 197.9)	132.3 (106.4, 156.2)	-11.8 (-38.0, 35.5)	-1.7 (-9.7, 6.8)
Tokelau	0.0 (0.0, 0.1)	3.4 (2.2, 4.9)	4.0 (3.0, 5.3)	278.0 (207.2, 371.5)	0.0 (0.0, 0.1)	3.3 (2.2, 4.6)	3.4 (2.6, 4.5)	251.7 (189.0, 330.5)	-6.8 (-32.6, 30.4)	-13.7 (-24.9, -1.1)	-1.9 (-29.3, 39.6)	-9.5 (-20.9, 4.1)
Tonga	1.8 (1.3, 2.5)	3.2 (2.2, 4.4)	190.2 (144.7, 247.5)	259.4 (195.6, 338.1)	2.9 (2.0, 4.1)	3.6 (2.5, 5.1)	235.0 (178.7, 307.3)	263.2 (199.6, 343.8)	62.9 (23.9, 115.7)	23.5 (10.2, 41.2)	13.7 (-14.2, 51.2)	1.5 (-9.5, 16.4)
Trinidad and Tobago	96.2 (63.1, 107.0)	11.2 (7.0, 12.5)	5482.8 (4304.8, 6329.0)	524.0 (411.7, 609.6)	129.5 (91.4, 210.1)	8.0 (5.6, 13.2)	6714.2 (5049.1, 9230.3)	436.0 (326.2, 623.3)	34.6 (-8.7, 164.9)	22.5 (-4.4, 84.8)	-29.1 (-52.5, 46.1)	-16.8 (-35.5, 32.6)
Tunisia	132.5 (99.2, 183.7)	1.9 (1.4, 2.6)	25682.8 (19238.6, 33930.3)	328.7 (243.1, 439.2)	237.4 (150.1, 329.5)	2.2 (1.4, 3.0)	37667.4 (27073.1, 50113.0)	311.5 (225.4, 409.5)	79.1 (2.6, 170.2)	46.7 (20.0, 68.3)	14.4 (-29.9, 69.5)	-5.2 (-18.9, 7.2)

Turkey	1784.6 (1091.8, 3592.7)	3.3 (2.1, 6.5)	253906.4 (180653.9, 402079.6)	445.8 (323.6, 652.1)	2523.6 (1985.2, 3269.2)	3.3 (2.7, 4.2)	344955.8 (254330.1, 451813.1)	424.6 (321.8, 552.5)	41.4 (-18.3, 134.8)	35.9 (-12.5, 71.9)	0.5 (-42.0, 64.1)	-4.8 (-31.2, 16.5)
Turkmenistan	23.9 (19.0, 29.6)	0.6 (0.5, 0.9)	7367.8 (5509.4, 9681.2)	229.1 (166.7, 307.2)	72.2 (52.2, 92.1)	1.5 (1.1, 1.9)	13506.8 (10111.9, 17485.1)	269.3 (201.4, 347.9)	202.6 (132.4, 278.9)	83.3 (66.1, 105.0)	140.2 (76.0, 202.0)	17.6 (6.7, 31.7)
Tuvalu	0.3 (0.2, 0.4)	4.1 (2.7, 5.7)	26.0 (20.0, 33.1)	307.9 (236.8, 399.9)	0.4 (0.2, 0.5)	3.7 (2.5, 5.2)	29.7 (22.1, 38.5)	266.9 (199.9, 343.6)	28.2 (-6.8, 77.2)	14.1 (-1.8, 34.1)	-9.3 (-34.1, 27.8)	-13.3 (-24.4, 0.8)
Uganda	108.2 (66.1, 153.7)	1.1 (0.7, 1.6)	30246.9 (21953.9, 39962.1)	243.4 (173.7, 326.4)	253.9 (157.2, 362.5)	1.3 (0.9, 1.9)	68077.6 (48831.2, 92812.7)	248.5 (178.0, 336.9)	134.7 (72.2, 222.4)	125.1 (102.8, 149.8)	22.4 (-5.5, 59.0)	2.1 (-4.9, 9.4)
Ukraine	204.8 (179.2, 342.7)	0.4 (0.3, 0.6)	99140.9 (68812.3, 136119.5)	171.5 (120.1, 235.4)	450.0 (332.8, 542.7)	0.9 (0.6, 1.1)	98218.4 (71857.3, 130219.0)	200.2 (148.3, 264.5)	119.7 (28.4, 179.1)	-0.9 (-9.8, 7.8)	163.3 (41.5, 235.4)	16.7 (2.1, 30.9)
United Arab Emirates	25.4 (15.1, 35.2)	2.8 (1.3, 4.0)	5755.1 (4204.8, 7659.4)	374.3 (275.2, 491.9)	207.2 (85.5, 385.1)	4.1 (1.7, 7.1)	33639.2 (22304.6, 46310.2)	380.9 (262.1, 513.0)	714.6 (343.7, 1265.3)	484.5 (381.9, 627.1)	48.3 (-11.7, 127.5)	1.7 (-11.0, 22.0)
United Kingdom	2133.4 (1590.7, 2311.8)	2.8 (2.0, 2.9)	254100.0 (189990.4, 329674.3)	383.2 (287.4, 494.6)	2359.6 (2023.4, 3757.3)	2.3 (2.0, 3.5)	335897.0 (247420.9, 446251.6)	399.9 (297.6, 520.3)	10.6 (-2.8, 83.5)	32.2 (26.3, 44.4)	-17.2 (-25.7, 29.1)	4.4 (0.2, 13.7)
United Republic of Tanzania	185.0 (142.3, 235.6)	1.1 (0.9, 1.4)	54388.1 (39365.1, 71941.0)	273.4 (197.8, 364.3)	460.6 (349.1, 604.3)	1.4 (1.1, 1.8)	118101.8 (84950.0, 160296.2)	272.3 (197.0, 366.9)	148.9 (86.1, 233.2)	117.1 (95.4, 140.5)	23.8 (-1.4, 54.2)	-0.4 (-6.4, 6.1)

United States of America	8683.7 (7449.0, 12182.6)	2.9 (2.5, 4.0)	902395.2 (697072.7, 1149230.2)	323.3 (250.3, 411.5)	34541.9 (24721.7, 37438.3)	6.5 (4.9, 7.3)	1499101.1 (1232165.6, 1795810.6)	350.2 (289.6, 421.8)	297.8 (181.0, 332.1)	66.1 (49.3, 86.1)	123.1 (68.0, 138.8)	8.3 (-1.4, 19.8)
United States Virgin Islands	5.8 (4.0, 7.2)	6.9 (4.8, 8.5)	408.6 (318.9, 512.7)	408.2 (320.7, 508.8)	10.4 (8.1, 13.0)	6.8 (5.4, 8.6)	513.4 (408.8, 644.1)	366.8 (292.2, 461.7)	80.7 (31.6, 150.2)	25.6 (3.9, 50.8)	-0.2 (-26.8, 37.6)	-10.1 (-26.2, 8.5)
Uruguay	131.5 (85.2, 146.7)	3.7 (2.4, 4.1)	13158.2 (9878.6, 17225.6)	388.3 (293.4, 503.1)	179.8 (151.8, 250.1)	3.4 (2.9, 4.9)	15344.6 (11568.1, 20422.1)	371.0 (276.2, 491.8)	36.8 (14.9, 131.5)	16.6 (8.9, 35.1)	-6.6 (-21.5, 58.2)	-4.5 (-11.4, 12.9)
Uzbekistan	77.6 (64.5, 101.6)	0.4 (0.3, 0.6)	40305.2 (28993.6, 54642.8)	226.0 (159.7, 307.8)	194.0 (156.9, 285.8)	0.8 (0.6, 1.0)	73443.3 (52484.0, 99461.6)	229.3 (164.7, 308.7)	149.8 (103.4, 211.7)	82.2 (68.3, 97.5)	96.5 (62.5, 134.5)	1.5 (-4.6, 8.6)
Vanuatu	2.5 (1.6, 3.7)	3.4 (2.0, 5.1)	278.3 (208.3, 370.2)	258.9 (190.6, 346.5)	7.2 (4.8, 10.2)	4.0 (2.6, 5.8)	675.9 (506.1, 882.7)	284.6 (213.0, 369.3)	185.6 (105.3, 307.3)	142.9 (109.0, 180.2)	16.4 (-16.1, 68.3)	9.9 (-5.5, 28.6)
Venezuela (Bolivarian Republic of)	179.2 (137.5, 207.0)	1.3 (1.1, 1.6)	32490.4 (24610.1, 41920.0)	226 (168.6, 296.2)	422.9 (311.1, 616.3)	1.5 (1.1, 2.2)	63054.8 (45793.9, 84076.5)	215.8 (158.1, 288.6)	136 (71.6, 241.5)	94.1 (72.9, 120.3)	11.9 (-17.2, 53.7)	-4.5 (-13.3, 7.1)
Viet Nam	617.0 (340.7, 848.7)	1.3 (0.8, 1.7)	96994.2 (68970.3, 129306.4)	179.4 (126.4, 240.6)	1154.1 (620.6, 1589.9)	1.3 (0.7, 1.7)	161682.8 (111858.0, 221135.8)	153.3 (108.5, 209.3)	87.1 (34.4, 168.4)	66.7 (45.7, 90.8)	-1.4 (-30.4, 39.3)	-14.5 (-22.7, -3.5)
Yemen	152.2 (97.9, 258.3)	1.3 (0.9, 1.8)	38502.9 (28270.5, 52069.0)	310.3 (223.0, 416.5)	330 (195.6, 493.7)	1.6 (0.9, 2.5)	90667.9 (66159.1, 122767.7)	330.5 (239.5, 447.0)	116.9 (0.1, 301.5)	135.5 (81.8, 186.4)	29.4 (-28.7, 100.4)	6.5 (-7.1, 21.1)

Zambia	56.9 (41.2, 75.4)	1.3 (1.0, 1.6)	16659.3 (12160.6, 22088.2)	286.0 (204.8, 380.7)	109.9 (84.1, 144.4)	1.3 (1.0, 1.7)	38409.6 (27523.7, 51834.4)	286.3 (204.5, 386.2)	93.3 (32.7, 181.3)	130.6 (105.8, 155.4)	1.0 (-23.8, 31.7)	0.1 (-6.2, 6.2)
Zimbabwe	99.2 (43.7, 132.3)	1.8 (0.8, 2.3)	25792.0 (18007.2, 34992.9)	354.0 (247.8, 482.2)	76.6 (47.1, 150.3)	0.8 (0.5, 1.7)	36867.4 (25743.9, 50417.8)	326.0 (225.1, 446.1)	-22.8 (-60.6, 162.9)	42.9 (22.7, 72.4)	-52.4 (-75.2, 60.6)	-7.9 (-17.7, 8.0)

EMBID: endocrine, metabolic, blood and immune disorders. **DALYs:** disability-adjusted life years.

Table S4: The EMBID-related YLLs, Age-standardized YLL rate and YLDs, Age-standardized YLD rate in 1990 and 2019, and percent change from 1990 to 2019.

	1990				2019				1990-2019		1990-2019	
	**YLLs**		**YLDs**		**YLLs**		**YLDs**		**Percent change in number (%)**		**Percent change in age-standardized rates (%)**	
	YLLs (95% UI)	Age-standardized YLL rate per100,000 (95% UI)	YLDs (95% UI)	Age-standardized YLD rate per 100,000 (95% UI)	YLLs (95% UI)	Age-standardized YLL rate per 100,000 (95% UI)	YLDs (95% UI)	Age-standardized YLD rate per 100,000 (95% UI)	**YLLs**	**YLDs**	**YLLs**	**YLDs**
Global	3590218.9 (2874542.6, 4329879.1)	67.5 (55.4, 80.2)	11022444.4 (7513643.2, 15340317.4)	232.6 (158.5, 323.1)	4906252.7 (4066139.1, 5534464.1)	64.2 (53.3, 72.7)	18000311.4 (24962910.0, 12249600.2)	221.8 (151.1, 307.9)	36.7 (13.4, 61.8)	63.3 (59.1, 67.5)	-4.9 (9.5, -18.5)	-4.6 (-3.4, -6.1)
**Countries**												
Afghanistan	20525.0 (8937.5, 35940.9)	127.8 (63.5, 206.6)	22997.7 (15287.7, 32537.2)	237.8 (161.1, 333.9)	50423.2 (30244.2, 72087.2)	116.6 (73.6, 158.6)	65321.8 (43302.6, 93843.7)	226.3 (149.9, 321.0)	145.7 (33.2, 410.5)	184.0 (160.9, 209.0)	-8.8 (-49.1, 64.1)	-4.9 (-10.1, 0.8)
Albania	4672.1 (3509.8, 8192.3)	140.2 (107.0, 216.5)	7365.6 (4915.7, 10508.8)	249.3 (165.0, 351.9)	2270.5 (1556.7, 3704.1)	86.5 (55.9, 163.9)	7889.2 (5288.4, 11364.5)	246.2 (163.3, 352.4)	-51.4 (-64.9, -27.0)	7.1 (-4.0, 19.6)	-38.3 (-59.2, -2.7)	-1.2 (-7.8, 6.1)

Algeria	24928.1 (15884.8, 40165.4)	87.9 (60.9, 131.2)	53467.3 (35747.2, 75799.3)	261.5 (174.1, 371.5)	33733.3 (22683.3, 42982.1)	85.8 (57.6, 109.3)	100045.9 (66270.2, 141132.0)	244.6 (163.0, 347.0)	35.3 (-28.1, 112.9)	87.1 (70.3, 105.1)	-2.4 (-44.2, 45.1)	-6.4 (-12.1, -1.0)
American Samoa	119.5 (86.4, 172.7)	349.8 (255.0, 490.7)	67.9 (45.1, 95.9)	182.6 (122.4, 255.5)	229.6 (157.0, 301.5)	450.3 (308.2, 589.3)	90.1 (60.9, 126.1)	168.4 (114.5, 233.8)	92.2 (27.5, 189.4)	32.7 (20.5, 43.7)	28.7 (-13.2, 91.7)	-7.8 (-13.5, -2.0)
Andorra	24.7 (18.2, 33.2)	52.6 (38.7, 72.2)	182.3 (121.2, 252.2)	312.7 (208.5, 435.3)	44.4 (31.7, 59.2)	43.5 (31.6, 58.7)	341.7 (225.9, 486.0)	304.5 (203.7, 428.3)	80.2 (24.2, 153.2)	87.5 (73.8, 102.1)	-17.4 (-42.9, 19.4)	-2.6 (-7.6, 2.9)
Angola	13453.8 (3991.0, 25988.8)	96.5 (37.1, 160.1)	15894.4 (10659.8, 22540.6)	221.5 (149.0, 310.1)	22865.4 (10002.2, 35901.9)	76.0 (32.1, 118.6)	47753.1 (32027.1, 67989.5)	225.2 (151.8, 316.4)	70.0 (-6.4, 260.3)	200.4 (181.5, 222.2)	-21.2 (-53.5, 28.4)	1.7 (-3.5, 7.5)
Antigua and Barbuda	163.2 (112.6, 188.2)	279.8 (192.2, 321.7)	94.4 (63.6, 132.6)	174.9 (117.9, 246.2)	243.4 (189.4, 362.7)	287.7 (217.8, 424.5)	168.0 (112.7, 236.0)	166.5 (112.3, 233.6)	49.1 (10.5, 159.8)	77.9 (64.4, 91.6)	2.8 (-24.9, 79.0)	-4.8 (-9.8, 0.8)
Argentina	56693.0 (28070.6, 64991.2)	175.9 (85.5, 201.1)	89938.5 (59573.2, 126584.7)	275.7 (182.7, 387.7)	40030.7 (32990.4, 76279.6)	88.5 (72.0, 169.4)	130407.6 (86998.9, 184050.2)	265.0 (176.1, 374.9)	-29.4 (-46.6, 121.0)	45.0 (37.5, 53.5)	-49.7 (-62.6, 61.4)	-3.9 (-8.7, 1.6)
Armenia	659.1 (563.5, 898.3)	19.2 (16.5, 26.2)	5752.2 (3817.0, 8144.1)	177.9 (118.0, 250.4)	1233.3 (822.8, 1487.7)	41.1 (28.6, 49.7)	6341.9 (4211.5, 8965.2)	178.4 (118.6, 250.3)	87.1 (26.0, 127.9)	10.3 (2.0, 19.2)	114 (50.2, 158.5)	0.3 (-5.1, 6.2)
Australia	14870.3 (12099.7, 19028.3)	86.9 (70.5, 110.4)	36803.1 (24485.8, 51476.6)	202.5 (134.6, 283.2)	40003.6 (33131.3, 51439.6)	130.2 (110.2, 174.7)	60952.2 (40896.0, 85656.5)	200.3 (135.1, 281.3)	169.0 (147.6, 190.0)	65.6 (56.2, 75.6)	49.8 (37.3, 65.2)	-1.1 (-5.8, 3.7)

Austria	10235.7 (8984.8, 14221.2)	121.2 (104.8, 173.5)	44330.3 (30288.8, 61022.3)	475.3 (323.8, 658.7)	22994.1 (15260.5, 25635.7)	191.6 (135.7, 215.9)	54347.5 (37156.2, 75176.3)	445.4 (303.6, 615.2)	124.6 (29.4, 171.3)	22.6 (16.5, 29.6)	58 (-3.2, 90.4)	-6.3 (-11.1, -1.1)
Azerbaijan	8324.2 (4282.5, 11158.7)	97.8 (54.6, 128.6)	12764.7 (8682.3, 17958.5)	198.0 (134.6, 277.6)	8991.4 (5013.9, 12836.7)	107.5 (54.7, 159.3)	21276.0 (14083.6, 29823.7)	189.7 (125.1, 266.7)	8.0 (-20.7, 49.9)	66.7 (54.3, 79.6)	9.9 (-19.6, 52.0)	-4.2 (-10.0, 1.1)
Bahamas	1349.5 (864.0, 1570.7)	595.0 (390.2, 687.4)	369.8 (250.0, 519.7)	184.3 (124.8, 260.2)	1683.8 (1281.7, 2440.7)	472.2 (362.1, 689.8)	702.6 (469.0, 985.9)	172.8 (116.8, 243.1)	24.8 (-7.3, 111.7)	90.0 (75.3, 105.2)	-20.6 (-41.3, 35.5)	-6.2 (-11.4, -0.6)
Bahrain	803.5 (623.2, 1186.0)	197.7 (160.5, 260.8)	1146.2 (757.3, 1647.7)	274.5 (184.0, 386.4)	1961.2 (1254.5, 2525.1)	208.2 (131.3, 262.9)	4011.2 (2602.9, 5759.0)	255.8 (171.1, 361.6)	144.1 (31.0, 244.7)	250.0 (209.1, 290.2)	5.3 (-37.3, 39.8)	-6.8 (-12.0, -2.0)
Bangladesh	67153.7 (40202.1, 103510.9)	46.7 (30.6, 68.7)	158278.1 (105496.1, 221573.9)	202.9 (136.6, 281.1)	46785.2 (35100.8, 62359.2)	32.9 (24.7, 43.6)	275580.3 (185006.5, 384813.5)	183.8 (123.8, 256.0)	-30.3 (-56.1, 29.9)	74.1 (60.7, 88.2)	-29.6 (-53.4, 21.9)	-9.4 (-14.5, -3.7)
Barbados	806.4 (561.5, 909.6)	322.3 (222.7, 367.6)	440.0 (297.0, 617.0)	170.1 (114.4, 239.9)	921.1 (695.9, 1435.0)	276.6 (205.1, 441.1)	634.9 (426.8, 905.8)	157.1 (105.6, 222.3)	14.2 (-17.3, 99.6)	44.3 (34.1, 55.4)	-14.2 (-39.5, 53.9)	-7.7 (-12.4, -2.3)
Belarus	1619.7 (1371.8, 2765.3)	16.6 (13.9, 27.9)	15815.1 (10684.4, 22109.3)	140.1 (94.4, 196.2)	4657.7 (2557.2, 6189.6)	48.7 (26.9, 65.9)	15473.7 (10377.6, 21869.5)	135.8 (91.4, 193.9)	187.6 (14.8, 322.8)	-2.2 (-9.0, 5.2)	194.1 (22.9, 339.8)	-3.0 (-9.4, 3.9)
Belgium	10433.0 (8399.4, 12483.4)	98.0 (78.2, 116.7)	42038.7 (28687.8, 59276.8)	349.5 (236.2, 489.1)	13874.3 (11022.9, 17401.8)	94.6 (77.5, 124.4)	54362.8 (36793.4, 76017.5)	365.5 (246.5, 509.3)	33.0 (20.6, 54.9)	29.3 (23.1, 37.0)	-3.5 (-14.6, 18.0)	4.6 (-0.5, 10.8)

Belize	694.6 (300.3, 875.7)	321.7 (162.3, 389.7)	211.3 (142.8, 297.3)	161.9 (109.5, 227.8)	912.7 (733.8, 1346.4)	249.9 (199.9, 374.9)	552.9 (370.8, 770.6)	159.0 (106.5, 221.4)	31.4 (-6.8, 263.2)	161.6 (140.3, 182.1)	-22.3 (-43.3, 79.3)	-1.8 (-7.5, 4.0)
Benin	3234.8 (1895, 4583.3)	60.9 (38.0, 77.8)	7277.9 (4899.5, 10247.9)	214.6 (145.5, 300.9)	7578.1 (4619.1, 11792.1)	60.9 (40.1, 88.3)	19460.6 (13113.7, 27223.3)	214.9 (144.1, 298.4)	134.3 (51.0, 258.1)	167.4 (150.3, 186.3)	0.0 (-32.7, 47.7)	0.2 (-4.7, 5.3)
Bermuda	171.0 (114.8, 194.7)	292.3 (198.1, 334.8)	115.5 (76.8, 160.7)	182.0 (120.8, 254.3)	134.4 (104.7, 200.8)	161.0 (121.6, 260.7)	169.5 (112.6, 244.3)	171.1 (114.4, 241.6)	-21.4 (-40.8, 33.8)	46.8 (36.3, 58.6)	-44.9 (-59.6, 0.0)	-6.0 (-11.0, -0.9)
Bhutan	412.9 (140.3, 808.5)	54.7 (22.8, 97.2)	1037.8 (686.6, 1484.6)	224.7 (150.9, 316.1)	371.1 (232.5, 543.5)	56.2 (35.6, 81.4)	1532.4 (1030.8, 2127.6)	220.0 (149.4, 302.6)	-10.1 (-54.2, 165.6)	47.7 (33.6, 73.9)	2.7 (-42.1, 143.3)	-2.1 (-8.8, 9.2)
Bolivia (Plurinational State of)	10747.6 (6814.2, 14892.2)	138.2 (90.6, 179.8)	9515.8 (6454.5, 13153.8)	205.2 (140.5, 287.6)	9286.7 (6331.3, 14020.8)	80.9 (56.9, 120.6)	19620.0 (13276.2, 27409.2)	187.6 (126.4, 264.3)	-13.6 (-50.5, 58.1)	106.2 (90.2, 122.0)	-41.4 (-63.9, 1.1)	-8.6 (-14.3, -3.1)
Bosnia and Herzegovina	1547.9 (1247.2, 1819.3)	36.0 (29.9, 43.1)	11429.2 (7607.9, 16181.4)	250.1 (165.8, 355.8)	1121.9 (839.5, 1464.6)	29.2 (22.5, 39.5)	10172.0 (6810.9, 14733.5)	245.5 (164.1, 347.7)	-27.5 (-44.7, -6.7)	-11.0 (-19.0, -2.6)	-18.8 (-38.2, 4.7)	-1.8 (-8.2, 4.4)
Botswana	965.1 (463.4, 1601.0)	100.3 (49.0, 169.7)	3128.2 (2139.0, 4398.0)	336.5 (228.3, 472.1)	3066.2 (1539.0, 4615.6)	149.4 (76.7, 223.4)	7854 (5318.5, 11017.3)	394.8 (266.2, 553.9)	217.7 (95.4, 398.1)	151.1 (134.0, 168.7)	48.9 (-6.9, 137.7)	17.3 (11.0, 24.0)
Brazil	85218.3 (74696.1, 115744.2)	64.3 (57.2, 88.5)	171407.5 (116867.3, 236883.3)	141.8 (96.8, 196.1)	302126.3 (189011.5, 333041.1)	138.8 (88.4, 154.4)	229269.0 (157042.5, 321069.9)	98.7 (67.8, 138.1)	254.5 (106.0, 316.4)	33.8 (25.6, 41.7)	115.9 (24.7, 152.5)	-30.4 (-33.9, -26.8)

Brunei Darussalam	536.7 (432.7, 735.7)	238.4 (196.0, 315.4)	634.2 (423.0, 912.1)	295.2 (197.9, 416.1)	868.9 (715.8, 1151.4)	243.2 (201.4, 310.2)	1285.4 (849.5, 1827.1)	280.9 (187.5, 397.8)	61.9 (33.0, 100.6)	102.7 (86.1, 121.1)	2.0 (-16.1, 25.2)	-4.8 (-9.7, 0.6)
Bulgaria	3819.8 (3134.2, 4902.3)	50.6 (40.0, 63.0)	26399.9 (17609.2, 37537.2)	262.0 (174.9, 371.8)	2895.3 (2183.3, 4088.6)	43.5 (32.3, 64.8)	22635.2 (15187.9, 32104.8)	251.9 (169.3, 354.8)	-24.2 (-41.9, -0.6)	-14.3 (-20.0, -7.8)	-14.2 (-35.2, 17.2)	-3.9 (-9.2, 2.6)
Burkina Faso	4115.7 (2951.2, 5471.4)	40.6 (30.8, 51.8)	15441 (10535.6, 21582.0)	215.1 (145.9, 301.1)	12262.5 (8530.7, 16938.0)	53.9 (39.6, 69.9)	40616 (27482.1, 57433.8)	231.9 (157.0, 323.1)	197.9 (115.1, 326.8)	163 (141.0, 187.2)	32.6 (3.4, 70.9)	7.8 (1.1, 14.8)
Burundi	2382.7 (1348.9, 3358.0)	44.1 (25.9, 58.8)	8041.6 (5493.1, 11359.8)	210.8 (140.9, 295.1)	3631.0 (2037.0, 6395.8)	37.4 (21.4, 60.4)	16712.1 (11151.8, 23709.5)	200.4 (134.9, 281.2)	52.4 (-15.7, 163.0)	107.8 (93.8, 122.7)	-15.2 (-46.2, 32.3)	-4.9 (-10.0, 0.4)
Cabo Verde	78.6 (53.0, 121.1)	20.3 (15.4, 28.1)	649.1 (436.9, 916.1)	235.5 (157.4, 330.5)	120.8 (91.4, 154.8)	24.0 (18.4, 30.8)	1219.4 (816.1, 1726.8)	235.5 (158.2, 331.9)	53.7 (-2.1, 123.7)	87.9 (73.3, 103.1)	18.3 (-15.4, 56.4)	0.0 (-5.7, 6.0)
Cambodia	8522.8 (4322.7, 14080.0)	77.3 (50.0, 108.2)	11517.7 (7804.2, 16210.2)	152.0 (102.3, 214.0)	8233.3 (6371.9, 10858.1)	57.1 (44.3, 73.2)	20689.3 (13825.0, 29044.2)	134.7 (89.7, 187.6)	-3.4 (-42.8, 98.0)	79.6 (64.3, 95.6)	-26.2 (-50.2, 21.3)	-11.4 (-17.4, -5.4)
Cameroon	7479.3 (4286.3, 10424.5)	82.1 (47.8, 109.3)	16479.5 (11062.7, 23126.4)	220.8 (149.9, 305.3)	17306.8 (10206.6, 26546.0)	71.5 (44.0, 105.9)	46326.0 (30670.7, 65742.8)	216.2 (144.7, 301.7)	131.4 (51.5, 239.7)	181.1 (161.7, 202.7)	-12.8 (-40.9, 27.5)	-2.1 (-7.6, 3.9)
Canada	33382.5 (22283.9, 36683.5)	118.1 (79.4, 129.1)	58559.8 (39033.9, 82367.0)	190.5 (126.7, 268.5)	58104.9 (50069.8, 81923.7)	125.1 (109.1, 177.9)	95287.4 (63804.5, 135304.6)	186.4 (125.1, 262.9)	74.1 (50.4, 136.0)	62.7 (51.0, 74.0)	5.9 (-8.7, 45.0)	-2.2 (-6.5, 3.2)

Central African Republic	3307.8 (1043.1, 5692.3)	98.1 (38.3, 144.2)	4268.6 (2870.8, 6070.4)	213.1 (143.6, 298.5)	4688.3 (2039.0, 7691.0)	87.5 (37.7, 131.3)	8484.4 (5663.1, 12090.3)	215.4 (145.9, 303.2)	41.7 (-4.0, 123.1)	98.8 (83.1, 114.7)	-10.9 (-35.3, 25.8)	1.1 (-5.1, 6.8)
Chad	2757.6 (1740.5, 3709.2)	42.2 (26.4, 58.2)	10202.6 (6954.4, 14452.1)	225.1 (152.3, 315.9)	6125.0 (4363.3, 8298.2)	37.8 (27.4, 49.2)	25996.4 (17516.9, 36611.7)	222.0 (150.2, 308.1)	122.1 (54.2, 218.2)	154.8 (136.7, 172.9)	-10.5 (-34.5, 21.4)	-1.3 (-6.1, 3.8)
Chile	8089.6 (6544.1, 10035.9)	64.8 (53.0, 80.8)	27795.3 (18893.7, 38800.5)	238.5 (162.0, 331.3)	17100.0 (14296.5, 21664.1)	91.4 (76.3, 120.1)	52660.6 (36120.9, 72939.5)	242.2 (165.5, 335.1)	111.4 (88.2, 142.4)	89.5 (81.2, 98.0)	41.1 (23.9, 65.6)	1.5 (-2.7, 5.7)
China	648259.7 (474833.6, 790880.0)	59.3 (43.8, 72.2)	2379888.3 (1589502.3, 3348381.0)	225.1 (150.1, 317.3)	502183.7 (371606.2, 587478.8)	36.9 (26.7, 42.8)	3966668.1 (2639710.9, 5621743.3)	213.2 (141.9, 302.6)	-22.5 (-41.4, -1.1)	66.7 (55.2, 79.1)	-37.8 (-54.8, -20.6)	-5.3 (-9.1, -1.6)
Colombia	19357.1 (14004.8, 21782.2)	62.7 (46.7, 69.3)	40005.9 (26731.5, 56171.2)	170.6 (114.4, 241.2)	36579.4 (27067.0, 50049.3)	77.6 (57.3, 106.1)	85505.9 (57029.2, 120603.2)	167.1 (111.5, 235.8)	89.0 (40.4, 162.5)	113.7 (96.8, 132.6)	23.9 (-7.6, 69.8)	-2.1 (-7.9, 4.0)
Comoros	205.3 (102.3, 293.2)	46.6 (23.4, 65.0)	823.2 (553.6, 1163.7)	241.0 (164.3, 337.7)	297.5 (180.0, 420.5)	50.0 (30.8, 70.2)	1402.5 (930.1, 1962.7)	224.2 (150.6, 314.5)	44.9 (-6.1, 178.8)	70.4 (55.8, 97.1)	7.4 (-26.1, 107.2)	-7.0 (-12.8, 2.5)
Congo	2816.8 (958.3, 4688.8)	108.8 (43.3, 160.1)	4225.9 (2854.1, 5934.3)	240.5 (163.3, 338.7)	3776.3 (1714.4, 5625.4)	83.5 (37.2, 125.4)	9754.0 (6499.2, 13565.5)	229.0 (154.7, 317.2)	34.1 (-14.5, 125.6)	130.8 (113.7, 150.9)	-23.3 (-46.9, 10.6)	-4.8 (-10.1, 0.9)
Cook Islands	2.7 (1.8, 3.6)	15.5 (11.5, 20.7)	28.0 (18.6, 39.4)	172.6 (114.2, 241.7)	2.8 (2.1, 3.8)	14.2 (9.7, 19.1)	35.9 (23.8, 50.7)	171.5 (113.6, 238.3)	5.1 (-28.4, 53.7)	28.1 (18.0, 38.6)	-8.5 (-38.2, 32.1)	-0.7 (-6.1, 5.0)

Costa Rica	1226.1 (1002.9, 1551.5)	41.5 (34.7, 53.3)	3759.5 (2523.3, 5324.2)	168.9 (112.9, 238.7)	2918.0 (2153.8, 3812.8)	63.1 (46.4, 83.8)	8606.5 (5725.4, 12264.1)	170.4 (113.5, 243.5)	138 (78.8, 216.2)	128.9 (111.6, 145.1)	52.2 (15.0, 99.1)	0.9 (-4.4, 6.7)
Croatia	2206.4 (1505.6, 2487.0)	51.3 (34.0, 58.4)	17896.4 (12190.3, 24795.1)	317.4 (217.0, 440.1)	1467.2 (1075.7, 2052.4)	36.2 (26.1, 51.0)	19070.3 (13016.0, 26259.3)	351.2 (238.4, 485.5)	-33.5 (-51.3, 3.5)	6.6 (1.4, 12.3)	-29.4 (-49.1, 12.7)	10.6 (5.6, 16.1)
Cuba	7875.3 (4838.5, 8900.0)	79.3 (47.6, 90.4)	16626.3 (11071.8, 23303.9)	158.8 (106.1, 224.1)	8242.1 (6191.7, 13774.2)	71.4 (54.6, 110.8)	23298.5 (15641.0, 32942.1)	148.8 (99.2, 211.2)	4.7 (-25.2, 129.0)	40.1 (30.0, 50.5)	-9.9 (-34.5, 85.9)	-6.3 (-11.4, -0.7)
Cyprus	889.9 (723.6, 1112.5)	118.2 (95.8, 148.1)	2147.0 (1448.6, 2982.9)	263.6 (178.8, 366.5)	1356.8 (891.9, 1653.7)	86.8 (60.9, 105.6)	4322.7 (2939.6, 5994.6)	255.0 (172.9, 352.8)	52.5 (1.9, 100.4)	101.3 (90.0, 113.3)	-26.6 (-49.1, -4.5)	-3.3 (-8.4, 2.5)
Czechia	5669.6 (3968.5, 6400.1)	63.0 (42.3, 72.7)	31135.7 (21050.2, 43335.9)	271.7 (183.6, 383.1)	6990.3 (4250.8, 8851.5)	57.1 (38.6, 73.4)	43530.9 (29064.2, 61893.2)	304.0 (204.9, 428.5)	23.3 (-8.0, 54.1)	39.8 (24,.0 61.0)	-9.4 (-29.7, 16.1)	11.9 (1.4, 26.4)
Côte d'Ivoire	6460.9 (3921.4, 8984.7)	55.8 (34.0, 72.7)	19713.7 (13272.7, 27581.1)	228.7 (154.2, 317.0)	11340.7 (7197.0, 15991.6)	50.3 (33.9, 68.7)	45342.2 (30488.4, 63901.3)	225.6 (152.2, 315.8)	75.5 (18.6, 155.4)	130.0 (113.6, 146.5)	-9.9 (-38.6, 28.0)	-1.4 (-6.6, 4.0)
Democratic People's Republic of Korea	16646.7 (10789.5, 26254.9)	66.3 (46.2, 99.9)	40254.5 (26681.4, 57357.9)	202.2 (135.0, 287.5)	9860.7 (6633.9, 14216.1)	38.2 (25.2, 56.3)	54211.1 (35606.6, 77020.4)	177.0 (118.4, 249.7)	-40.8 (-61.9, -2.7)	34.7 (25.5, 44.2)	-42.4 (-62.4, -8.0)	-12.5 (-18.0, -7.2)
Democratic Republic of the Congo	54181 (17468.3, 104544.3)	100.3 (39.5, 162.7)	63064.4 (42957.1, 87996.3)	226.2 (153.4, 315.4)	50889.5 (22527.4, 85172.8)	64.7 (26.7, 114.0)	138965.0 (94546.2, 195341.4)	216.4 (147.2, 302.4)	-6.1 (-52.2, 108.1)	120.4 (106.6, 135.1)	-35.5 (-63.0, 13.3)	-4.3 (-9.3, 0.5)

Denmark	5124.8 (4148.7, 6307.7)	93.5 (77.2, 114.6)	22373.9 (15266.6, 31215.0)	365.2 (247.6, 510.9)	7416.8 (5775.8, 9279.5)	100.8 (78.3, 125.8)	27332.3 (18553.2, 37862.8)	361.2 (244.0, 499.2)	44.7 (25.2, 64.0)	22.2 (16.3, 28.0)	7.8 (-9.7, 27.7)	-1.1 (-5.3, 3.2)
Djibouti	155.0 (88.2, 245.6)	35.7 (20.8, 54.1)	790.1 (531.3, 1113.2)	240.7 (163.0, 333.0)	455.9 (254.6, 748.7)	45.8 (26.1, 72.8)	2248.1 (1499.2, 3132.9)	222.6 (149.2, 308.6)	194.2 (98.4, 335.4)	184.6 (160.9, 207.4)	28.3 (-6.5, 77.9)	-7.5 (-12.4, -2.8)
Dominica	228.8 (158.5, 275.1)	314.5 (217.4, 380.3)	109.4 (73.9, 155.0)	163.6 (110.0, 231.3)	221.1 (162.7, 321.8)	336.6 (242.0, 490.5)	123.3 (83.5, 174.2)	153.7 (104.1, 215.4)	-3.3 (-32.9, 62.0)	12.7 (5.9, 20.3)	7.0 (-27.6, 80.8)	-6.0 (-10.9, -0.8)
Dominican Republic	27778.8 (7435.2, 42507.7)	298.4 (93.5, 442.1)	8366.3 (5675.1, 11691.4)	162.1 (110.2, 224.9)	23562.4 (10950.7, 38513.8)	223.1 (103.8, 363.1)	15846.4 (10695.8, 22294.6)	156.3 (105.4, 220.1)	-15.2 (-45.2, 65.8)	89.4 (76.3, 103.0)	-25.2 (-50.8, 29.8)	-3.6 (-9.2, 2.1)
Ecuador	13213.8 (8506.5, 16052.1)	128.6 (84.0, 152.7)	10757.5 (7360.3, 15071.3)	150.2 (102.6, 207.3)	14772 (11328.1, 19029.4)	90.7 (69.6, 116.5)	29743.1 (20353.7, 41141.0)	182.4 (124.7, 252)	11.8 (-18.5, 71.9)	176.5 (158.6, 193.4)	-29.4 (-47.9, 8.6)	21.5 (15.2, 27.9)
Egypt	92752.5 (31723.8, 171841.3)	133.8 (51.9, 231.7)	116376.2 (77543.6, 165965.2)	248.2 (165.5, 349.7)	101503.2 (51044.6, 166431.2)	116.1 (58.5, 189.4)	217050.5 (144479.2, 308885.5)	240.5 (160.8, 338.6)	9.4 (-40.6, 107.8)	86.5 (74.7, 98.0)	-13.3 (-50.6, 44.8)	-3.1 (-8.6, 2.4)
El Salvador	13008.9 (2788.0, 20079.9)	196.2 (50.9, 292.1)	6271.2 (4175.2, 8856.6)	166.5 (111.2, 234.7)	2781.0 (1945.4, 4228.3)	46.4 (32.2, 70.3)	9901.2 (6635.5, 13981.7)	167.5 (112.4, 236.4)	-78.6 (-89.0, 19.9)	57.9 (46.0, 69.4)	-76.4 (-87.5, 7.9)	0.6 (-5.5, 6.7)
Equatorial Guinea	519.1 (180.2, 933.3)	90.9 (36.7, 137.5)	724.0 (493.9, 1007.2)	227.4 (154.3, 312.9)	856.9 (334.4, 1485.6)	78.8 (30.5, 139.6)	2430.8 (1628.1, 3421.5)	250.3 (167.0, 348.8)	65.1 (-15.6, 234.7)	235.7 (202.0, 271.4)	-13.3 (-49.8, 45.7)	10.1 (0.6, 20.4)

Eritrea	789.4 (464.7, 1208.1)	31.5 (19.7, 49.3)	4872.7 (3295.5, 6916.8)	234.0 (159.4, 326.0)	1839.4 (898.6, 3363.6)	38.1 (20.2, 64.8)	11037.8 (7450.5, 15466.3)	222.3 (150.3, 308.9)	133.0 (39.8, 284.0)	126.5 (112.1, 141.5)	20.9 (-17.2, 78.1)	-5.0 (-9.8, 0.1)
Estonia	650.8 (517.3, 807.9)	43.1 (34.2, 54.0)	2514.1 (1707.3, 3534.0)	146.1 (98.4, 207.7)	662.6 (492.5, 887.4)	43.1 (33.0, 63.3)	2306.0 (1548.2, 3240.6)	142.8 (95.8, 202.1)	1.8 (-21.9, 32.6)	-8.3 (-14.2, -1.4)	0.0 (-22.9, 32.3)	-2.2 (-8.1, 4.3)
Eswatini	1047.8 (556.5, 1465.8)	165.2 (92.2, 220.7)	1656.2 (1110.1, 2321.3)	313.6 (211.9, 441.6)	1904.8 (984.3, 3074.8)	207.2 (107.1, 329.6)	2701.4 (1808.1, 3790.7)	308.6 (208.0, 432.4)	81.8 (17.9, 172.2)	63.1 (52.2, 73.7)	25.4 (-20.5, 88.4)	-1.6 (-6.6, 3.6)
Ethiopia	26522.9 (17755.5, 36939.6)	52.9 (37.9, 70.4)	83863.2 (57047.0, 117959.0)	233.4 (158.7, 326.8)	26507.8 (20417.5, 35691.3)	33.6 (27.0, 45.1)	172640.4 (115292.8, 244333.7)	228.8 (152.9, 320.7)	-0.1 (-38.2, 85.7)	105.9 (97.9, 113.0)	-36.5 (-55.9, 4.1)	-2.0 (-4.9, 0.7)
Fiji	432.6 (311.2, 577.0)	78.8 (57.1, 103.9)	1036.8 (692.4, 1457.6)	170.3 (114.7, 236.7)	780.1 (565.9, 1046.8)	92.6 (67.6, 122.8)	1511.3 (1016.4, 2099.9)	167.5 (113.3, 231.3)	80.3 (20.3, 165.6)	45.8 (35.5, 56.9)	17.5 (-21.4, 72.4)	-1.6 (-6.9, 4.0)
Finland	2035.6 (1609.9, 2395.2)	43.2 (31.6, 48.4)	15923.1 (10932.7, 21823.8)	256.9 (175.9, 352.2)	2935.7 (2216.4, 3441.7)	49.8 (33.4, 58.6)	19721.8 (13606.6, 27186.8)	238.1 (163.1, 328.2)	44.2 (27.8, 66.1)	23.9 (19.2, 29.1)	15.2 (-3.2, 35.1)	-7.3 (-10.6, -3.7)
France	64686.1 (52235.5, 84240.1)	103.9 (86.4, 137.6)	198392.9 (133367.3, 279621.1)	297.7 (199.7, 414.0)	94484.2 (80319.8, 126767.6)	110.5 (95.4, 156.5)	255176.3 (170038.3, 360695.8)	293.5 (194.5, 412.8)	46.1 (31.9, 62.3)	28.6 (20.7, 36.5)	6.4 (-4.2, 22.3)	-1.4 (-6.4, 4.0)
Gabon	1171.6 (434.1, 1938.3)	103.8 (40.5, 158.7)	2101.5 (1420.6, 2945.2)	270.3 (182.4, 380.4)	1715.6 (741.9, 2630.0)	112.8 (48.7, 169.8)	3886.4 (2602.6, 5480.2)	260.1 (176.1, 366.9)	46.4 (-1.6, 129.1)	84.9 (71.7, 97.3)	8.7 (-20.2, 53.8)	-3.8 (-9.5, 2.0)

Gambia	414.3 (253.5, 583.3)	44.8 (27.0, 63.6)	1756.9 (1187.7, 2469.8)	234.7 (158.9, 327.2)	836.9 (580.2, 1152.5)	48.1 (34.2, 62.6)	4154.2 (2796.1, 5842.5)	237.1 (161.6, 331.4)	102.0 (33.0, 229.7)	136.5 (122.2, 152.4)	7.3 (-27.5, 63.3)	1.0 (-3.9, 6.4)
Georgia	751.1 (627.4, 1238.0)	14.3 (11.9, 23.5)	11610.9 (7820.9, 16401.5)	196.5 (132.8, 275.6)	2236.3 (775.0, 2822.3)	54.5 (22.2, 67.4)	9273.7 (6185.6, 13242.9)	203.8 (135.4, 286.2)	197.7 (-23.1, 312.6)	-20.1 (-26.1, -13.8)	281.4 (14.5, 424.0)	3.7 (-3.1, 12.2)
Germany	77773.2 (60965.0, 87896.8)	84.7 (67.4, 101.8)	309436.4 (207288.0, 433640.3)	312.4 (209.0, 435.1)	117226.7 (83461.4, 131904.0)	100.5 (75.6, 116.6)	423607.3 (288448.8, 594333.7)	349.7 (236.8, 489.6)	50.7 (25.6, 63.9)	36.9 (25.8, 48.1)	18.6 (0.3, 29.5)	12.0 (3.1, 20.4)
Ghana	3481.5 (2418.9, 5380.5)	28.6 (20.0, 40.8)	24219.6 (16467.7, 33917.2)	219.9 (149.5, 307.8)	9762.6 (6442.9, 14803.8)	39.2 (27.3, 56.9)	58415.9 (39345.3, 82375.8)	230.0 (155.3, 321.9)	180.4 (104.6, 276.6)	141.2 (127.2, 157.2)	37.1 (1.6, 83.2)	4.6 (-0.4, 10.6)
Greece	2701.5 (2340.3, 4479.5)	26.2 (22.1, 45.1)	35985.1 (23880.9, 51369.1)	288.4 (191.4, 406.7)	6259.9 (4345.3, 7000.3)	45.9 (34.3, 53.2)	40819 (27437.9, 57849.4)	283.5 (188.9, 397.1)	131.7 (18.2, 184.8)	13.4 (6.5, 20.1)	75.3 (-5.6, 118.5)	-1.7 (-6.3, 3.4)
Greenland	72.8 (55.7, 111.2)	150.1 (119.0, 217.1)	93.6 (62.9, 131.4)	191.5 (129.8, 268.9)	101.4 (69.2, 133.8)	159.3 (108.7, 213.1)	125.9 (82.9, 179.0)	190.6 (127.1, 267.7)	39.4 (-15.0, 110.1)	34.5 (22.4, 48.1)	6.1 (-31.6, 55.8)	-0.5 (-5.3, 4.7)
Grenada	275.0 (154.2, 337.4)	333.1 (189.3, 403.1)	121.2 (82.8, 169.3)	166 (112.5, 233.2)	265.2 (216.8, 392.9)	270 (218.7, 400.5)	175.2 (116.4, 247.8)	157.4 (106.2, 222.0)	-3.6 (-29.4, 100.8)	44.6 (33.8, 56.1)	-18.9 (-40.3, 67.8)	-5.2 (-10.5, 0.4)
Guam	101.1 (80.5, 142.9)	95.9 (76.3, 131.4)	217.7 (143.9, 305.0)	185.7 (123.9, 260.2)	231.2 (167.1, 297.0)	128.6 (92.8, 165.7)	326.0 (218.3, 462.4)	178.8 (119.7, 254.7)	128.8 (48.4, 218.9)	49.8 (37.5, 62.5)	34.1 (-11.5, 82.3)	-3.7 (-9.5, 2.3)

Guatemala	11715.2 (7262.1, 13862.6)	124.3 (83.7, 142.9)	9471.0 (6369.1, 13246.2)	171.4 (116.5, 242.1)	15365.6 (11218.7, 22719.2)	100.2 (74.3, 144.2)	22694.9 (15281.9, 31964.6)	168.0 (113.0, 236.4)	31.2 (-8.5, 160.8)	139.6 (123.8, 155.4)	-19.4 (-41.6, 34.4)	-2.0 (-7.0, 2.9)
Guinea	4577.8 (2558.8, 6525.3)	62.5 (37.5, 80.3)	10424.8 (7162.1, 14570.3)	218.1 (149.1, 302.1)	6328.0 (4224.0, 8638.4)	51.6 (35.8, 67.3)	20577.7 (13920.1, 29090.4)	218.5 (147.8, 304.5)	38.2 (-5.2, 107.0)	97.4 (84.3, 112.4)	-17.4 (-39.8, 15.5)	0.2 (-5.4, 6.0)
Guinea-Bissau	656.9 (393.8, 952.0)	69.2 (43.6, 92.8)	1605.2 (1090.4, 2265.6)	219.9 (148.6, 309.7)	752.1 (547.1, 1005.7)	51.2 (37.3, 67.0)	3213.0 (2167.8, 4530.4)	225.6 (153.3, 315.7)	14.5 (-21.6, 79.8)	100.2 (86.3, 116.2)	-26.0 (-47.2, 9.6)	2.6 (-2.7, 8.7)
Guyana	2461.0 (1434.2, 2999.2)	342.7 (207.9, 411.3)	1041.6 (710.3, 1458.3)	181.4 (124.4, 251.2)	2393.0 (1725.7, 3422.3)	332.4 (240.1, 474.4)	1198.0 (816.3, 1664.5)	167.0 (113.6, 232.4)	-2.8 (-33.7, 80.4)	15.0 (6.1, 24.2)	-3.0 (-33.2, 79.2)	-7.9 (-13.2, -2.3)
Haiti	24437.4 (12655.3, 41001.7)	306.1 (192.7, 453.1)	8531.8 (5819.0, 11989.3)	178.2 (121.6, 248.8)	29715.4 (19732.8, 45534.8)	238.8 (168.4, 344.7)	17420.9 (11882.6, 24491.1)	174.1 (118.8, 242.4)	21.6 (-27.1, 108.5)	104.2 (91.7, 116.8)	-22.0 (-49.6, 16.2)	-2.3 (-7.2, 2.6)
Honduras	10542.4 (5489.6, 14839.6)	166.6 (105.0, 215.8)	5294.6 (3552.9, 7494.0)	168.7 (113.4, 239.6)	11702.4 (7957.4, 16229.4)	132.6 (94.1, 178.3)	12312.8 (8266.1, 17345.0)	163.8 (110.4, 233.0)	11.0 (-27.5, 80.5)	132.6 (115.9, 148.5)	-20.4 (-44.8, 12.3)	-2.9 (-7.5, 1.9)
Hungary	7210.2 (6105.8, 9117.9)	76.8 (63.9, 95.9)	32240.6 (21648.8, 45208.8)	272.1 (183.7, 383.2)	9103.5 (6319.4, 11478.1)	83.3 (60.6, 107.4)	31935.6 (21424.2, 44734.3)	260.2 (175.2, 368.2)	26.3 (-17.9, 61.0)	-0.9 (-7.2, 5.6)	8.5 (-20.0, 37.8)	-4.4 (-10.1, 2.1)
Iceland	109.3 (91.0, 138.9)	42.5 (35.4, 54.6)	739.2 (492.1, 1042.1)	283.0 (187.7, 400.6)	189.3 (147.4, 230.9)	46.4 (36.1, 59.0)	1224.0 (817.2, 1712.0)	283.2 (189.7, 393.9)	73.2 (42.0, 104.5)	65.6 (53.4, 78.9)	9.0 (-10.5, 31.6)	0.1 (-6.1, 7.4)

India	329124.7 (234369.2, 434258.7)	34.9 (26.1, 44.1)	1831392.7 (1239944.4, 2516742.6)	265.9 (181.2, 365.9)	368505.7 (302951.5, 460360.4)	29.7 (24.4, 36.9)	3664488.4 (2495864.7, 5062753.8)	277.1 (188.6, 383.2)	12.0 (-17.3, 65.6)	100.1 (93.9, 106.6)	-14.7 (-34.5, 19.0)	4.2 (2.5, 5.8)
Indonesia	73257.5 (41382.8, 102828.1)	38.8 (25.2, 51.4)	202173.4 (135701.0, 284147.2)	133.5 (90.1, 185.0)	76670.1 (56626.1, 92581.1)	34.0 (24.8, 40.9)	235121.4 (155929.8, 327420.8)	85.8 (57.1, 118.5)	4.7 (-25.4, 59.6)	16.3 (8.4, 24.2)	-12.4 (-33.8, 19.1)	-35.7 (-39.3, -32.0)
Iran (Islamic Republic of)	54660.1 (39128.6, 91566.7)	81.0 (63.8, 124.1)	125727.5 (83290.1, 179755.9)	264.4 (176.6, 371.4)	60065.2 (36035.3, 72218.8)	81.0 (47.9, 98.5)	224526.0 (147565.3, 316299.7)	262.5 (174.3, 371.5)	9.9 (-55.3, 72.0)	78.6 (65.9, 92.1)	0.0 (-55.5, 43.3)	-0.7 (-3.0, 1.4)
Iraq	11394.9 (7180.3, 16683.3)	50.6 (34.5, 68.5)	35377.5 (23495.4, 50441.1)	256.7 (171.8, 360.5)	12451.1 (8609.8, 16200.2)	32.0 (23.1, 40.6)	87219.0 (57800.8, 123748.9)	240.7 (160.8, 337.8)	9.3 (-29.3, 89.8)	146.5 (127.9, 166.7)	-36.9 (-54.6, -0.6)	-6.2 (-11.1, -1.2)
Ireland	3220.5 (2593.0, 4037.3)	90.1 (72.5, 113.5)	10696.2 (7171.4, 14966.1)	288 (191.7, 404.5)	4763.2 (4056.2, 6421.5)	89.8 (75.5, 119.1)	18098.2 (12065.7, 25403.0)	303.8 (202.2, 425.7)	47.9 (29.5, 74.6)	69.2 (59.1, 79.9)	-0.4 (-13.6, 16.0)	5.5 (-0.1, 11.5)
Israel	5616.4 (4681.6, 7128.4)	114.8 (95.4, 144.6)	13977.4 (9351.0, 19599.5)	292.7 (194.6, 414.4)	13614.3 (10697.1, 16417.3)	129.1 (105.1, 158.3)	28680.0 (19343.6, 40668.1)	290.1 (195.9, 410.4)	142.4 (85.7, 174.1)	105.2 (92.7, 118.1)	12.4 (-7.8, 26.8)	-0.9 (-6.3, 5.0)
Italy	41683.7 (36333.4, 57035.7)	75.8 (64.1, 99.1)	271387.0 (182274.4, 382226.0)	389.3 (259.9, 547.3)	77254.8 (49422.1, 84652.2)	94.6 (63.9, 103.8)	304735.1 (206239.3, 427191.9)	367.5 (245.7, 511.4)	85.3 (6.7, 107.1)	12.3 (7.3, 17.6)	24.8 (-21.7, 38.8)	-5.6 (-9.7, -0.9)
Jamaica	1897.4 (1404.7, 2243.0)	83.4 (62.5, 98.5)	3140.0 (2132.9, 4398.7)	162.8 (109.6, 230.2)	3508.0 (2596.1, 4830.4)	127.7 (92.8, 179.2)	4534.7 (3063.9, 6396.1)	155.5 (105.7, 218.8)	84.9 (34.9, 159.7)	44.4 (34.8, 53.9)	53.2 (11.1, 117.2)	-4.5 (-10.0, 1.2)

Japan	48531.4 (42278.6, 67968.8)	41.0 (35.9, 56.9)	453276.5 (301401.8, 636579.5)	307.6 (204.8, 435.8)	63194.6 (51214.7, 82301.0)	34.8 (30.2, 52.6)	445408.1 (299250.6, 622603.1)	272.5 (183.2, 376.8)	30.2 (1.1, 41.0)	-1.7 (-9.6, 5.9)	-15.1 (-23.8, -0.7)	-11.4 (-16.6, -6.3)
Jordan	3816.5 (2816.0, 5044.2)	94.3 (72.8, 116.0)	7290.7 (4811.1, 10471.8)	253.5 (168.3, 356.0)	8748.4 (6365.2, 11435.4)	83.0 (62.3, 108.4)	24884.1 (16295.6, 35500.8)	240.2 (158.9, 336.5)	129.2 (66.2, 215.0)	241.3 (213.4, 268.5)	-11.9 (-33.7, 17.5)	-5.2 (-11.0, 0.3)
Kazakhstan	4326.4 (3638.1, 7239.8)	26.4 (22.3, 44.0)	31770.8 (21423.1, 44355.1)	208.8 (141.0, 292.1)	12226.7 (9277.1, 15426.7)	66.5 (50.8, 84.6)	38316.6 (25553.0, 53924.9)	199.9 (134.0, 281.2)	182.6 (61.3, 255.6)	20.6 (13.0, 29.4)	151.6 (44.9, 215.2)	-4.3 (-9.4, 1.8)
Kenya	4232.9 (3057.6, 5322.1)	22.8 (16.6, 30.5)	36334.6 (24325.3, 50747.6)	257.8 (172.4, 360.3)	9972.1 (6946.6, 14090.9)	29.8 (21.2, 42.2)	88375.8 (59065.1, 123548.1)	238.9 (160.9, 332.2)	135.6 (62.7, 215.3)	143.2 (136.9, 148.9)	30.9 (4.9, 65.9)	-7.3 (-9.4, -4.9)
Kiribati	112.1 (73.0, 155.1)	203.1 (142.7, 266.1)	105.7 (71.1, 148.2)	180.7 (122.0, 253.1)	178.4 (116.1, 243.2)	195.7 (130.4, 263.2)	178.9 (119.3, 251.4)	175.5 (118.5, 246.3)	59.2 (18.7, 113.9)	69.2 (57.9, 80.7)	-3.6 (-26.7, 25.3)	-2.9 (-8.5, 2.9)
Kuwait	1131.7 (827.1, 1357.6)	66.4 (49.1, 78.5)	4033.5 (2645.7, 5751.5)	278.3 (184.7, 392.4)	2128.5 (1654.8, 2970.9)	65.0 (50.6, 91.2)	12350.1 (7985.9, 17586.9)	277.2 (184.8, 390.8)	88.1 (43.0, 160.6)	206.2 (179.5, 233.4)	-2.0 (-23.9, 31.0)	-0.4 (-5.4, 4.8)
Kyrgyzstan	1140.3 (888.0, 1378.6)	23.7 (18.9, 28.8)	7400.0 (4991.9, 10324.6)	194.2 (131.4, 270.8)	1995.2 (1591.9, 2614.0)	30.8 (24.6, 39.7)	11114 (7508.3, 15757.0)	183.1 (123.8, 257.6)	75.0 (45.4, 117.2)	50.2 (40.1, 60.1)	30.1 (9.8, 56.5)	-5.7 (-11.3, -0.3)
Lao People's Democratic Republic	4565.5 (1849.8, 8418.4)	97.1 (50.1, 154.9)	4384.4 (2947.0, 6086.6)	141.4 (95.3, 196.5)	4017.0 (2645.9, 5804.0)	63.7 (44.9, 88.0)	8141.2 (5362.6, 11400.7)	129.5 (85.6, 180.8)	-12.0 (-49.2, 93.7)	85.7 (71.0, 101.4)	-34.3 (-57.6, 15.7)	-8.4 (-14.5, -2.3)

Latvia	732.3 (598.5, 971.0)	28.0 (23.0, 37.9)	4522.8 (3080.1, 6311.6)	153.0 (103.3, 215.2)	938.7 (635.5, 1217.8)	41.7 (30.7, 55.3)	4571.2 (3105.8, 6355.6)	183.6 (124.4, 255.3)	28.2 (-14.7, 63.8)	1.1 (-6.4, 8.2)	49.0 (10.9, 88.5)	20.0 (12.2, 27.1)
Lebanon	2578.3 (1814.6, 3557.9)	70.8 (51.7, 95.9)	7467.8 (4916.7, 10666.7)	254.8 (170.0, 361.0)	3772.4 (2457.5, 5300.9)	73.2 (47.7, 103.0)	12759.9 (8474.5, 17982.7)	244.0 (161.7, 343.7)	46.3 (-8.2, 123.0)	70.9 (58.6, 83.5)	3.3 (-33.5, 51.1)	-4.2 (-10.2, 1.9)
Lesotho	1549.2 (848.8, 2499.2)	101.8 (55.9, 169.5)	4054.0 (2755.1, 5705.6)	296.0 (198.8, 418.8)	3225.0 (1647.1, 4743.5)	187.5 (97.6, 274.1)	5106.3 (3423.6, 7203.4)	293.5 (198.7, 411.0)	108.2 (27.0, 221.1)	26.0 (19.0, 33.1)	84.1 (9.7, 184.6)	-0.9 (-5.5, 3.9)
Liberia	1851.0 (961.0, 2992.7)	82.7 (47.0, 119.3)	3311.8 (2266.9, 4619.0)	213.2 (145.5, 296.8)	2130.5 (1349.4, 3130.0)	56.5 (36.2, 81.0)	7386.0 (4954.1, 10495.4)	202.5 (135.6, 283.1)	15.1 (-26.1, 95.9)	123.0 (105.0, 142.2)	-31.7 (-52.1, 4.7)	-5.0 (-10.2, 0.6)
Libya	4346.4 (2567.3, 6687.5)	87.6 (55.7, 125.8)	8989.0 (5948.2, 12843.8)	267.8 (180.1, 374.3)	5321.4 (3496.6, 7618.2)	95.7 (63.7, 135.5)	16066.1 (10558.0, 22796.6)	240.9 (159.5, 337.7)	22.4 (-27.4, 88.7)	78.7 (60.6, 99.1)	9.3 (-32.3, 63.3)	-10.0 (-15.5, -3.4)
Lithuania	921.7 (805.6, 1310.6)	26.8 (23.1, 37.1)	9717.1 (6528.8, 13518.6)	240.8 (162.4, 336.7)	1064.6 (791.8, 1331.3)	38.0 (27.7, 47.5)	8830.7 (5950.5, 12293.9)	240.9 (162.0, 336.5)	15.5 (-14.6, 42.2)	-9.1 (-15.0, -2.8)	42.0 (6.2, 76.7)	0.1 (-5.5, 6.0)
Luxembourg	366.0 (301.2, 459.3)	91.7 (76.0, 116.0)	1273.5 (863.1, 1774.4)	274.3 (185.0, 381.5)	617.2 (486.1, 782.6)	84.3 (66.7, 114.3)	2317.3 (1559.7, 3208.2)	288.5 (193.8, 397.8)	68.6 (35.5, 102.7)	82.0 (72.3, 91.8)	-8.1 (-26.3, 15.1)	5.2 (0.0, 10.4)
Madagascar	4309.8 (2570.1, 5799.0)	35.2 (21.3, 47.9)	19080.2 (12849.9, 26453.0)	224.8 (151.5, 314.2)	6070.0 (3518.1, 9414.4)	29.8 (16.9, 44.7)	41735.3 (27681.1, 58992.7)	214.3 (144.5, 300.7)	40.8 (-4.5, 109.1)	118.7 (104.1, 134.4)	-15.4 (-37.8, 15.0)	-4.7 (-10.2, 1.3)

Malawi	3631.9 (2308.2, 5453.9)	35.0 (25.3, 48.0)	15312 (10349.1, 21460.5)	224.7 (151.4, 312.9)	4521.7 (3225.4, 6234.7)	32.7 (24.5, 43.3)	28860.2 (19344.8, 40806.5)	219.5 (148.4, 305.5)	24.5 (-26.1, 126.1)	88.5 (76.3, 101.1)	-6.7 (-36.7, 39.5)	-2.3 (-7.0, 2.8)
Malaysia	7155.6 (5855.0, 9056.7)	48.0 (40.4, 60.4)	21822.1 (14501.8, 30377.4)	151.8 (100.7, 211.1)	16805.3 (11769.3, 22081.8)	58.0 (40.8, 76.0)	43240.6 (28291.7, 61094.5)	136.2 (89.3, 191.1)	134.9 (76.7, 216.6)	98.2 (83.5, 114.1)	20.9 (-9.4, 62.0)	-10.3 (-16.0, -4.3)
Maldives	659.0 (368.9, 1023.4)	269.2 (174.3, 379.2)	254.4 (170.6, 354.5)	151.0 (100.9, 209.1)	843.7 (655.1, 1085.5)	205.4 (160.6, 265.4)	588.3 (386.3, 841.0)	117.4 (78.1, 166.5)	28.0 (-17.8, 136.1)	131.2 (100.2, 161.4)	-23.7 (-46.2, 17.4)	-22.3 (-28.5, -15.8)
Mali	5516.7 (3304.3, 8332.2)	56.3 (34.6, 74.8)	15763.1 (10737.4, 22144.6)	231.1 (158.5, 321.9)	11656.1 (7530.9, 17374.3)	50.4 (35.2, 68.9)	40829.6 (27523.8, 57914.4)	240.4 (161.8, 336.3)	111.3 (43.4, 215.7)	159.0 (138.6, 179.4)	-10.4 (-34.6, 28.3)	4.0 (-2.5, 9.9)
Malta	159.5 (129.8, 201.3)	43.6 (35.1, 55.4)	1019.6 (679.8, 1435.2)	249.5 (166.1, 350.4)	270.3 (215.7, 344.2)	51.7 (40.6, 68.5)	1728.1 (1165.3, 2416.9)	265.5 (179.6, 370.8)	69.5 (43.8, 101.3)	69.5 (56.9, 82.7)	18.5 (-3.5, 44.9)	6.4 (0.8, 12.6)
Marshall Islands	31.1 (21.9, 43.0)	113.8 (77.0, 162.9)	52.4 (35.2, 73.5)	164.2 (111.0, 229.2)	58.6 (39.9, 82.2)	123.2 (85.0, 171.6)	80.2 (53.1, 112.6)	157.1 (104.5, 219.4)	88.3 (37.8, 156.5)	53.0 (39.7, 66.1)	8.3 (-19.9, 51.3)	-4.3 (-9.9, 1.4)
Mauritania	1289.7 (833.2, 1722.3)	74.1 (45.3, 97.7)	3850.2 (2591.3, 5369.9)	239.3 (160.7, 332.8)	1515.5 (1000.0, 2222.8)	48.3 (32.7, 69.2)	7283.8 (4918.6, 10247.7)	228.0 (154.0, 320.5)	17.5 (-23.9, 85.3)	89.2 (72.3, 103.1)	-34.9 (-54.8, 2.7)	-4.7 (-11.1, 1.2)
Mauritius	724.6 (415.2, 823.9)	77.3 (43.2, 87.5)	1352.1 (904.9, 1902.2)	138.6 (92.4, 193.1)	727.6 (537.9, 1238.8)	53.4 (39.2, 93.4)	2087.0 (1386.1, 2990.6)	131.3 (86.9, 185.8)	0.4 (-30.0, 150.5)	54.4 (40.1, 70.1)	-30.9 (-52.0, 73.4)	-5.3 (-11.8, 1.1)

Mexico	63764.1 (56335.3, 91399.2)	89.8 (78.3, 118.5)	96525.5 (64722.1, 136069.0)	157.8 (107.1, 220.6)	181158.7 (129317.2, 214476.4)	150.7 (109.2, 178.5)	223899.4 (151577.9, 313506.7)	182.4 (123.2, 256.0)	184.1 (84.4, 249.2)	132 (119.4, 144.0)	67.9 (23.9, 98.3)	15.6 (12.8, 18.1)
Micronesia (Federated States of)	114.1 (80.9, 148.1)	165.5 (118.3, 215.2)	126.4 (85.4, 175.7)	165.7 (111.6, 230.4)	137.7 (78.1, 191.6)	161.9 (95.8, 224.4)	148.0 (99.9, 207.7)	158.2 (107.2, 223.0)	20.8 (-26.4, 67.8)	17.0 (7.8, 26.9)	-2.2 (-39.2, 40.4)	-4.6 (-10.2, 0.9)
Monaco	13.1 (9.6, 19.5)	39.3 (29.0, 57.8)	148.7 (98.3, 209.9)	342.3 (225.2, 479.4)	23.0 (14.9, 29.7)	42.4 (29.0, 55.1)	187.6 (124.5, 267.0)	337.6 (225.0, 475.6)	75.5 (2.2, 165.4)	26.2 (19.1, 34.0)	7.8 (-32.2, 57.3)	-1.4 (-6.7, 4.1)
Mongolia	3677.1 (2213.4, 5138.9)	146.2 (90.4, 193.0)	3186.8 (2176.9, 4494.0)	190.0 (130.4, 264.2)	2940.6 (2148.3, 3978.7)	84.4 (62.8, 114.9)	6114.1 (4051.7, 8670.8)	185.4 (123.7, 262.9)	-20.0 (-49.9, 40.9)	91.9 (75.5, 109.7)	-42.2 (-61.5, -2.0)	-2.4 (-8.8, 3.8)
Montenegro	169.0 (139.1, 210.7)	29.4 (23.9, 37.1)	1714.1 (1153.0, 2432.4)	269.8 (181.2, 382.7)	138.2 (105.3, 174.2)	21.6 (16.2, 28.4)	1844.1 (1226.4, 2586.3)	249.4 (166.1, 350.4)	-18.3 (-34.1, 4.1)	7.6 (-0.1, 15.7)	-26.6 (-42.8, -3.2)	-7.6 (-13.0, -1.0)
Morocco	24143.2 (12693.4, 39249.9)	82.9 (46.7, 124.6)	52976.3 (35327.2, 74966.2)	250.1 (165.2, 353.3)	30383.3 (19390.6, 42710.3)	95.8 (61.1, 134.7)	86945 (58037.7, 122054.0)	241.3 (159.7, 339.0)	25.8 (-31.2, 115.7)	64.1 (51.3, 76.9)	15.5 (-34.1, 77.5)	-3.5 (-9.0, 2.1)
Mozambique	5763.8 (3686.2, 10380.7)	43.3 (30.4, 71.1)	21748.5 (14720.0, 30600.0)	223.2 (151.3, 312.9)	12131.6 (7473.6, 20623.6)	50.5 (33.8, 83.4)	44374.1 (30046.0, 62298.5)	216.6 (145.8, 304.7)	110.5 (26.3, 262.4)	104.0 (92.0, 116.6)	16.8 (-16.4, 64.5)	-3.0 (-7.6, 1.7)
Myanmar	38183.8 (18701.6, 74713.2)	89.5 (52.8, 155.0)	49176.4 (32856.9, 69230.9)	146.4 (98.2, 204.3)	35326.4 (24581.2, 51604.5)	70.6 (49.7, 101.8)	78827.5 (52699.4, 110611.1)	143.3 (96.1, 200.7)	-7.5 (-47.7, 82.4)	60.3 (47.0, 74.6)	-21.1 (-50.3, 28.2)	-2.2 (-8.6, 5.1)

Namibia	1037.4 (487.0, 1642.6)	93.1 (44.0, 147.8)	3411.4 (2269.1, 4773.9)	325.8 (218.1, 453.0)	1986.6 (917.7, 3647.7)	100.9 (47.5, 181.6)	6209.8 (4136.0, 8657.1)	319.1 (215.1, 442.8)	91.5 (23.6, 190.5)	82.0 (70.7, 94.8)	8.4 (-27.4, 58.3)	-2.0 (-6.9, 3.0)
Nauru	11.1 (7.5, 15.0)	146.0 (102.2, 192.9)	13.7 (9.2, 19.3)	178.7 (120.0, 249.2)	11.7 (7.6, 16.6)	146.8 (100.1, 200.5)	13.7 (9.1, 19.3)	166.4 (110.9, 232.5)	5.4 (-18.4, 34.3)	0.0 (-7.1, 7.5)	0.5 (-21.5, 27.6)	-6.9 (-12.3, -1.0)
Nepal	9413.3 (5185.7, 14908.7)	36.7 (23.3, 53.1)	25833.7 (17617.1, 35789.0)	178.3 (122.3, 246.1)	11386.4 (7989.9, 15352.2)	41.4 (29.4, 55.3)	42866.9 (29241.4, 59553.4)	158.3 (108.0, 219.1)	21.0 (-23.7, 92.5)	65.9 (54.7, 78.5)	13.0 (-20.8, 60.8)	-11.3 (-17.0, -5.1)
Netherlands	15949.1 (11138.3, 17514.9)	110.9 (73.9, 123.2)	50948.0 (33902.5, 71760.7)	298.6 (197.9, 421.9)	16145.5 (13968.6, 23730.0)	81.3 (68.1, 113.7)	68771.8 (45333.5, 98338.8)	297.8 (196.9, 418.3)	1.2 (-14.4, 64.8)	35.0 (25.8, 45.4)	-26.7 (-39.1, 19.3)	-0.3 (-5.4, 5.2)
New Zealand	3496.7 (2685.2, 4054.7)	99.9 (76.5, 115.0)	6866.2 (4641.7, 9463.6)	190.7 (128.6, 265.1)	7352.2 (5853.4, 9360.1)	135.7 (108.2, 173.3)	10828.5 (7364.0, 15027.5)	193.7 (131.0, 267.5)	110.3 (92.3, 146.3)	57.7 (49.1, 66.9)	35.9 (24.6, 58.4)	1.5 (-3.5, 6.9)
Nicaragua	3015.2 (1977.7, 3908.2)	67.0 (49.2, 80.6)	3944.7 (2641.4, 5628.0)	166.1 (110.7, 237.0)	3202.8 (2460.1, 4137.1)	56.1 (43.9, 72.5)	8360.7 (5603.2, 11865.4)	158.5 (106.8, 225.9)	6.2 (-20.9, 59.4)	111.9 (94.7, 129.1)	-16.3 (-35.1, 10.9)	-4.6 (-9.2, 0.3)
Niger	4859.2 (2624.9, 7355.6)	52.7 (32.8, 68.8)	13058.9 (8840.6, 18433.1)	225.1 (151.3, 316.1)	9619.5 (6114.0, 14199.9)	40.3 (27.1, 57.9)	36006.2 (24440.3, 51230.3)	220.5 (149.2, 309.3)	98.0 (29.2, 227.6)	175.7 (154.2, 197.7)	-23.5 (-46.0, 11.0)	-2.0 (-7.4, 3.5)
Nigeria	45879.6 (28041.9, 62635.8)	47.9 (29.9, 61.4)	177700.3 (120490.2, 246580.0)	250.2 (170.3, 347.2)	84475.4 (57312.2, 117576.2)	42.8 (30.7, 57.4)	435663.8 (293116.2, 611741.1)	266.3 (180.6, 370.1)	84.1 (40.0, 159.1)	145.2 (136.3, 154.7)	-10.7 (-32.1, 21.8)	6.4 (4.0, 9.0)

Niue	2.4 (1.7, 3.3)	112.3 (78.5, 153.2)	3.7 (2.5, 5.2)	174.4 (118.6, 243.0)	2.1 (1.4, 2.9)	113.0 (76.0, 158.3)	3.0 (2.0, 4.3)	159.5 (106.3, 221.7)	-12.8 (-41.8, 31.4)	-18.2 (-24.0, -12.3)	0.6 (-34.1, 53.5)	-8.6 (-13.5, -3.4)
North Macedonia	1101.8 (622.0, 1451.9)	60.0 (33.1, 80.2)	5244.9 (3522.6, 7372.6)	259.1 (173.8, 364.9)	491.4 (374.6, 691.0)	24.2 (18.6, 34.2)	6163.6 (4153.7, 8717.5)	241.6 (163.1, 341.0)	-55.4 (-70.7, -10.8)	17.5 (9.1, 26.5)	-59.7 (-73.8, -18.4)	-6.8 (-12.4, -1.1)
Northern Mariana Islands	39.6 (25.8, 64.4)	120.6 (84.5, 187.7)	66.8 (43.7, 94.6)	171.8 (114.3, 241.1)	78.1 (56.9, 100.9)	152.6 (112.9, 196.0)	90.6 (59.5, 130.6)	166.1 (109.6, 234.5)	97.2 (19.1, 215.4)	35.5 (17.5, 55.6)	26.6 (-18.3, 87.2)	-3.3 (-8.9, 2.4)
Norway	2475.0 (2126.6, 3470.1)	57.5 (47.9, 76.4)	19538.3 (13157.1, 27162.3)	390.0 (260.6, 543.1)	4393.9 (3470.6, 5533.5)	70.9 (54.7, 88.0)	27957.9 (18751.8, 39060.2)	411.4 (274.0, 572.2)	77.5 (36.9, 93.3)	43.1 (37.0, 48.7)	23.3 (-0.6, 35.1)	5.5 (1.8, 8.8)
Oman	420.6 (295.2, 566.7)	21.2 (15.1, 27.9)	4262.8 (2841.2, 6130.5)	274.6 (183.6, 384.2)	714.9 (525.3, 870.9)	22.4 (17.0, 27.4)	9932.7 (6495.6, 14140.0)	253.5 (169.0, 356.7)	70.0 (25.0, 131.9)	133.0 (111.2, 157.8)	5.4 (-19.3, 38.4)	-7.7 (-12.5, -2.5)
Pakistan	61853.8 (27816.3, 113422.0)	41.0 (21.2, 74.2)	197371.2 (133720.2, 273020.9)	233.3 (158.5, 322.6)	128727.2 (62818.5, 225320.5)	53.0 (26.3, 92.3)	390650.2 (264704.9, 546397.6)	221.8 (149.9, 305.8)	108.1 (47.8, 217.6)	97.9 (88.0, 109.0)	29.4 (0.1, 70.8)	-4.9 (-8.9, -1.2)
Palau	11.9 (8.3, 16.4)	93.7 (66.4, 127.9)	22.9 (15.5, 32.1)	173.0 (117.0, 243.0)	23.7 (15.0, 35.0)	111.6 (72.6, 160.7)	34.9 (23.1, 49.6)	153.7 (104.5, 215.0)	100 (30.4, 191.6)	52.1 (37.8, 67.1)	19.1 (-21.9, 70.9)	-11.1 (-16.3, -5.9)
Palestine	4071.8 (1958.2, 6305.1)	121.4 (77.4, 170.1)	3734.2 (2462.2, 5322.8)	239.9 (159.4, 338.6)	3335.4 (2454.1, 4487.8)	68.3 (53.4, 90.5)	9404.3 (6247.9, 13479.0)	228.9 (152.4, 325.2)	-18.1 (-49.9, 72.5)	151.8 (134.9, 171.5)	-43.8 (-62.2, -9.5)	-4.6 (-9.5, 1.1)

Panama	2209.5 (1117.8, 2606.4)	94.2 (49.9, 110.4)	3309.4 (2222.7, 4642.1)	178.8 (120.4, 251.5)	3130.3 (2247.6, 4659.4)	76.4 (54.8, 114.7)	7502.8 (5062.0, 10653.3)	180.0 (121.3, 255.9)	41.7 (-4.0, 223.6)	126.7 (111.4, 144.0)	-19.0 (-45.1, 78.7)	0.7 (-4.8, 6.7)
Papua New Guinea	2649.8 (1898.6, 3556.3)	77.3 (53.2, 108.5)	5370.4 (3616.7, 7463.3)	169.0 (114.6, 234.1)	6816.7 (4498.6, 9708.7)	80.2 (52.2, 121.3)	12957.4 (8611.4, 18242.3)	159.3 (107.0, 222.2)	157.3 (85.8, 261.1)	141.3 (124.8, 159.8)	3.6 (-23.1, 38.4)	-5.8 (-11.2, 0.2)
Paraguay	3685.8 (2098.7, 5004.6)	78.2 (54.2, 98.0)	3473.6 (2332.4, 4963.7)	111.5 (75.1, 157.1)	6857.0 (3746.9, 9786.1)	111.4 (60.6, 158.4)	6540.5 (4410.6, 9261.8)	102.9 (69.5, 145.2)	86.0 (29.8, 159.4)	88.3 (73.2, 103.7)	42.4 (-4.8, 100.6)	-7.7 (-13.5, -1.3)
Peru	50250.3 (19496.0, 72363.3)	198.4 (82.9, 275.0)	33838.7 (22908.9, 47236.7)	208.2 (141.4, 290.9)	13833.9 (7337.2, 31476.5)	42.4 (22.4, 97.0)	62996.8 (42025.0, 88521.5)	188.9 (126.4, 266.3)	-72.5 (-88.6, 30.4)	86.2 (72.2, 101.6)	-78.6 (-90.9, -6.5)	-9.3 (-14.4, -3.7)
Philippines	45718.5 (34744.9, 62344.6)	80.7 (63.8, 95.6)	77140.1 (51464.4, 108102.5)	165.3 (110.3, 230.2)	67974.4 (54260.3, 87665.3)	69.5 (54.8, 85.0)	186152 (122794.5, 261541.0)	184.2 (122.1, 259.1)	48.7 (18.9, 88.3)	141.3 (128.1, 152.8)	-13.9 (-27.8, 2.3)	11.5 (7.3, 15.0)
Poland	26917.2 (18694.5, 29817.2)	76.1 (53.0, 85.7)	119901.3 (80067.6, 169646.8)	294.8 (196.9, 417.8)	14765.6 (11322.8, 27069.0)	37.7 (28.0, 75.1)	62322.6 (42517.3, 86135.5)	133.3 (89.9, 186.6)	-45.1 (-59.2, 19.7)	-48.0 (-52.9, -42.8)	-50.5 (-65.1, 17.7)	-54.8 (-59.0, -50.3)
Portugal	5816.3 (4992.1, 8049.3)	63.2 (52.4, 82.0)	29017.5 (19315.5, 40975.7)	247.0 (163.4, 346.0)	13714.8 (7585.7, 15664.2)	93.1 (56.8, 107.8)	29436.4 (19686.4, 41526.1)	199.2 (133.1, 277.9)	135.8 (6.3, 182.1)	1.4 (-4.6, 8.4)	47.5 (-22.9, 77.6)	-19.3 (-24.3, -14.4)
Puerto Rico	12700.0 (7177.7, 14351.2)	361.1 (205.8, 408.0)	6187.9 (4150.2, 8736.1)	172.2 (115.3, 244.0)	11493.6 (8585.3, 15270.3)	255.1 (188.7, 365.8)	8587.3 (5698.0, 12282.2)	166.5 (112.1, 234.0)	-9.5 (-34.8, 60.4)	38.8 (28.9, 49.2)	-29.3 (-50.8, 38.5)	-3.3 (-8.5, 2.2)

Qatar	483.8 (339.0, 704.1)	150.4 (113.6, 198.4)	984.4 (642.5, 1411.5)	268.7 (177.9, 378.3)	1378.5 (988.9, 2121.5)	108.9 (77.7, 145.5)	6561 (4249.8, 9385.7)	254.5 (168.6, 356.6)	184.9 (92.3, 345.5)	566.5 (513.9, 619.7)	-27.6 (-47.7, 4.5)	-5.3 (-10.9, 0.1)
Republic of Korea	21651.8 (17314.8, 29604.9)	59.2 (47.0, 78.2)	117546.3 (78377.8, 165710.4)	276.1 (184.0, 385.1)	14958.8 (11384.7, 20009.3)	26.4 (20.4, 36.5)	186400.6 (122183.3, 263889.5)	268.1 (177.2, 380.9)	-30.9 (-54.5, -4.8)	58.6 (42.6, 75)	-55.4 (-69.3, -36.5)	-2.9 (-8.4, 3.3)
Republic of Moldova	1306.7 (1122.5, 1763.2)	30.5 (26.1, 41.0)	6728.1 (4575.2, 9490.4)	149.4 (101.4, 210.7)	1429.2 (1168.3, 2086.8)	39.4 (32.1, 63.8)	6013.9 (4073.2, 8457.8)	138.8 (93.9, 196.1)	9.4 (-9.9, 36.4)	-10.6 (-17.5, -3.3)	29.0 (4.5, 71.7)	-7.1 (-13.0, -0.8)
Romania	10694.5 (5367.9, 12497.9)	54.3 (25.6, 65.0)	66498.5 (44484.2, 94715.6)	262.7 (176.3, 372.7)	3666.2 (2816.7, 6254.9)	19.4 (14.8, 34.8)	64136.6 (42670.3, 90616.9)	265.8 (178.7, 374.2)	-65.7 (-75.7, -5.9)	-3.6 (-10.7, 3.2)	-64.4 (-75.6, 14.2)	1.2 (-5.1, 7.4)
Russian Federation	77567.7 (47550.2, 84208.6)	55.6 (33.7, 60.3)	219887.8 (148741.1, 306533.4)	135.7 (91.9, 189.9)	60758.1 (50609.6, 91210.0)	43.6 (36.1, 64.6)	148529.3 (100839.5, 206658.4)	91.2 (61.9, 128.7)	-21.7 (-36.3, 49.8)	-32.5 (-39.7, -24.5)	-21.6 (-36.7, 49.5)	-32.8 (-40.2, -24.4)
Rwanda	3375.5 (1966.7, 4696.2)	53.1 (32.1, 69.2)	10347.1 (6891.0, 14537.6)	214.7 (143.7, 300.4)	4703.1 (2800.9, 6918.7)	48.3 (29.5, 67.1)	19778.4 (13313.2, 27821.3)	208.0 (140.0, 291.6)	39.3 (-16.7, 145.6)	91.1 (77.3, 105.3)	-9.1 (-40.2, 43.0)	-3.1 (-8.5, 2.5)
Saint Kitts and Nevis	144.1 (90.2, 165.5)	370.7 (229.3, 426.1)	60.9 (41.5, 85.4)	168.2 (114.2, 238.0)	131.6 (83.0, 232.6)	227.5 (145.9, 401.1)	109.8 (74.1, 156.3)	161.4 (109.3, 229.1)	-8.7 (-44.3, 107.2)	80.4 (63.0, 97.0)	-38.6 (-61.9, 38.9)	-4.0 (-9.7, 1.2)
Saint Lucia	321.4 (193.8, 379.2)	251.9 (153.9, 289.9)	180.9 (121.6, 254.9)	170.7 (115.2, 241.2)	331.8 (255.1, 539.9)	190.9 (143.1, 309.5)	326.2 (220.0, 462.6)	160.3 (108.9, 227.3)	3.2 (-25.1, 125.1)	80.3 (65.9, 94.7)	-24.2 (-45.8, 66.2)	-6.1 (-11.3, -1.0)

Saint Vincent and the Grenadines	361.2 (211.7, 438.8)	350.0 (213.0, 415.1)	139.6 (94.2, 197.1)	165.2 (112.4, 233.1)	382.2 (298.5, 561.6)	342.7 (263.3, 505.7)	202.7 (137.4, 288.8)	158.8 (107.6, 223.1)	5.8 (-25.3, 107.4)	45.2 (32.6, 56.6)	-2.1 (-30.4, 83.9)	-3.8 (-9.4, 2.3)
Samoa	161.4 (114.7, 220.0)	142.2 (102.8, 191.0)	192.7 (128.5, 272.1)	156.8 (104.7, 218.3)	194.2 (130.4, 266.8)	115.0 (78.0, 157.1)	270.2 (180.8, 380.3)	148.2 (98.4, 207.0)	20.3 (-18.3, 76.0)	40.2 (30.7, 50.1)	-19.1 (-44.2, 15.0)	-5.5 (-10.8, 0.0)
San Marino	10.3 (8.1, 14.4)	44.0 (34.0, 62.6)	85.4 (57.0, 119.9)	311.9 (207.9, 436.8)	20.8 (12.9, 30.9)	49.6 (31.6, 71.7)	131.8 (86.9, 184.9)	305.6 (201.9, 430.1)	102.3 (18.7, 229.5)	54.2 (45.5, 64.1)	12.7 (-32.2, 80.4)	-2.0 (-7.6, 3.4)
Sao Tome and Principe	106.3 (79.0, 147.0)	90.6 (67.7, 115.6)	207.9 (140.0, 295.5)	228.4 (152.9, 321.5)	144.3 (105.9, 207.4)	96.9 (70.5, 132.8)	365.4 (245.7, 515.3)	220.2 (148.2, 308.1)	35.8 (-8.8, 102.9)	75.8 (60.9, 93.7)	7.0 (-21.3, 51.2)	-3.6 (-9.1, 3.1)
Saudi Arabia	17851.0 (12533.8, 27382.6)	127.1 (93.7, 169.3)	32812.3 (21647.3, 47099.8)	262.2 (174.8, 368.3)	33643.8 (24654.1, 45210.7)	123.8 (91.7, 159.1)	84595.6 (54922.6, 121556.9)	252.6 (168.2, 356.4)	88.5 (26.7, 191.1)	157.8 (131.4, 186.2)	-2.6 (-30.0, 41.3)	-3.6 (-8.8, 1.5)
Senegal	4818.5 (2918.3, 6423.3)	63.5 (38.9, 81.0)	14222.9 (9658.8, 19828.5)	243.4 (164.8, 337.9)	6284.8 (4301.0, 8596.0)	49.1 (34.7, 64.2)	28578.3 (19153.2, 39743.9)	236.0 (160.0, 327.2)	30.4 (-15.8, 113.4)	100.9 (88.3, 113.7)	-22.6 (-45.5, 17.7)	-3.1 (-8.2, 1.7)
Serbia	6317.9 (4810.8, 8412.5)	77.0 (56.6, 108.0)	26371.4 (17510.6, 37297.1)	254.4 (168.9, 359.6)	4081.0 (2809.4, 5223.8)	40.3 (28.4, 51.6)	29342.8 (19882.7, 40904.4)	275.3 (184.9, 387.0)	-35.4 (-56.9, -9.3)	11.3 (4.2, 20.0)	-47.7 (-67.5, -19.5)	8.2 (1.3, 16.3)
Seychelles	51.4 (30.8, 61.0)	78.8 (48.0, 93.7)	90.1 (60.0, 126.2)	146.5 (96.4, 207.0)	70.2 (54.7, 91.0)	66.2 (52.3, 86.8)	155.0 (100.6, 222.6)	128.4 (84.0, 182.6)	36.5 (6.0, 126.1)	72.0 (56.1, 87.7)	-16.0 (-34.2, 36.7)	-12.4 (-18.0, -6.6)

Sierra Leone	2667.1 (1434.9, 4228.0)	61.3 (35.8, 87.4)	6246.2 (4217.6, 8791.3)	219.6 (148.8, 308.1)	4355.9 (2636.6, 6587.5)	52.6 (34.4, 75.7)	13811.5 (9248.4, 19480.9)	217.7 (146.3, 308.0)	63.3 (5.7, 168.8)	121.1 (105.6, 138.3)	-14.2 (-40.8, 28.2)	-0.9 (-6.6, 4.9)
Singapore	1197.2 (865.5, 1372.8)	43.9 (32.3, 50.8)	8610.2 (5720.9, 12324.5)	280.3 (187.6, 397.0)	1314.8 (1083.8, 2227.3)	23.0 (18.6, 38.1)	20298.8 (13332.3, 28694.3)	284.3 (187.5, 403.0)	9.8 (-14.6, 107.1)	135.8 (117.5, 156.8)	-47.6 (-60.2, -2.9)	1.4 (-3.7, 7.2)
Slovakia	1835.7 (1566.7, 2431.9)	37.5 (31.8, 49.2)	23561.0 (15981.9, 32765.0)	424.6 (286.3, 592.9)	1896 (1257.7, 2532.2)	35.1 (22.8, 46.9)	26741.7 (18330.8, 36744.0)	391.0 (267.5, 545.5)	3.3 (-32.9, 44.6)	13.5 (6.9, 22.6)	-6.4 (-39.5, 29.9)	-7.9 (-13.1, -0.6)
Slovenia	1020.1 (787.2, 1284.1)	58.4 (45.5, 72.0)	6969.3 (4748.8, 9620.3)	323.2 (218.8, 449.9)	1425.6 (985.1, 1860.3)	61.2 (45.4, 80.1)	8624.8 (5906.5, 11958.5)	318.8 (217.5, 446.9)	39.8 (1.8, 84.5)	23.8 (17.4, 30.7)	4.7 (-18.0, 31.7)	-1.4 (-6.3, 3.9)
Solomon Islands	296.7 (210.1, 403.7)	130.2 (95.2, 174.8)	395.7 (265.1, 555.1)	160.1 (108.4, 223.8)	717.0 (485.5, 991.4)	145.9 (101.2, 195.6)	825.7 (555.3, 1165.4)	157.4 (105.6, 220.0)	141.7 (80.1, 221.4)	108.7 (93.6, 126.2)	12.1 (-18.3, 50.7)	-1.7 (-7.0, 4.8)
Somalia	3122.4 (1744.3, 4648.8)	48.6 (29.1, 73.5)	12204.5 (8192.9, 17142.7)	239.3 (162.0, 334.1)	8031.8 (4551.1, 15160.6)	50.7 (29.2, 103.3)	31838.2 (21469.0, 44953.7)	231.4 (157.1, 322.6)	157.2 (62.0, 318.4)	160.9 (145.8, 176.3)	4.3 (-29.7, 56.0)	-3.3 (-7.8, 0.6)
South Africa	74135.8 (53339.4, 85132.2)	221.7 (166.6, 255.3)	106421.8 (71287.2, 149654.1)	369.2 (250.4, 511.3)	115065.6 (91994.2, 141871.0)	215.7 (173.3, 261.9)	189390.2 (127305.0, 265997.2)	358.1 (241.6, 497.0)	55.2 (31.5, 106.2)	78.0 (71.4, 84.7)	-2.7 (-14.9, 20.6)	-3.0 (-5.0, -0.9)
South Sudan	2724.3 (1466.6, 4289.3)	44.0 (25.7, 61.7)	10121.2 (6742.7, 14209.3)	246.0 (165.1, 339.9)	3921.4 (2297.3, 5782.3)	47.0 (28.4, 69.3)	16523.7 (10983.4, 23170.8)	244.4 (163.7, 341.2)	43.9 (-4.9, 117.3)	63.3 (51.8, 75.5)	6.8 (-20.5, 47.7)	-0.7 (-5.8, 4.9)

Spain	27688.9 (22911.3, 36111.5)	75.6 (60.8, 94.2)	131626.9 (88334.0, 183535.5)	293.5 (196.2, 409.4)	51241.4 (36106.6, 58306.1)	87.0 (63.9, 100.5)	182393.1 (121508.9, 258897.2)	290.6 (192.3, 411.2)	85.1 (19.7, 107.9)	38.6 (30.0, 47.1)	15.1 (-18.1, 30.3)	-1.0 (-6.1, 4.0)
Sri Lanka	7056.5 (5480.4, 8313.9)	48.4 (37.4, 56.6)	22251.4 (14902.8, 30894.7)	145.6 (97.8, 201.1)	7713.1 (5377.9, 10475.1)	34.1 (23.9, 46.5)	32380.2 (21459.1, 45826.5)	130.6 (86.4, 183.0)	9.3 (-19.8, 53.4)	45.5 (31.8, 59.8)	-29.6 (-48.4, -2.3)	-10.3 (-17.1, -3.8)
Sudan	27221.2 (12054.7, 53458.2)	90.3 (48.0, 159.1)	40215.0 (27043.6, 57385.2)	247.8 (167.1, 351.2)	51570.4 (28338.9, 82827.2)	119.3 (69.3, 182.3)	79772.6 (53293.9, 114044.5)	237.8 (158.7, 334.5)	89.4 (-1.1, 276.4)	98.4 (86.8, 110.9)	32.2 (-27.6, 122.8)	-4.0 (-8.6, 1.2)
Suriname	859.2 (510.0, 1115.1)	225.8 (139.1, 286.5)	536.4 (356.6, 752.0)	169.6 (114.1, 236.9)	1000.5 (741.9, 1429.2)	184.2 (135.2, 261.9)	992.3 (664.9, 1404.5)	163.1 (109.9, 230.0)	16.4 (-23.5, 101.6)	85.0 (72.0, 97.4)	-18.4 (-46.3, 37.0)	-3.8 (-9.1, 1.7)
Sweden	5466.2 (3103.1, 6096.0)	50.7 (31.5, 55.9)	29501.5 (19790.6, 41064.5)	284.0 (189.9, 399.9)	6356.8 (4518.3, 7111.6)	42.9 (33.3, 51.3)	37936.5 (25312.9, 53599.7)	287.7 (191.5, 404.0)	16.3 (4.2, 56.3)	28.6 (21.4, 35.4)	-15.2 (-25.4, 21.5)	1.3 (-3.8, 6.6)
Switzerland	5846.4 (4777.6, 7120.3)	81.2 (67.1, 100.5)	19125 (13053.0, 26271.5)	224.3 (152.0, 309.0)	7784.7 (6295.1, 10268.1)	72.5 (59.6, 96.8)	28888.7 (19980.6, 40014.4)	230.1 (158.0, 319.1)	33.2 (15.9, 66.8)	51.1 (45.5, 57.0)	-10.7 (-24.3, 15.3)	2.6 (-1.2, 6.3)
Syrian Arab Republic	21312.1 (12750.9, 32595.3)	120.1 (81.1, 170.0)	24322.7 (16355.1, 34587.6)	243.3 (163.5, 341.4)	9646.1 (7034.7, 13199.5)	76.2 (55.7, 104.2)	33769.9 (22436.9, 47674.2)	233.2 (154.9, 326.6)	-54.7 (-71.6, -17.5)	38.8 (24.9, 53.8)	-36.5 (-57.3, 1.2)	-4.2 (-9.5, 1.2)
Taiwan (Province of China)	11547.5 (8699.4, 12782.1)	65.8 (49.4, 72.3)	37092.6 (24518.7, 51928.3)	195.9 (129.1, 274.4)	16236.3 (11886.0, 23692.2)	55.3 (40.7, 81.4)	49615.8 (33276.0, 69912.6)	160.8 (108.2, 223.7)	40.6 (5.9, 103.5)	33.8 (20.5, 46.0)	-16.0 (-36.7, 22.0)	-17.9 (-25.3, -10.4)

Tajikistan	1661.4 (1262.0, 2847.0)	24.3 (19.7, 37.6)	7596.4 (5131.3, 10789.4)	182.4 (123.2, 255.5)	3659.7 (2181.7, 4937.7)	37.4 (21.8, 50.1)	14499.1 (9749.4, 20540.0)	175.3 (118.4, 248.2)	120.3 (-6.4, 242.0)	90.9 (77.1, 106.3)	54.0 (-28.7, 125.4)	-3.9 (-9.6, 2.1)
Thailand	50084.1 (23763.5, 70760.6)	98.3 (49.4, 135.8)	63778.1 (41827.0, 90769.2)	129.2 (85.6, 180.7)	42335 (30366.9, 61141.0)	53.3 (38.7, 78.2)	116748.7 (76780.9, 168061.5)	124.1 (81.7, 177.0)	-15.5 (-52.2, 91.3)	83.1 (67.3, 100.9)	-45.8 (-68.5, 21.5)	-4.0 (-9.9, 2.8)
Timor-Leste	614.2 (255.1, 1127.1)	65.8 (39.7, 99.0)	727.0 (464.0, 1032.7)	127.9 (83.7, 179.6)	526.3 (346.6, 710.7)	45.4 (29.8, 62.4)	1282.1 (862.1, 1792.1)	122.4 (81.7, 171.2)	-14.3 (-54.7, 130.3)	76.4 (57.8, 110.7)	-30.9 (-57.8, 28.0)	-4.2 (-11.7, 6.9)
Togo	1566.5 (1031.0, 2146.1)	48.8 (30.9, 65.5)	5597.5 (3763.5, 7889.6)	220.4 (148.5, 307.8)	2688.4 (1910.3, 3641.7)	42.3 (30.7, 55.1)	13952.4 (9278.8, 19811.6)	222.4 (148.6, 312.7)	71.6 (17.2, 153.3)	149.3 (131.2, 167.0)	-13.2 (-38.4, 28.1)	0.9 (-4.9, 6.6)
Tokelau	1.5 (0.9, 2.1)	100.1 (63.2, 148.3)	2.5 (1.7, 3.5)	177.9 (119.1, 247.4)	1.3 (0.8, 1.8)	93.7 (60.6, 136.0)	2.2 (1.5, 3.1)	158.0 (105.4, 222.6)	-13.5 (-39.0, 21.2)	-13.8 (-19.4, -7.6)	-6.4 (-35.9, 33.0)	-11.2 (-16.0, -5.7)
Tonga	64.9 (46.3, 88.2)	91.1 (63.6, 125.6)	125.3 (85.0, 175.1)	168.3 (113.3, 232.9)	89.9 (60.6, 125.9)	102.1 (69.0, 143.2)	145.0 (97.2, 204.3)	161.1 (108.3, 227.4)	38.6 (2.6, 89.5)	15.7 (8.2, 23.7)	12.1 (-16.2, 53.5)	-4.3 (-9.7, 1.6)
Trinidad and Tobago	3753.1 (2679.8, 4187.1)	352.0 (250.8, 391.5)	1729.7 (1169.8, 2422.3)	172.0 (116.2, 241.1)	3883.0 (2658.0, 6446.2)	267.6 (180.9, 461.0)	2831.3 (1894.1, 3989.8)	168.4 (112.5, 234.7)	3.5 (-31.6, 97.5)	63.7 (50.5, 78.6)	-24.0 (-50.2, 53.7)	-2.1 (-7.5, 3.8)
Tunisia	8406.2 (5870.2, 12319.8)	87.8 (64.5, 123.8)	17276.6 (11454.8, 24810.4)	241.0 (160.0, 343.3)	7841.7 (5065.6, 10856.8)	72.8 (46.9, 100.4)	29825.7 (19616.7, 41587.3)	238.7 (158.0, 331.7)	-6.7 (-50.9, 47.9)	72.6 (60.0, 87.8)	-17.0 (-53.6, 28.2)	-0.9 (-6.3, 5.4)

Turkey	111859.4 (63449.4, 246153.0)	171.2 (102.2, 356.8)	142047 (94589.2, 200580.2)	274.6 (184.0, 384.4)	88414.9 (68239.9, 108311.2)	132.6 (101.2, 165.0)	256540.9 (169897.7, 361269.7)	292.0 (195.5, 411.2)	-21.0 (-62.2, 42.7)	80.6 (65.1, 96.6)	-22.5 (-61.1, 35.5)	6.3 (0.1, 14.5)
Turkmenistan	1680.9 (1259.8, 2031.6)	35.1 (28.1, 43.8)	5686.9 (3843.1, 7980.3)	194.0 (131.5, 273.8)	4197.8 (2861.8, 5311.8)	80.6 (55.3, 102.1)	9309.0 (6238.4, 13122.3)	188.7 (126.7, 264.3)	149.7 (91.5, 215.5)	63.7 (50.6, 77.0)	129.6 (68.9, 190.7)	-2.7 (-8.5, 3.0)
Tuvalu	10.8 (7.6, 14.0)	126.7 (88.8, 167.9)	15.2 (10.3, 21.2)	181.2 (122.6, 252.3)	11.7 (7.7, 16.6)	108.3 (71.4, 152.5)	18.0 (12.0, 25.1)	158.6 (106.0, 221.3)	8.7 (-22.3, 54.5)	17.9 (10.6, 25.8)	-14.5 (-38.6, 19.4)	-12.5 (-17.6, -7.0)
Uganda	5934.4 (3506.6, 8447.8)	34.2 (20.8, 49.2)	24312.5 (16564.2, 33806.0)	209.2 (141.4, 293.1)	12445.7 (7227.3, 19344.1)	39.5 (24.6, 56.4)	55631.9 (36855.0, 78521.4)	209.0 (140.1, 297.0)	109.7 (36.6, 225.4)	128.8 (112.5, 145.1)	15.3 (-14.3, 56.9)	-0.1 (-6.0, 6.4)
Ukraine	7999.0 (6896.2, 13317.5)	16.2 (13.8, 26.4)	91141.9 (61027.0, 128595.0)	155.3 (104.9, 219.6)	17840.9 (12236.2, 21294.1)	49.9 (30.4, 60.2)	80377.5 (53791.5, 112322.0)	150.3 (99.5, 213.6)	123.0 (20.1, 188.5)	-11.8 (-16.7, -5.5)	208.2 (47.4, 306.4)	-3.3 (-8.3, 3.5)
United Arab Emirates	1628.3 (1001.1, 2272.6)	101.3 (57.5, 140.8)	4126.8 (2691.5, 5943.4)	273.0 (180.6, 386.6)	9502.8 (4278.2, 17257.2)	127.3 (64.9, 207.3)	24136.4 (15484.8, 34916.8)	253.5 (167.9, 359.3)	483.6 (235.1, 911.5)	484.9 (409.9, 555.3)	25.7 (-17.1, 87.8)	-7.1 (-12.8, -1.1)
United Kingdom	60284.6 (43502.2, 63668.3)	99.0 (70.4, 105.5)	193815.4 (131255.6, 269085.9)	284.2 (191.8, 394.0)	62003.7 (54584.6, 89658.1)	84.6 (70.6, 114.6)	273893.4 (186627.8, 381025.7)	315.4 (212.8, 433.6)	2.9 (-7.5, 58.6)	41.3 (38.0, 44.8)	-14.5 (-23.4, 24.2)	11.0 (9.1, 13.1)
United Republic of Tanzania	10187.6 (7421.7, 13571.3)	38.1 (29.5, 47.9)	44200.4 (29790.4, 61851.7)	235.3 (157.6, 324.9)	22731.6 (15843.7, 32507.7)	44.9 (33.7, 59.5)	95370.3 (63769.8, 134554.0)	227.4 (153.3, 318.4)	123.1 (46.2, 235.1)	115.8 (102.0, 131.1)	17.8 (-11.9, 57.1)	-3.4 (-8.5, 1.9)

United States of America	272895.3 (226889.8, 362604.8)	102.5 (84.4, 134.9)	629499.9 (428397.4, 868359.1)	220.8 (150.0, 302.6)	772390.1 (598359.1, 893977.3)	182.0 (146.2, 221.5)	726711.0 (500661.1, 990691.1)	168.2 (115.1, 230.2)	183.0 (139.0, 197.3)	15.4 (6.8, 24.0)	77.5 (63.0, 84.2)	-23.8 (-29.2, -18.2)
United States Virgin Islands	232.2 (165.9, 294.2)	230.9 (165.7, 292.6)	176.4 (118.5, 252.0)	177.2 (119.9, 252.8)	255.4 (197.5, 327.1)	190.8 (146.9, 254.5)	258.0 (171.1, 370.9)	176.0 (118.0, 246.4)	10.0 (-21.8, 55.8)	46.3 (32.8, 60.6)	-17.4 (-42.2, 17.6)	-0.7 (-6.3, 5.5)
Uruguay	3719.0 (2489.7, 4132.4)	113.9 (77.1, 127.6)	9439.2 (6378.2, 13385.4)	274.4 (184.1, 387.6)	4151.7 (3586.6, 6028.6)	103.3 (87.4, 156.5)	11192.9 (7445.6, 16058.6)	267.7 (178.0, 378.4)	11.6 (-7.1, 80.6)	18.6 (12.4, 24.5)	-9.3 (-26.6, 53.5)	-2.5 (-7.5, 2.3)
Uzbekistan	4996.3 (4000.7, 6234.9)	20.1 (16.7, 26.2)	35309.0 (24050.1, 49664.8)	205.9 (140.0, 286.5)	10282.7 (8251.7, 16124.9)	32.0 (25.9, 48.4)	63160.6 (42581.3, 89096.6)	197.3 (133.6, 278.0)	105.8 (64.4, 173.5)	78.9 (65.3, 92.8)	58.9 (29.8, 99.3)	-4.2 (-9.7, 1.8)
Vanuatu	102.5 (67.9, 147.2)	99.1 (62.5, 146.9)	175.8 (118.6, 245.1)	159.8 (107.0, 224.4)	272.0 (178.9, 378.2)	119.0 (78.8, 168.3)	403.9 (273.1, 560.5)	165.7 (111.7, 230.1)	165.4 (86.2, 276.9)	129.8 (114.1, 147.0)	20.0 (-15.3, 74.6)	3.7 (-2.3, 10.4)
Venezuela (Bolivarian Republic of)	9581.3 (6953.1, 10747.5)	50.7 (38.5, 58.0)	22909.1 (15398.9, 32085.6)	175.3 (118.3, 246.6)	14821.4 (10613.5, 21622.2)	53.0 (37.9, 78.1)	48233.4 (32166.9, 67985.2)	162.9 (108.6, 229.3)	54.7 (9.2, 148.5)	110.5 (93.4, 127.9)	4.5 (-25.5, 57.0)	-7.1 (-12.2, -1.6)
Viet Nam	28708.5 (13784.0, 42655.1)	47.2 (23.3, 67.9)	68285.7 (45301.3, 95660.8)	132.3 (87.1, 186.1)	40653.6 (18957.5, 58442.2)	42.3 (20.9, 59.2)	121029.2 (79919.2, 173019.4)	111.0 (73.3, 156.7)	41.6 (-1.4, 112.7)	77.2 (60.8, 95.3)	-10.3 (-35.1, 29.2)	-16.0 (-22.0, -9.7)
Yemen	10949.2 (6276.9, 20144.5)	54.7 (37.9, 86.2)	27553.7 (18409.4, 39059.3)	255.5 (172.5, 359.5)	19454.5 (11142.9, 29629.1)	61.6 (36.5, 92.7)	71213.4 (48214.5, 100511.7)	268.9 (183.4, 376.8)	77.7 (-19.2, 276.5)	158.5 (141.8, 178.6)	12.6 (-43.8, 100.3)	5.2 (-0.2, 10.7)

Zambia	3173.8 (2150.7, 4522.7)	42.0 (31.0, 54.8)	13485.6 (9085.8, 18777.0)	244.0 (165.7, 337.7)	5177.5 (3727.0, 7110.0)	38.6 (29.3, 50.4)	33232.1 (22283.9, 46520.5)	247.7 (168.1, 343.0)	63.1 (2.5, 176.2)	146.4 (131.2, 162.3)	-8.2 (-34.9, 30.4)	1.5 (-3.4, 6.5)
Zimbabwe	5214.7 (2042.4, 7461.9)	55.8 (26.5, 72.7)	20577.3 (13876.0, 28948.6)	298.2 (200.8, 423.8)	3769.7 (2427.6, 6563.7)	29.0 (17.8, 56.7)	33097.7 (22310.7, 46469.1)	297.0 (202.0, 414.4)	-27.7 (-63.6, 147.5)	60.8 (50.0, 72.0)	-48.0 (-72.9, 65.2)	-0.4 (-5.9, 5.3)

EMBID: endocrine, metabolic, blood and immune disorders. **YLLs:** years of life lost. **YLDs:** years lived with disability.
