# Supplementary material for: Global, regional and national burden of endocrine, metabolic, blood and immune disorders 1990-2019: a systematic analysis of the Global Burden of Disease study 2019
Source: Front Endocrinol (Lausanne). 2023 May 8;14:1101627. doi: 10.3389/fendo.2023.1101627 (PMC10200867; doi:10.3389/fendo.2023.1101627)
Supplement: Supplementary file 2 [file DataSheet_2.pdf]

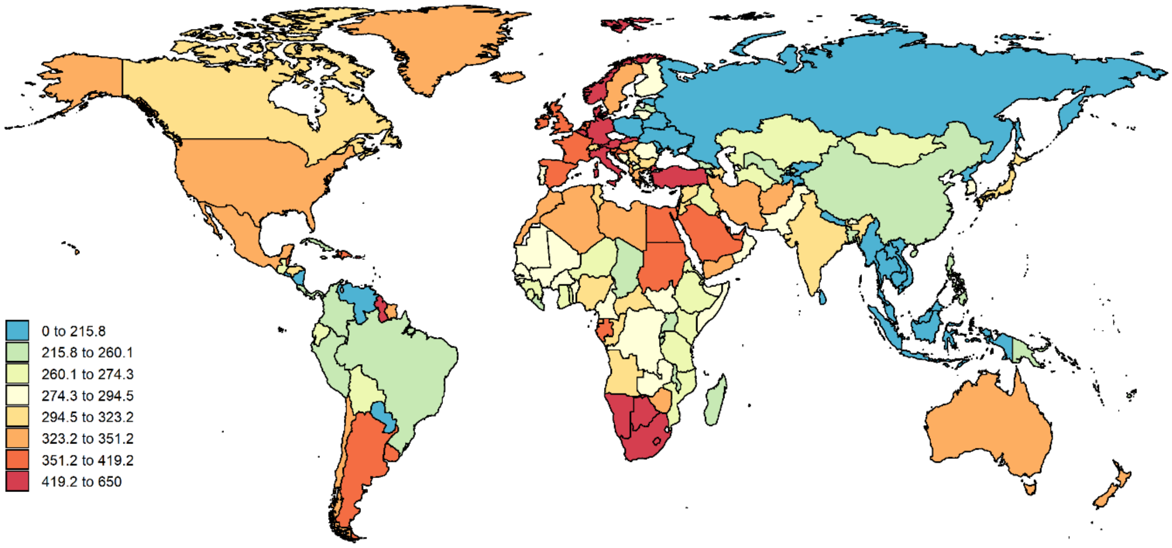

Figure S1: EMBID-related Age-standardized DALY rates by territory for both sex in 2019. **EMBID:** endocrine, metabolic, blood and immune disorders. **DALY:** disability-adjusted life year.

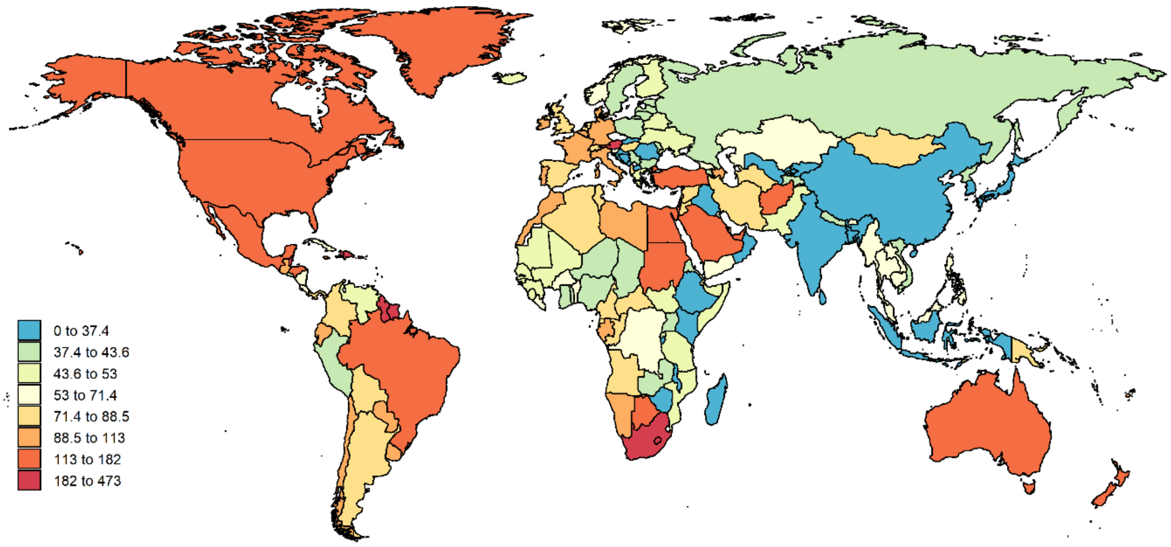

Figure S2: EMBID-related Age-standardized YLL rates by territory for both sex in 2019. **EMBID:** endocrine, metabolic, blood and immune disorders. **YLL:** year of life lost.

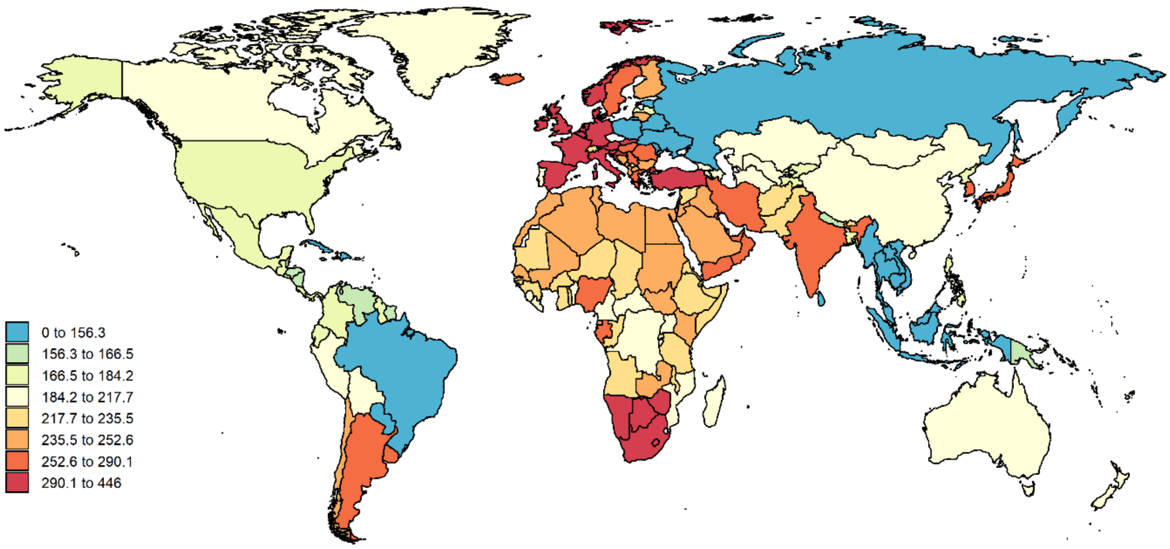

Figure S3: EMBID-related Age-standardized YLD rates by territory for both sex in 2019. **EMBID:** endocrine, metabolic, blood and immune disorders. **YLD:** year lived with disability.

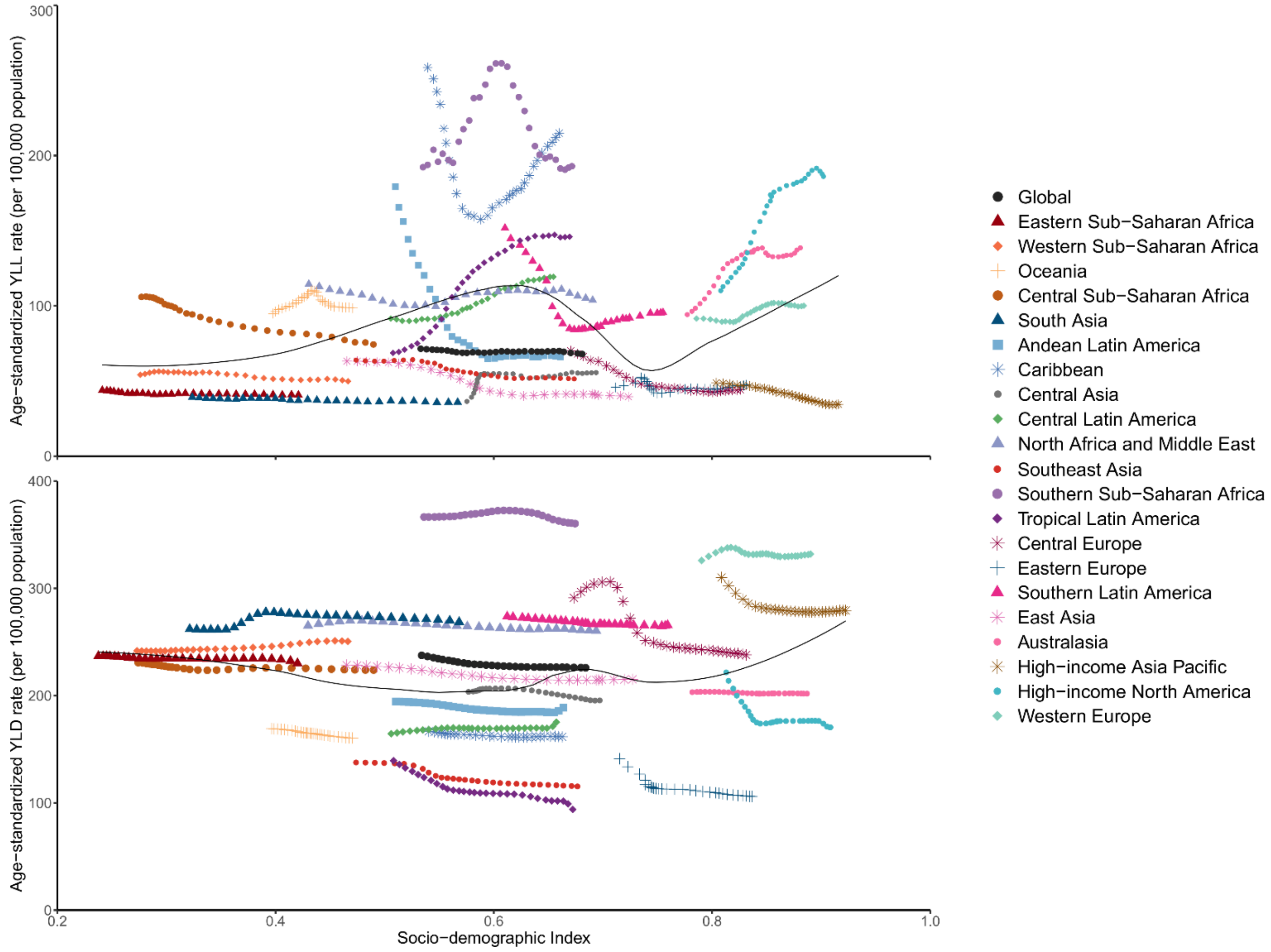

Figure S4: EMBID-related Age-standardized YLL rates and Age-standardized YLD rates across 21 GBD regions by Socio-demographic Index for both sexes combined, 1990-2019. **EMBID:** endocrine, metabolic, blood and immune disorders. **YLL:** year of life lost. **YLD:** year lived with disability.

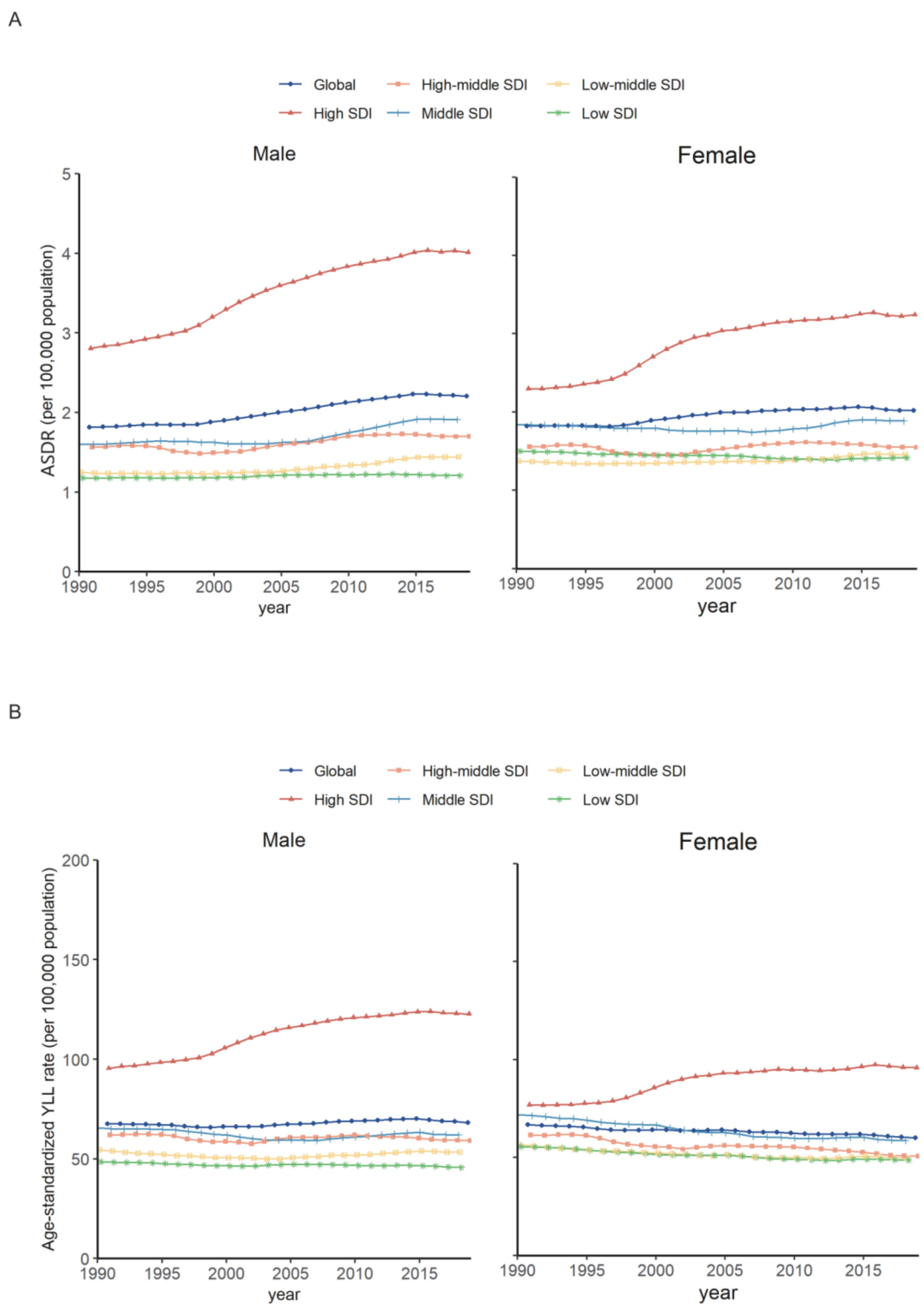

Figure S5: EMBID-related ASDRs (**A**) and Age-standardized YLL rates (**B**) for global and SDI by sex, from 1990 to 2019. **EMBID:** endocrine, metabolic, blood and immune disorders. **ASDRs:** age-standardized death rates. **YLL:** year of life lost.

A

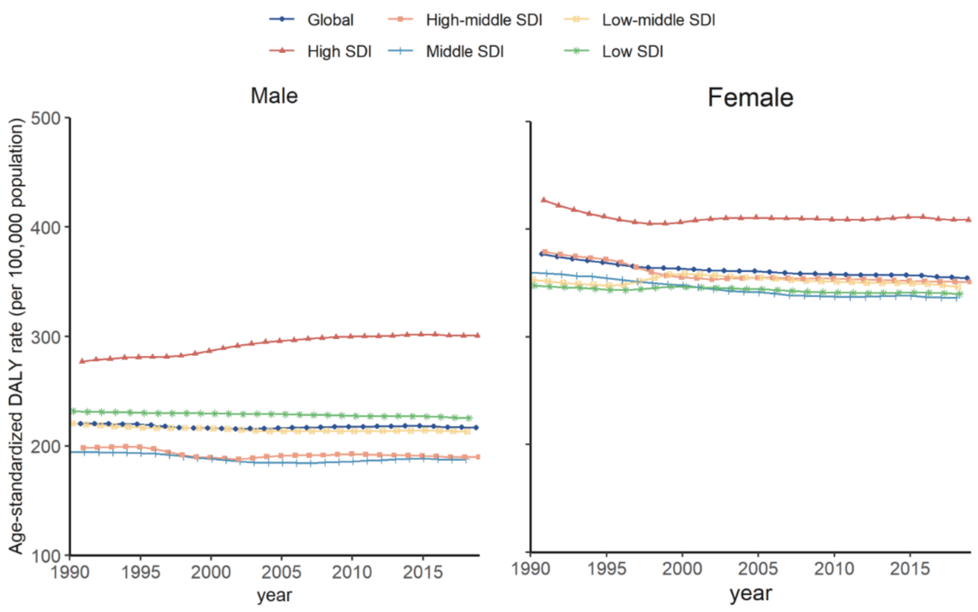

B

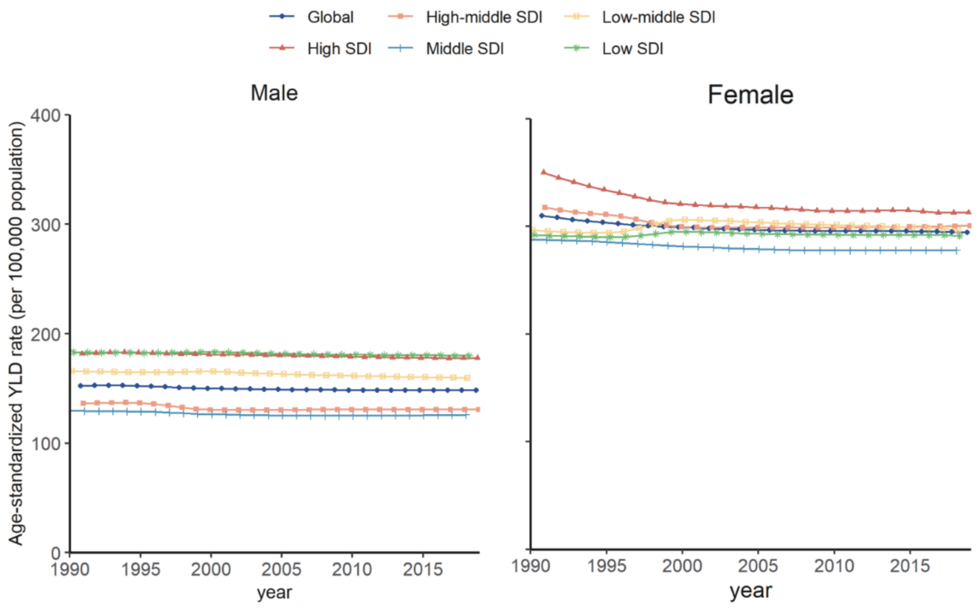

Figure S6: EMBID-related Age-standardized DALY rates (**A**) and Age-standardized YLD rates (**B**) for global and SDI by sex, from 1990 to 2019. **EMBID:** endocrine, metabolic, blood and immune disorders. **DALY:** disability-adjusted life year. **YLD:** year lived with disability.

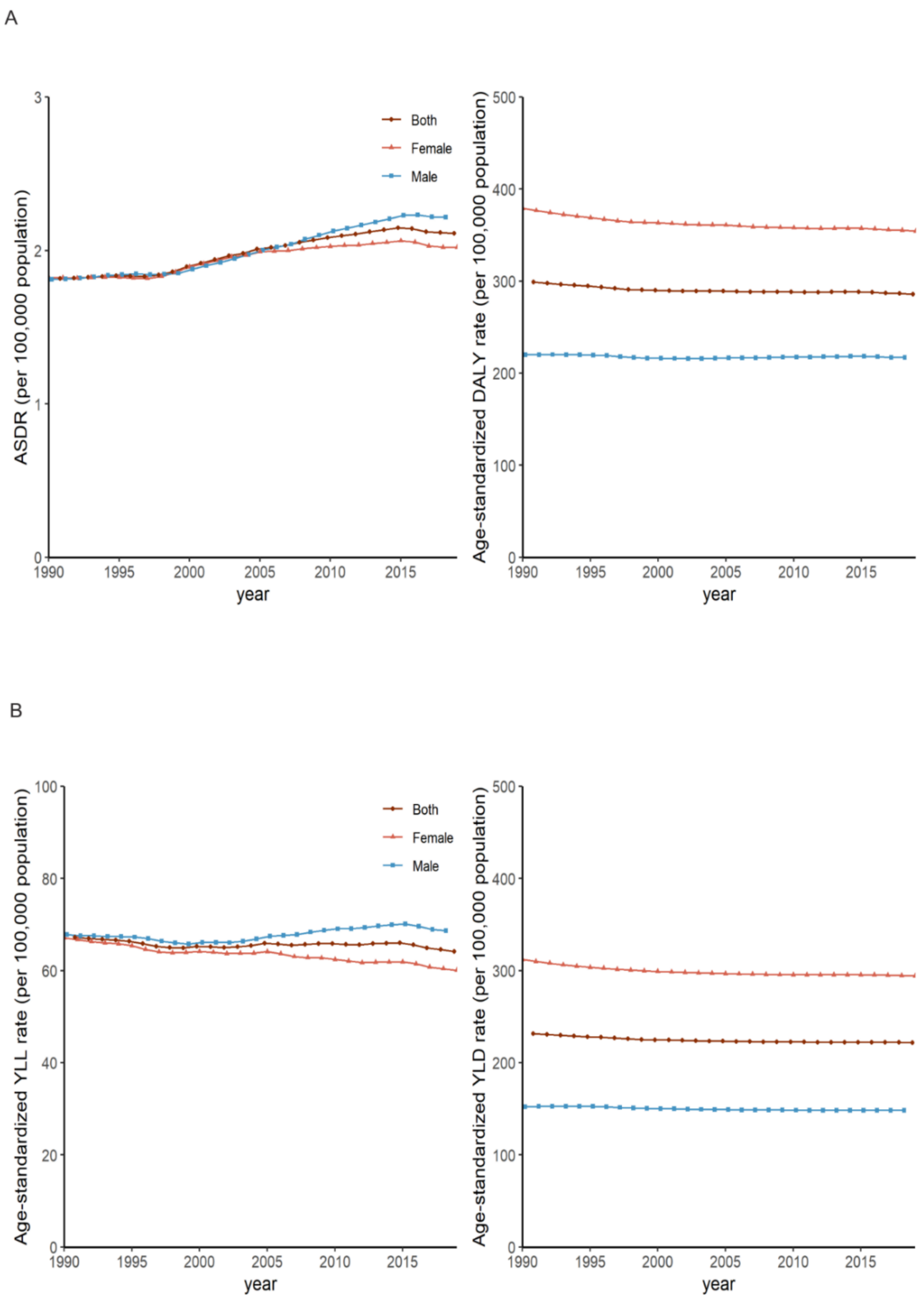

Figure S7: EMBID-related ASDRs, Age-standardized DALY rates (**A**), Age-standardized YLL rates and Age-standardized YLD rates (**B**) for global by sex, from 1990 to 2019. **EMBID:** endocrine, metabolic, blood and immune disorders. **ASDRs:** age-standardized death rates. **DALY:** disability-adjusted life year. **YLL:** year of life lost. **YLD:** year lived with disability.

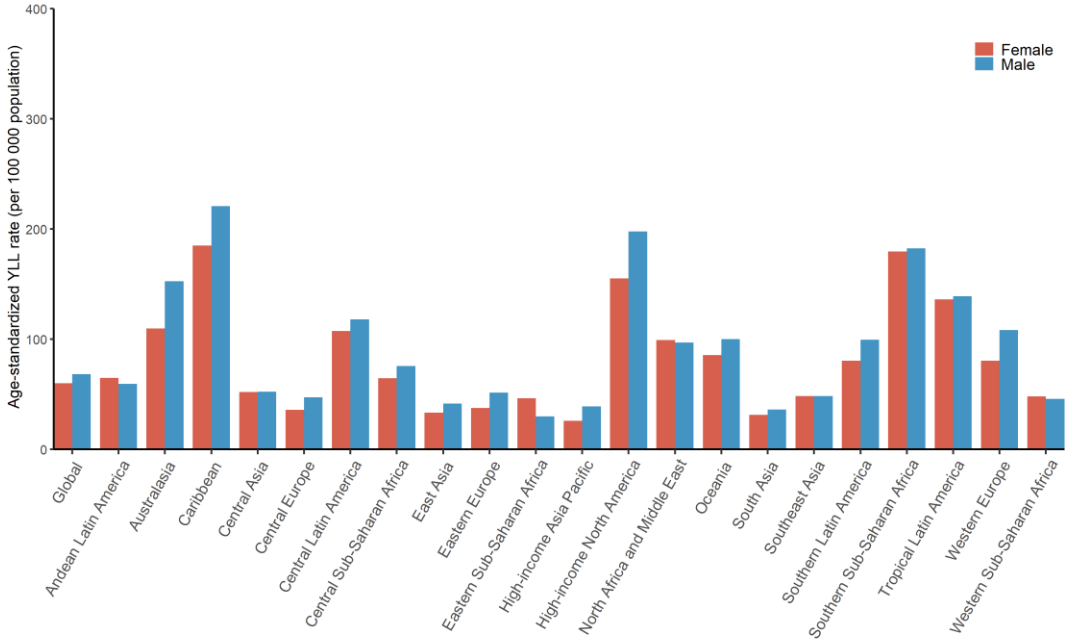

Figure S8: EMBID-related Age-standardized YLL rates for global and 21 GBD regions, by sex, in 2019. **EMBID:** endocrine, metabolic, blood and immune disorders. **YLL:** year of life lost.

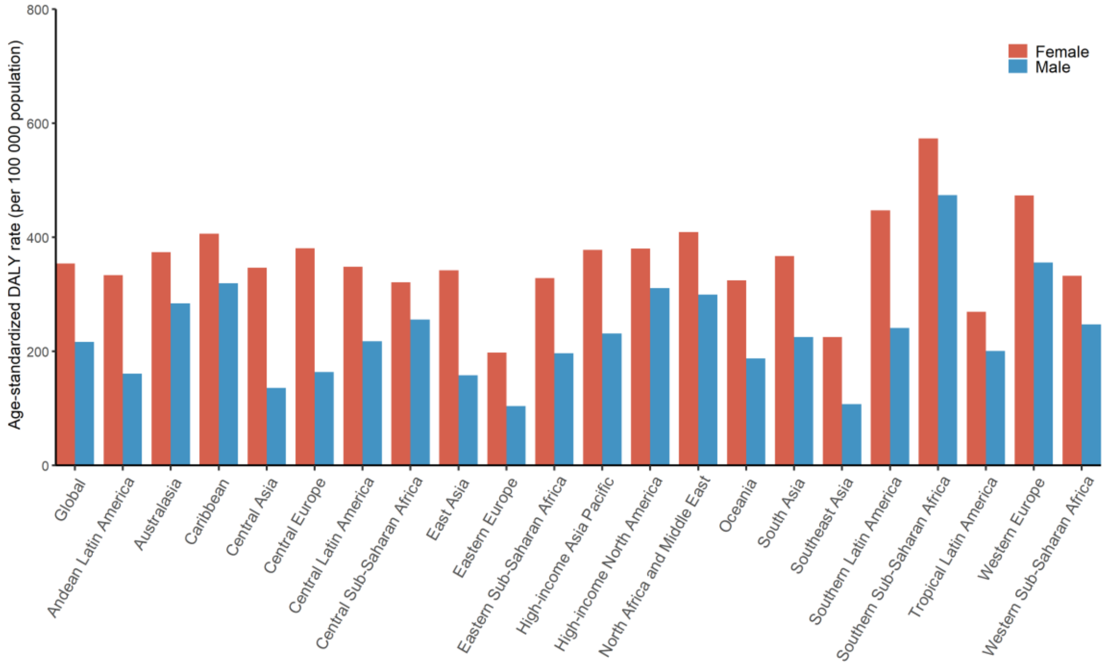

Figure S9: EMBID-related Age-standardized DALY rates for global and 21 GBD regions, by sex, in 2019. **EMBID:** endocrine, metabolic, blood and immune disorders. **DALY:** disability-adjusted life year.

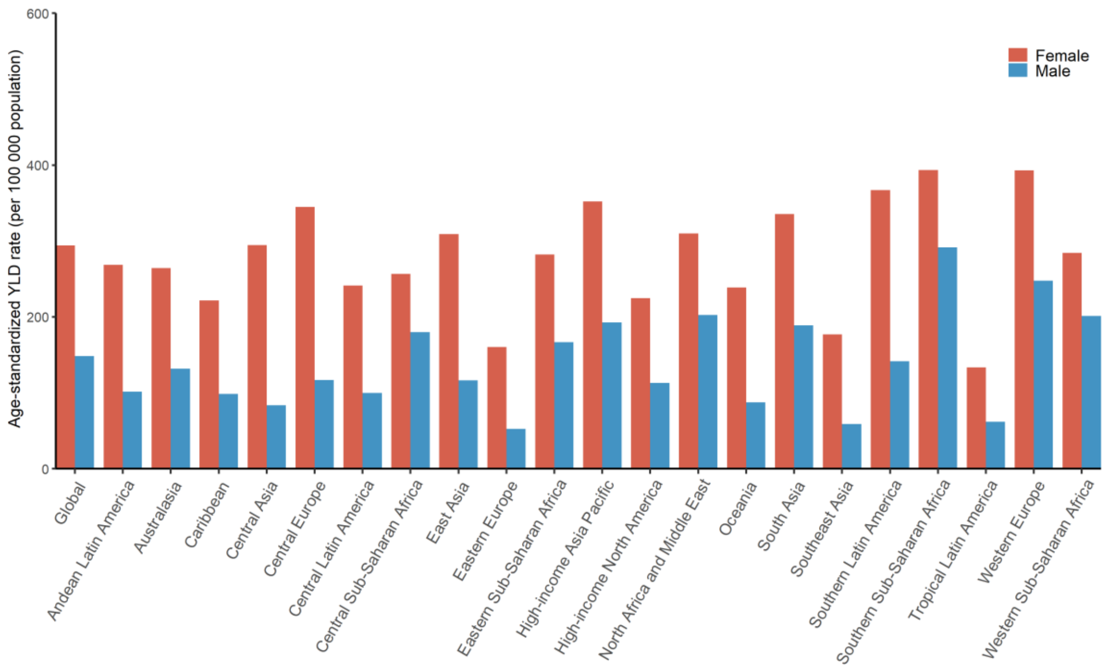

Figure S10: EMBID-related Age-standardized YLD rates for global and 21 GBD regions, by sex, in 2019. **EMBID:** endocrine, metabolic, blood and immune disorders. **YLD:** year lived with disability.

A

B

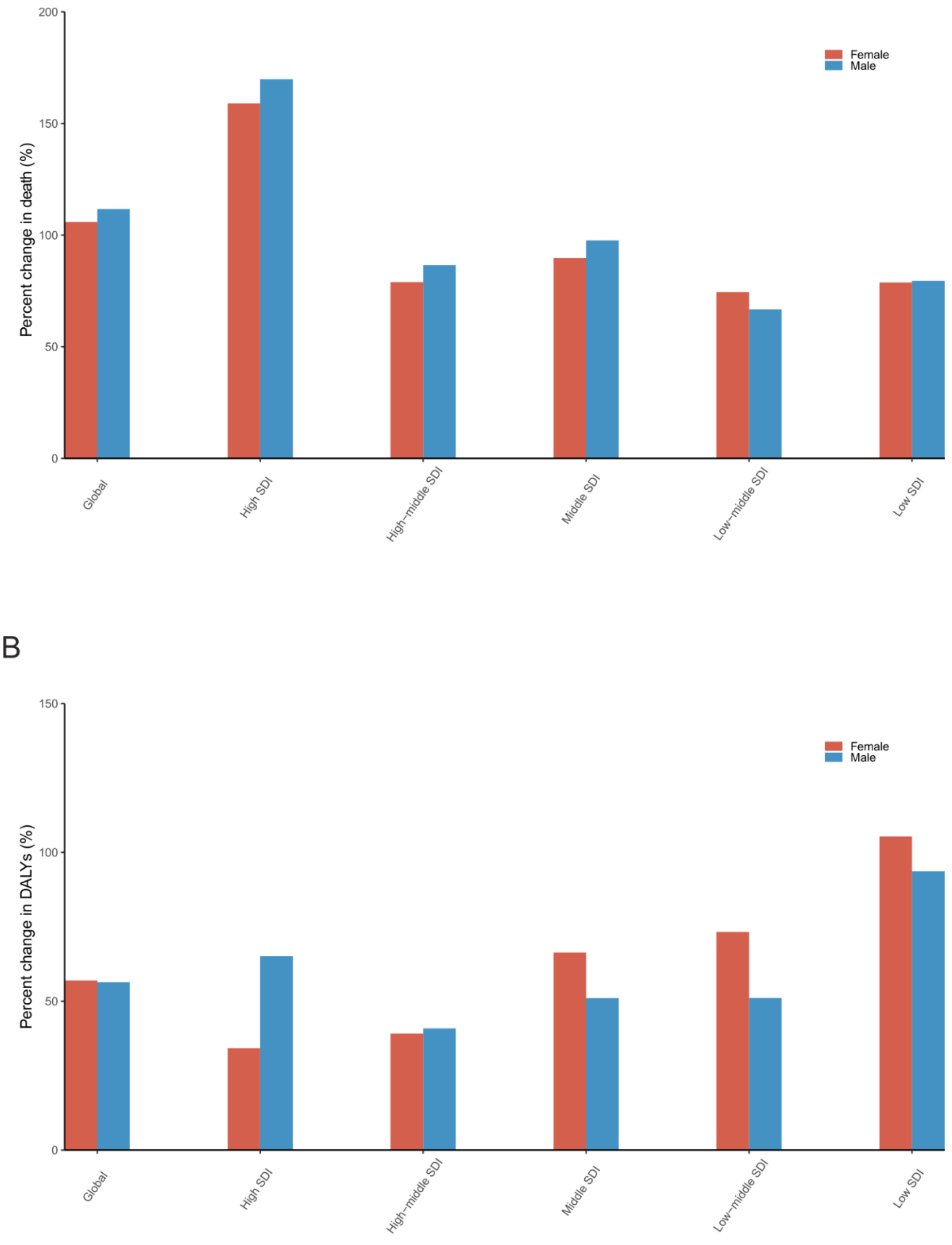

Figure S11: The percent changes of EMBID-related death cases and DALYs by global and SDI in males and females between 1990 and 2019. **EMBID:** endocrine, metabolic, blood and immune disorders. **DALYs:** disability-adjusted life years.

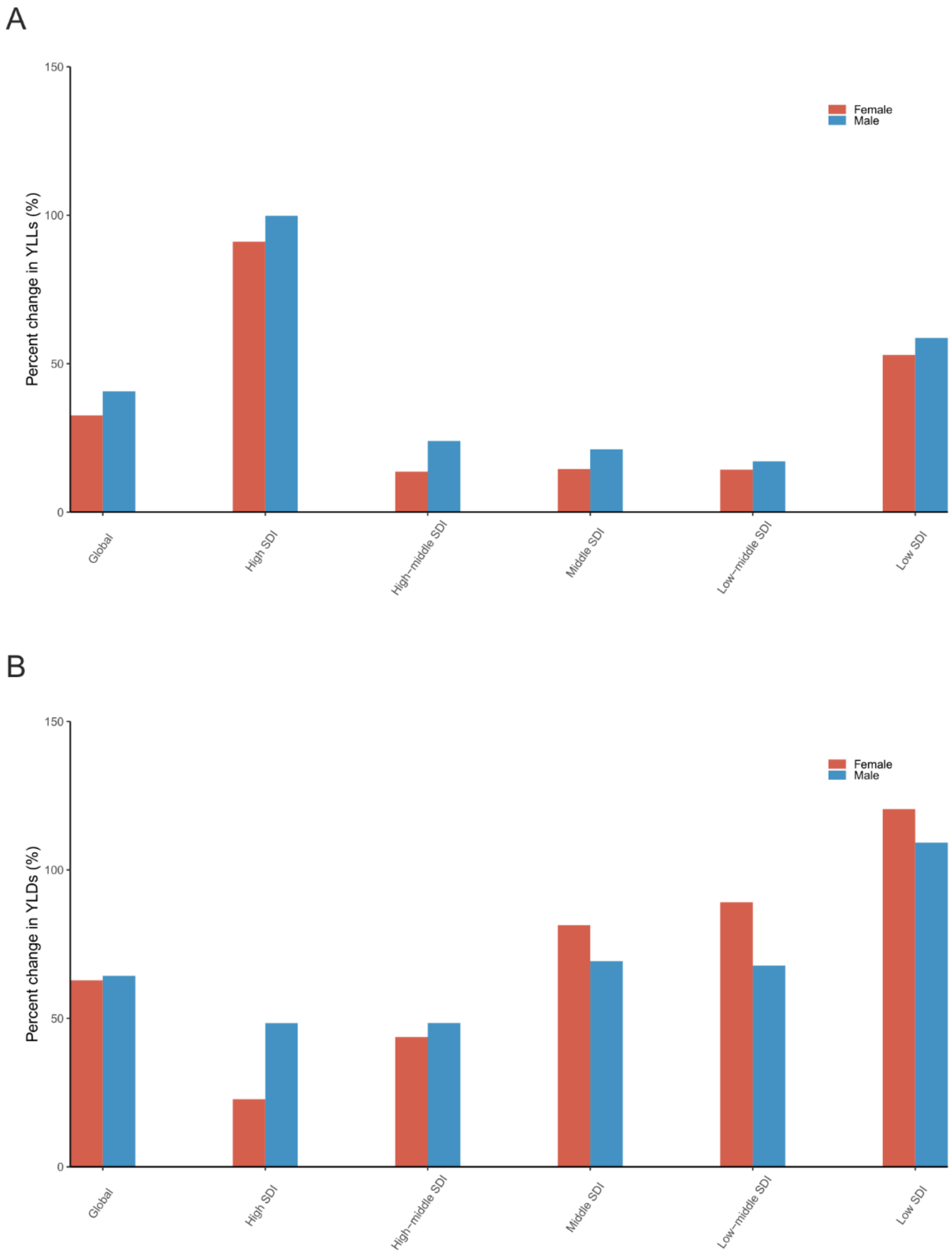

Figure S12: The percent changes of EMBID-related YLLs and YLDs by global and SDI in males and females between 1990 and 2019. **EMBID:** endocrine, metabolic, blood and immune disorders. **YLLs:** years of life lost. **YLDs:** years lived with disability.

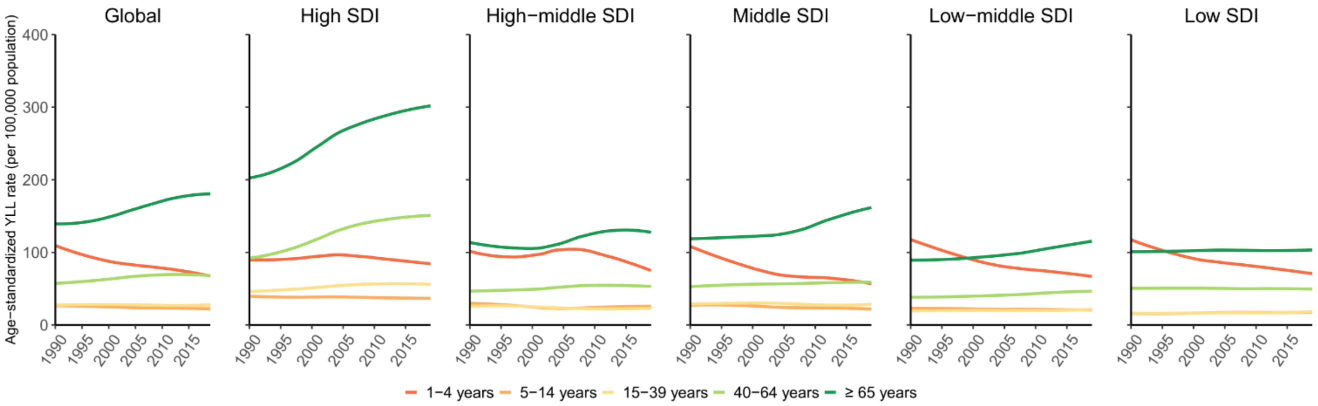

Figure S13: Global and SDI regions EMBID-related Age-standardized YLL rates by age, from 1990 to 2019. **EMBID:** endocrine, metabolic, blood and immune disorders. **YLL:** year of life lost.

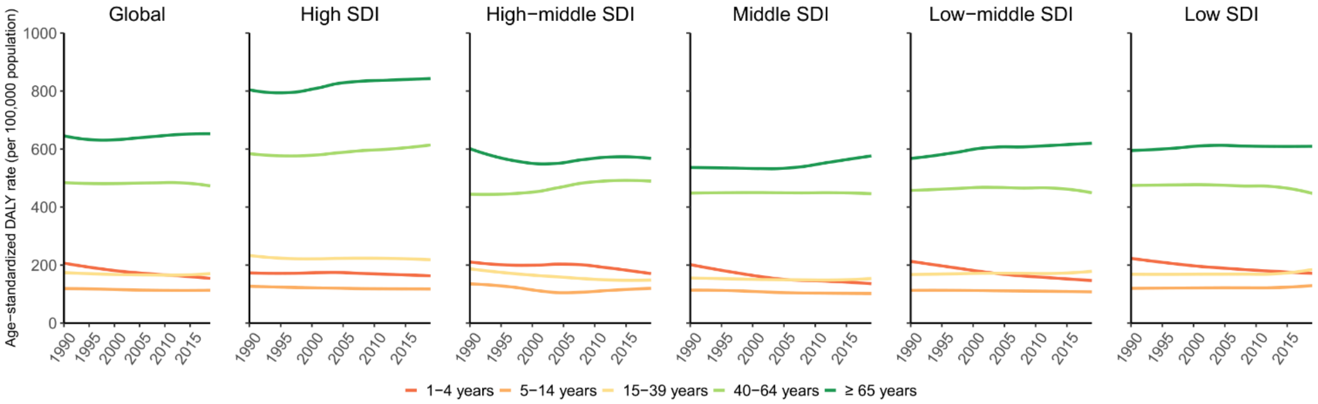

Figure S14: Global and SDI regions EMBID-related Age-standardized DALY rates by age, from 1990 to 2019. **EMBID:** endocrine, metabolic, blood and immune disorders. **DALY:** disability-adjusted life year.

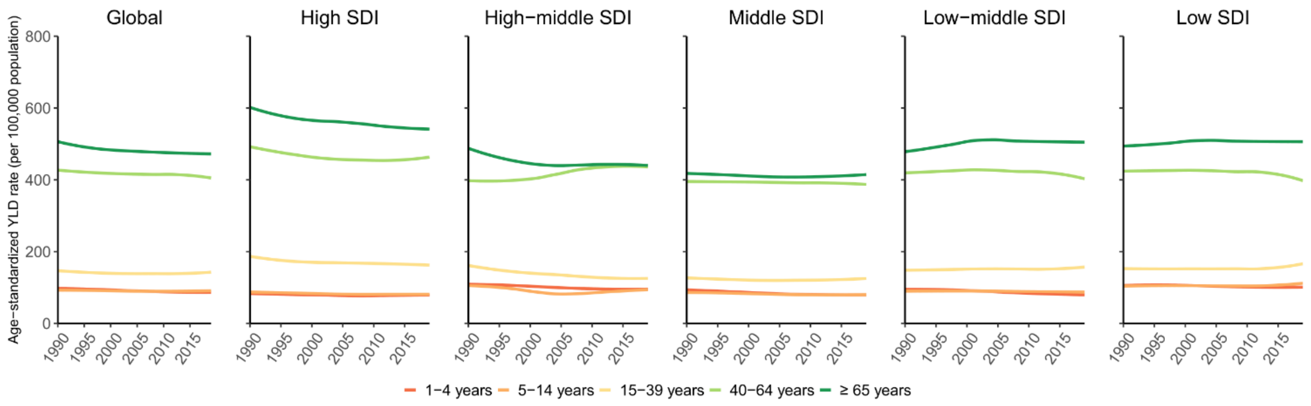

Figure S15: Global and SDI regions EMBID-related Age-standardized YLD rates by age, from 1990 to 2019. **EMBID:** endocrine, metabolic, blood and immune disorders. **YLD:** year lived with disability.

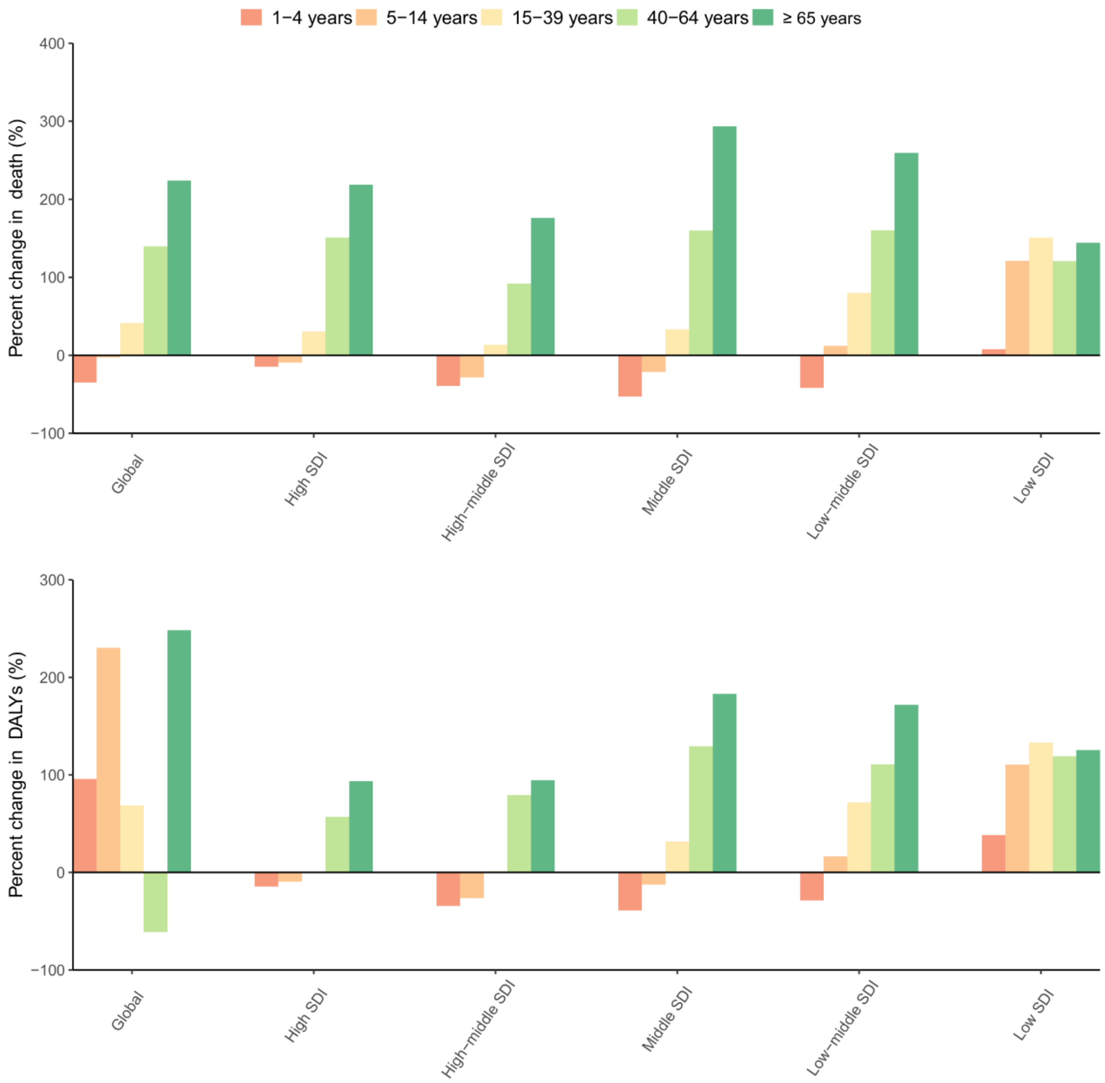

Figure S16: The percent changes of EMBID-related death cases and DALYs by age for global and SDI between 1990 and 2019. **EMBID:** endocrine, metabolic, blood and immune disorders. **DALYs:** disability-adjusted life years.

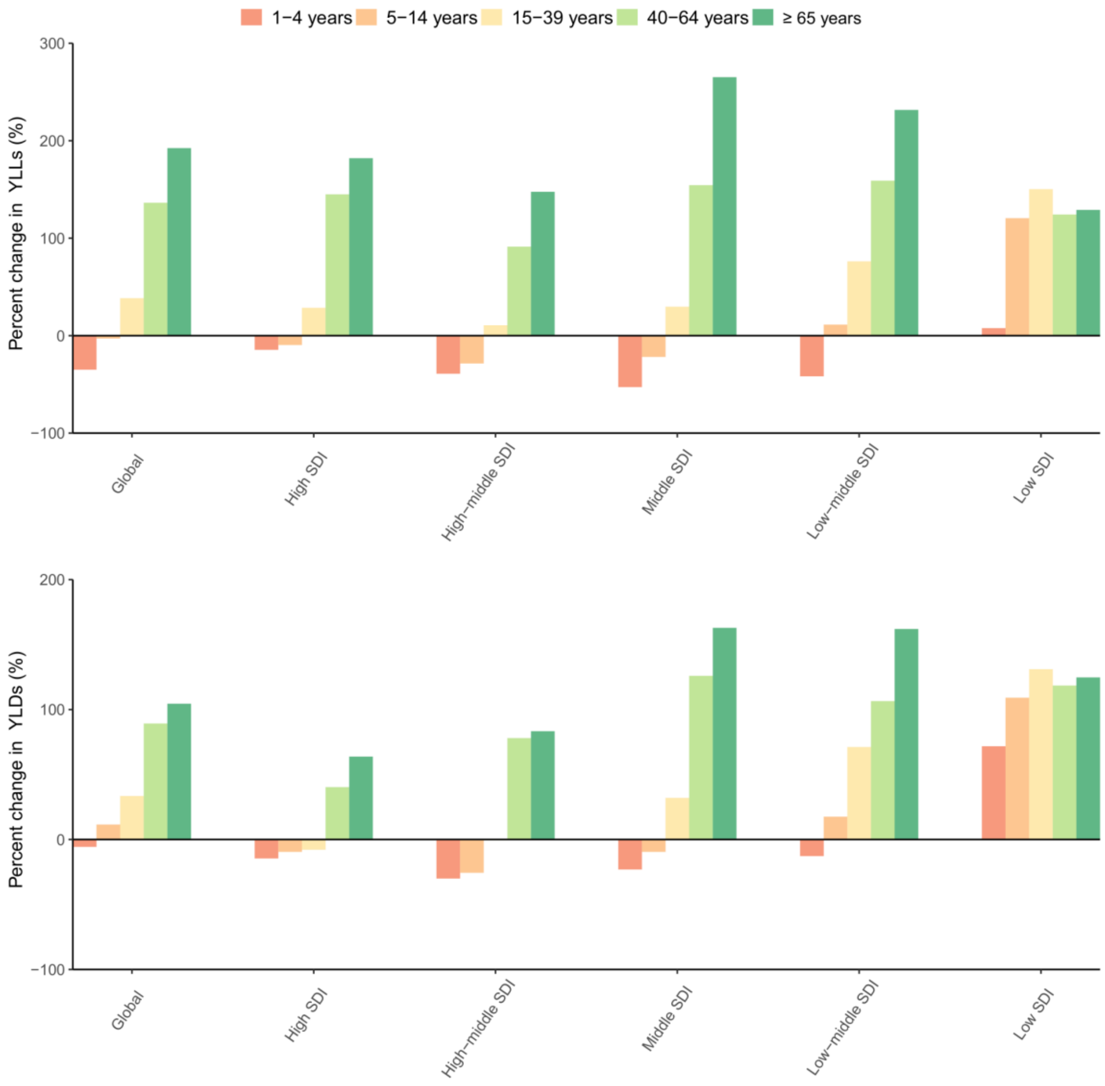

Figure S17: The percent changes of EMBID-related YLLs and YLDs by age for global and SDI between 1990 and 2019. **EMBID:** endocrine, metabolic, blood and immune disorders. **YLLs:** years of life lost. **YLDs:** years lived with disability.

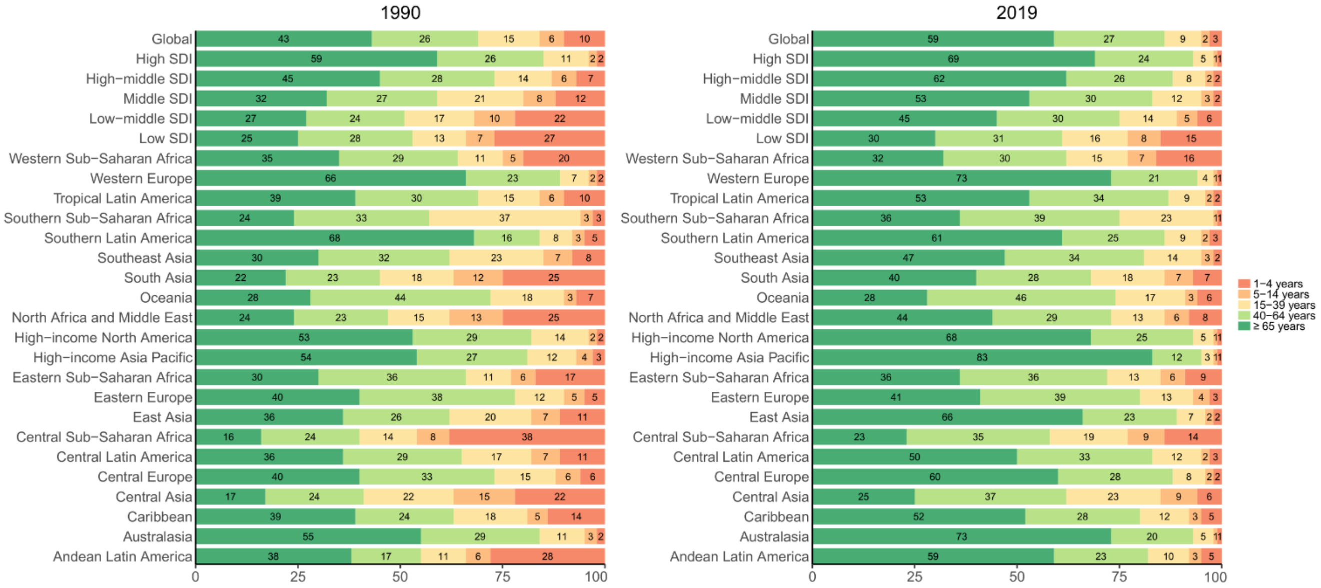

Figure S18: The five age groups as percentages of total EMBID-related death globally, in global, SDIs and 21 GBD regions in 1990 and 2019. **EMBID:** endocrine, metabolic, blood and immune disorders.

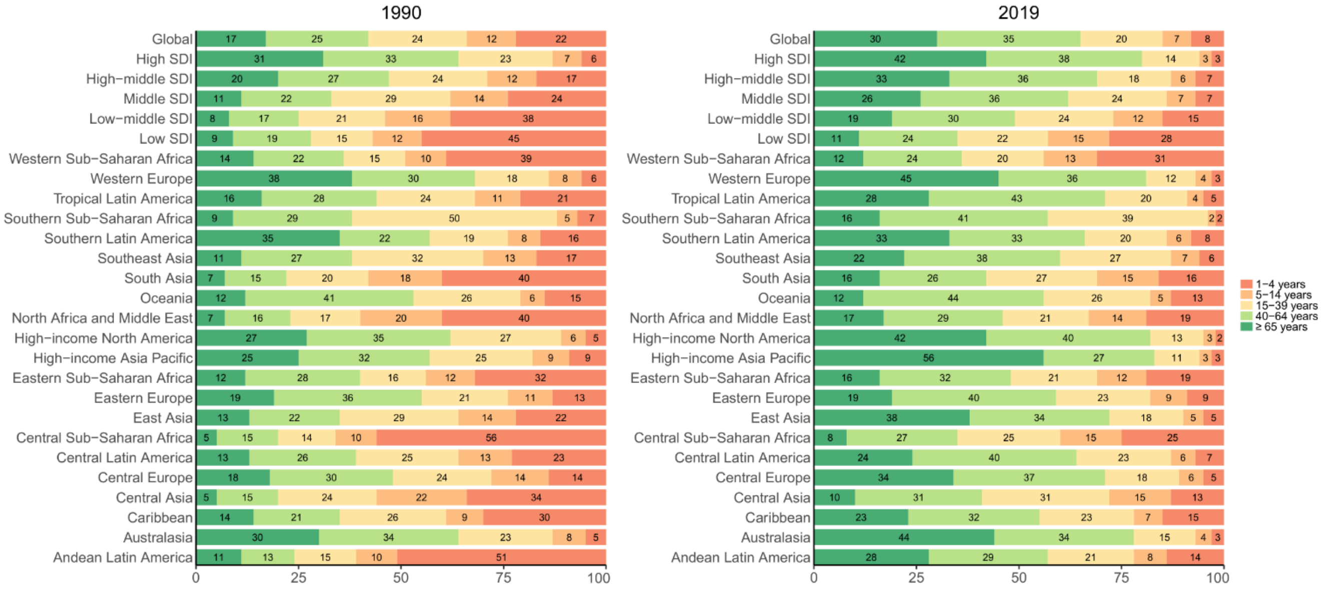

Figure S19: The five age groups as percentages of total EMBID-related DALYs globally, in global, SDIs and 21 GBD regions in 1990 and 2019. **EMBID:** endocrine, metabolic, blood and immune disorders. **DALYs:** disability-adjusted life years.

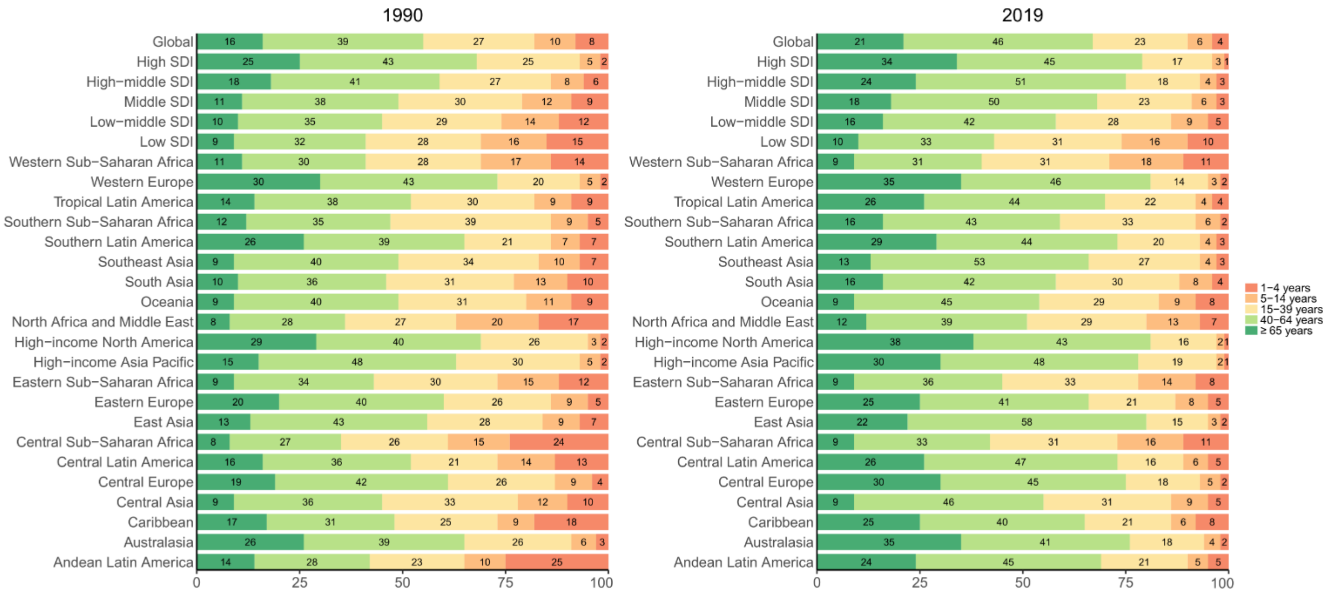

Figure S20: The five age groups as percentages of total EMBID-related YLLs globally, in global, SDIs and 21 GBD regions in 1990 and 2019. **EMBID:** endocrine, metabolic, blood and immune disorders. **YLLs:** years of life lost.

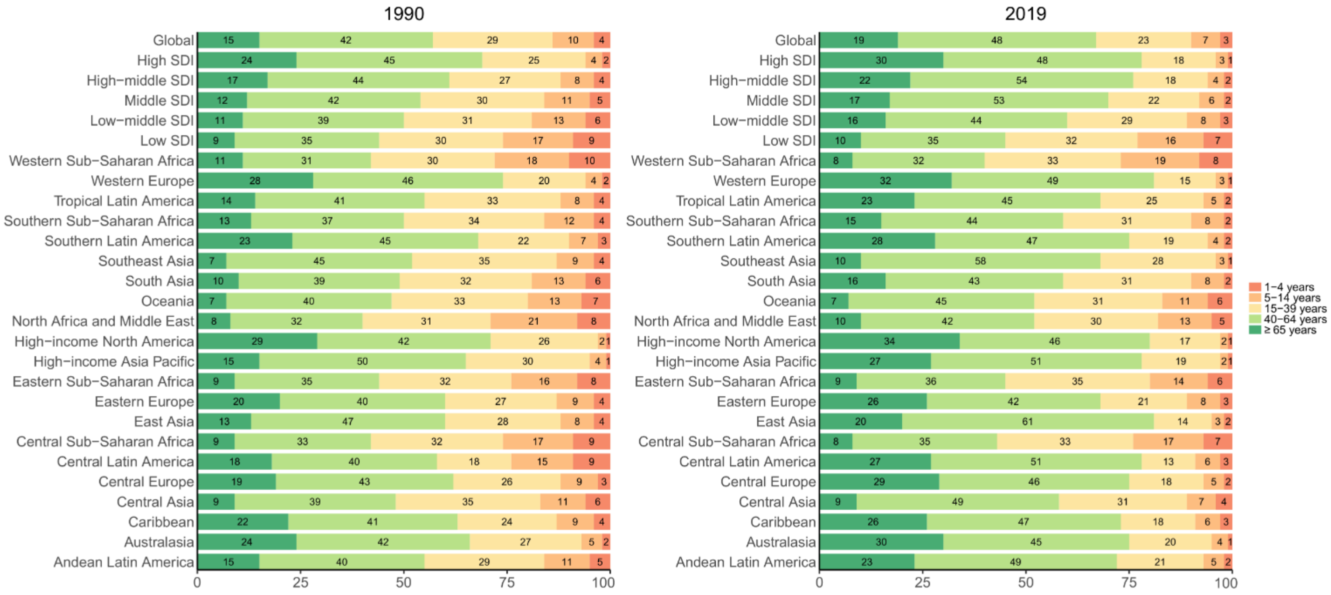

Figure S21: The five age groups as percentages of total EMBID-related YLDs globally, in global, SDIs and 21 GBD regions in 1990 and 2019. **EMBID:** endocrine, metabolic, blood and immune disorders. **YLDs:** years lived with disability.
